# Atypical *N*-Alkyl to *N*-Noralkoxy Switch in a Dual cSRC/BCR-ABL1 Kinase Inhibitor Improves Drug Efflux and hERG Affinity

**DOI:** 10.1021/acsmedchemlett.3c00479

**Published:** 2023-12-05

**Authors:** Jarvis Hill, Robert M. Jones, David Crich

**Affiliations:** † Department of Pharmaceutical and Biomedical Sciences, University of Georgia, Athens, Georgia 30602, United States; ‡ Department of Chemistry, University of Georgia, Athens, Georgia 30602, United States; § Independent Researcher, P.O. Box 568, Oakley, Utah 84055-0568, United States; ∥ Complex Carbohydrate Research Center, University of Georgia, Athens, Georgia 30602, United States

**Keywords:** Bioisostere, Hydroxylamine, BCR-ABL1, Leukemia, hERG, Efflux

## Abstract

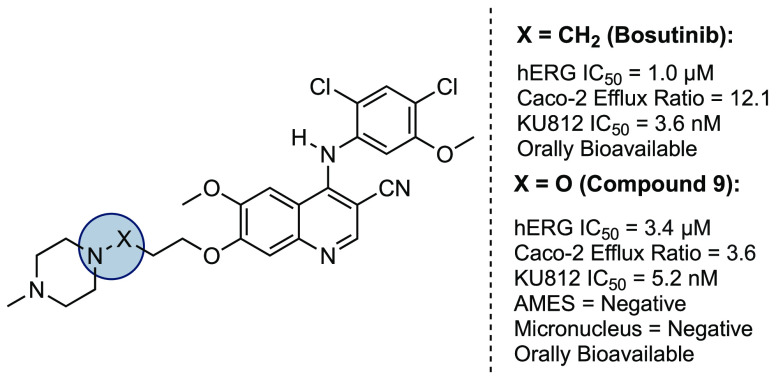

We describe an atypical amine bioisostere, the trisubstituted hydroxylamine, that upon incorporation into an approved dual cSRC/BCR-ABL1 kinase inhibitor yields **9**, a compound that retains potent biological activity and couples it with improved drug efflux and hERG affinity at the expense of only a 2 atomic mass unit increase in molecular weight. Contrary to the common expectation for hydroxylamines in medicinal chemistry, **9** is well tolerated *in vivo* and lacks the mutagenicity and genotoxicity so often ascribed to lesser substituted hydroxylamines. A matched molecular pair (MMP) analysis suggests that the beneficial properties conferred by the *N*-alkyl to *N*-noralkoxy switch arises from a reduction in basicity of the piperazine unit. Overall, these results lend additional support to the use of trisubstituted hydroxylamines as bioisosteres of *N*-alkyl groups that are not involved in key polar interactions.

We recently described compound **2**, a hydroxylamine-bearing, orally bioavailable, brain-penetrant, selective epidermal growth factor receptor (EGFR) inhibitor with potential for treating EGFR+ central nervous system (CNS) metastases in non-small-cell lung cancer (NSCLC) (Figure 1a).^1^ Key to the discovery of **2** was the recognition that derivatization of a saturated nitrogen heterocycle in the form of a hydroxylamine could attenuate basicity and modify the lipophilicity of the starting molecule, gefitinib (**1**), in such a way that **2** evades efflux by transporters, such as P-glycoprotein (P-gp, or MDR1), and/or breast cancer resistance protein (BCRP).^1−3^ Unlike lesser substituted hydroxylamines^4,5^ and structurally related hydroxamates,^6,7^ which confer mutagenicity by metabolic activation to electrophilic nitroso derivatives or isocyanates and subsequent conjugation with DNA, **2** exhibited good stability *in vitro* and *in vivo* and critically lacked both mutagenicity and genotoxicity.^8,9^ We now describe the extension of this novel bioisosteric design strategy to the FDA-approved small molecule drug, bosutinib (**3**).

**Figure 1:**
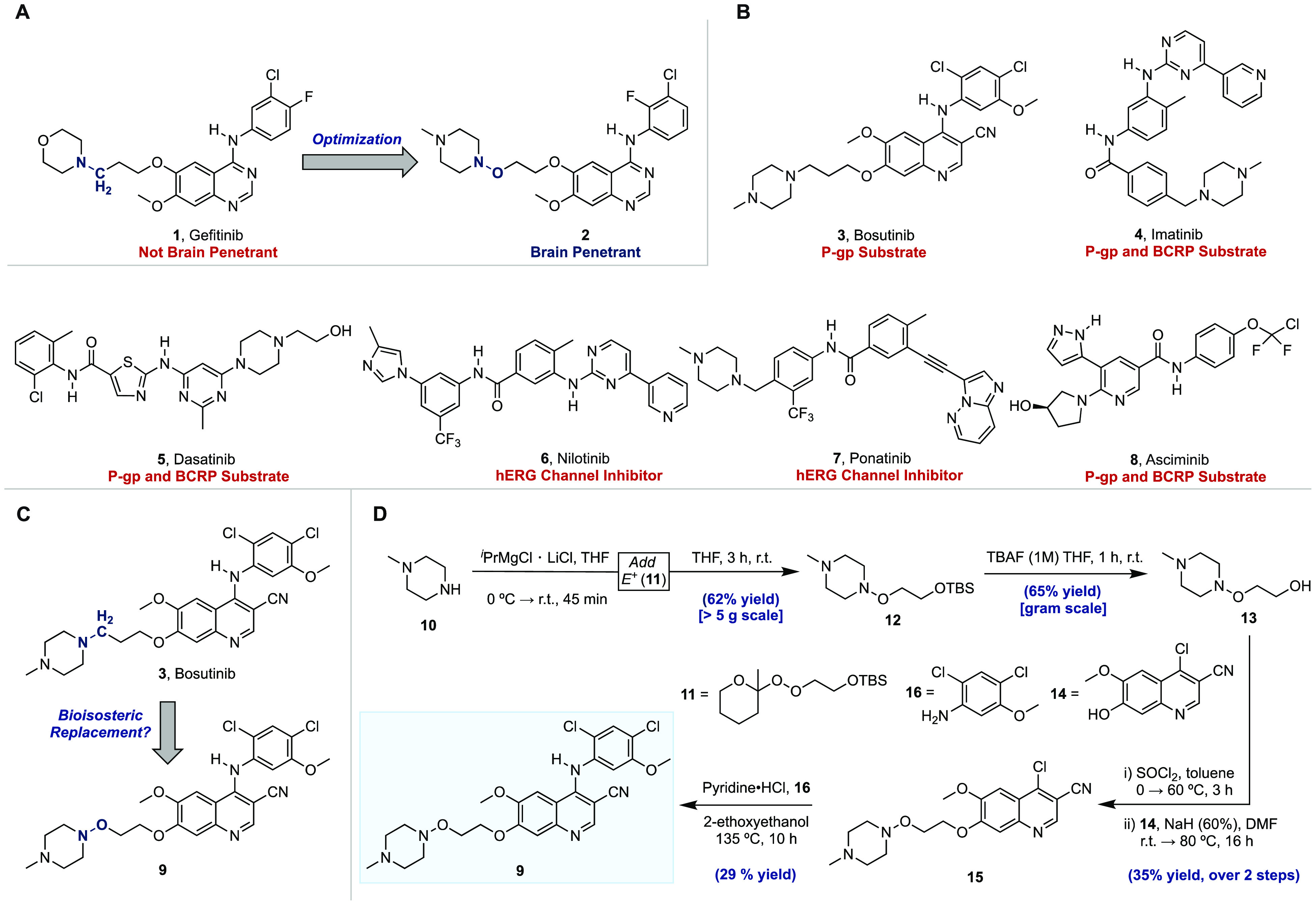
Overview of selected BCR-ABL1 inhibitors and the bioisosteric design strategy deployed in this study. (A) Chemical structures of gefitinib and the brain-penetrating EGFR inhibitor, **2**. (B) Chemical structures of select BCR-ABL1 inhibitors and their liabilities against efflux transporters or the hERG ion channel. (C) Proposed bioisosteric replacement. (D) Chemical synthesis of **9**. ^*i*^PrMgCl·LiCl, isopropylmagnesium chloride lithium chloride; THF, tetrahydrofuran; TBAF, tetra-*N*-butylammonium fluoride; SOCl_2_, thionyl chloride; NaH, sodium hydride; DMF, dimethylformamide; HCl, hydrogen chloride.

Bosutinib is approved for the treatment of chronic myeloid leukemia (CML), a disease that comprises 15–20% of all adult leukemia cases worldwide and that is characterized by a reciprocal translocation [t(9;22)(q34;q11)] of DNA between chromosomes 9 and 22 in hematologic progenitor cells, which results in fusion of the *ABL1* (Abelson proto-oncogene 1, chromosome 9) and *BCR* (breakpoint cluster region, chromosome 22) genes on the Philadelphia chromosome (Ph).^10−15^ The first BCR-ABL1-targeted tyrosine kinase inhibitor (TKI), imatinib (**4**), which received regulatory approval in 2001, ushered in a new era of targeted drug discovery and turned a once fatal leukemia into one in which the lifespan of patients now approaches that of the general population.^16−21^ Unfortunately, acquired resistance to imatinib (**4**), which appears in roughly 40% of all CML patients, is a significant challenge in management of the disease.^22−26^ The search for drugs to treat CML in all phases with acquired resistance to imatinib led to the discovery of second-generation BCR-ABL1 inhibitors, such as bosutinib (**3**),^15^ dasatinib (**5**),^27^ and nilotinib (**6**),^28^ and third-generation inhibitors ponatinib (**7**)^29^ and asciminib (**8**)^30^ (Figure 1b). Unfortunately, second- and third- generation BCR-ABL1 inhibitors nilotinib and ponatinib have serious potential side-effects due to inhibition of the human ether-à-go-go-related gene (hERG) potassium ion channel, which significantly limits their use in the treatment of CML.^31−35^ Indeed, nilotinib carries a black box warning for QT interval prolongation and sudden death, while ponatinib carries a black box warning for vascular occlusion, heart failure, and hepatotoxicity. While bosutinib, dasatinib, and asciminib have lower affinity for the hERG ion channel, these second- and third- generation BCR-ABL1 inhibitors are substrates for efflux transporters P-gp and/or BCRP, which can confer indirect inhibitor resistance through active drug efflux.^36−39^ Thus, developing potent inhibitors of BCR-ABL1 with minimal affinity for the hERG ion channel and reduced drug efflux is of significant interest for the clinical management of Ph+ CML. Building on our previous results,^1^ we hypothesized that a bioisosteric replacement of the *N*-alkyl piperazine unit in bosutinib by an equivalent hydroxylamine *N*-noralkoxy unit would attenuate the basicity^2^ and modify the lipophilicity^3^ of bosutinib to reduce hERG affinity and efflux propensity (Figure 1c). Herein, we reduce this hypothesis to practice and report on the direct incorporation of a trisubstituted hydroxylamine *N*-noralkoxy unit into a bosutinib *N*-alkyl unit that leads to both a reduction in hERG affinity and efflux propensity at the expense of an insignificant molecular weight increase of 2 atomic mass units (amu). These properties are all achieved while maintaining oral bioavailability and potent antiproliferative activity in patient-derived Ph+ leukemia cells and without the anticipated toxicity commonly surmised^8,9^ to accompany hydroxylamine units. Collectively, we provide additional support for the use of trisubstituted hydroxylamines as bioisosteres in medicinal chemistry.

The synthesis of **9** commenced with our direct N–O bond-forming reaction^40−42^ that gave multigram-scale quantities of hydroxylamine (**12**) in 62% yield upon reaction of the *N*-methylpiperazine (**10**)-derived magnesium amide with the 2-methyltetrahydropyranyl (MTHP) monoperoxyacetal^43−45^ (**11**) derived from commercially available 2-[(*tert*-butyldimethylsilyl)oxy]ethanol (Figure 1d). Deprotection of the *tert*-butyldimethylsilyl (TBS) group in **12** with tetra-*N*-butylammonium fluoride (TBAF) proceeded in 65% yield and was followed by chlorination and displacement with phenol (**14**) under basic conditions to give quinoline (**15**) in 35% yield over two steps. A nucleophilic aromatic substitution (S_N_Ar) reaction with aniline (**16**) under acidic conditions then gave **9** in 29% yield.

With **9** in hand, we initially evaluated biochemical inhibition of the relevant mutant BCR-ABL1 kinases and found that **9** maintained strong inhibition of imatinib-resistant BCR-ABL1 mutants. As expected,^46^ however, **9** showed reduced activity against BCR-ABL1^T315I^ that bears the T315I “gatekeeper” mutation, which disrupts the ATP binding region drug contact sites (Table 1). Moreover, **9** also strongly inhibited cSRC, an additional target of bosutinib with an IC_50_ value of 2.0 nM.

**Table 1:** Determination of Kinase Inhibition (IC_50_) of Bosutinib and **9** against Mutant BCR-ABL1 and cSRC

kinase	bosutinib^a^	**9**
BCR-ABL1 IC_50_(nM)^b^^,^^c^		
wt	<1	<1
H396P	<1	<1
M351T	<1	<1
Q252H	<1	<1
T315I	44	47
Y253F	<1	<1
cSRC IC_50_(nM)^b^^,^^c^		
wt	2	2

a Obtained from MedChemExpress (HY-10158).

b Conducted by Eurofins Cerep, SA.

c IC_50_ represents mean of *n* = 2 technical replicates conducted at [ATP] = 10 μM.

Moving to *in vitro* ADMET properties of **9**, we first measured lipophilicity, where **9** and bosutinib had log*D*_7.4_ values of 3.5 and 3.1, respectively (Table 2). Despite the increase in lipophilicity, **9** and bosutinib exhibited similar solubility at pH 7.4 and comparable unbound plasma protein fractions in human and rat. Both **9** and bosutinib showed significant degradation in human and rat liver microsomes, while the compounds exhibited comparably enhanced stability in hepatocytes. We then tested for permeability in colon-carcinoma (Caco-2) cells and Madin-Darby canine kidney (MDCK) MDCKII-MDR1 cells, with the former expressing both P-gp and BCRP, and the latter overexpressing only P-gp.^47,48^ Gratifyingly, in the Caco-2 cell permeability assay, we observed a 4-fold decrease in efflux ratio for **9** in comparison with bosutinib due to decreased efflux (basolateral to apical direction). For the MDCKII-MDR1 cell line, differences between bosutinib and **9** were much smaller in both directions, thereby indicating overall that the direct *N*-noralkoxy switch has minimal effect on P-gp substrate recognition in this series. With respect to potential toxicity by hERG ion channel inhibition, **9** showed a significant increase (*P* = 0.0086; unpaired Student’s *t* test) in IC_50_ value compared with bosutinib with IC_50_ values of 3.41 and 1.01 μM, respectively, which translated into a reduced maximal % hERG inhibition of 24 for **9** and 52 for bosutinib at 1 μM (see the Supporting Information for details). In a CYP inhibition assay across isoforms 3A4, 2D6, 2C9, and 1A2, we observed no inhibition for **9** (IC_50_ = >10 μM) except for moderate inhibition of CYP2D6 (IC_50_ = 7.6 μM), which was negative in a follow-up time-dependent inhibition (TDI) study in human liver microsomes (see the Supporting Information for details). We then conducted an Ames fluctuation assay across four *Salmonella* strains (TA98, TA100, TA1537, and TA1535) and an *in vitro* micronucleus test in Chinese hamster ovary (CHO) cells, both with and without metabolic activation by S9 (±S9), and found that **9** was neither mutagenic nor genotoxic (see the Supporting Information for details).^49,50^ Collectively, and consistent with our earlier work,^1^ we find that trisubstituted hydroxylamines are neither inherently mutagenic nor genotoxic.

**Table 2:** *In Vitro* ADMET Properties of Bosutinib and **9**^a^

parameter	bosutinib^b^	**9**
log*D*_7.4_^c^	3.1	3.5
aq sol. (μM)^d^	14	12
*f*_u, plasma_ % (H/R/M)^e^	4.5/7.2/n.d.	5.0/6.5/2.8
LMCl_int_ (H/R)^f^	288/264	228/185
*t*_1/2_ (min) (H/R)^f^	4.8/5.3	6.1/7.6
HEPCl_int_ (H/R/M)^g^	25.3/103.4/n.d.	34.3/178.2/60.1
*t*_1/2_ (min) (H/R/M)^g^	54.9/13.4	40.4/7.8/23.1
Caco-2 *P*_app_ (a-b/b-a) (10^–6^ cm/s)^h^	0.9:10.9	1.1:4.0
Caco-2 ER^h^	12.1	3.6
MDCKII-MDR1 *P*_app_ (a-b/b-a) (10^–6^ cm/s)^i^	0.4:11.3	0.5:10.3
MDCKII-MDR1 ER^i^	28.3	20.6
hERG (IC_50_) (μM)^j^	1.01	3.41
% hERG inhibition (1 μM/10 μM)^j^	52/93	24/83
CYP (IC_50_) (μM)^k^(3A4/2D6/2C9/1A2)	n.d.	24.7/7.6/13.3/>30
CYP 2D6 TDI^k^	n.d.	negative
Ames^l^	n.d.	negative
micronucleus^m^	n.d.	negative

a Assayed by Pharmaron Inc. and values presented represent the mean of *n* = 2 technical replicates, unless otherwise specified.

b Obtained from MedChemExpress (catalog no. HY-10158).

c log*D*_7.4_ determined by the shake-flask method.

d Solubility (μM) in aqueous buffer at pH 7.4, *n* = 1.

e Fraction of unbound drug in human, rat, and mouse plasma was obtained with test concentration of 5 μM by equilibrium dialysis.

f Rate of metabolism (μL/min/mg) in human and rat liver microsomes.

g Rate of metabolism (μL/min/10^6^ cells) in human, rat, and mouse hepatocytes.

h Parent compound (5 μM) was incubated for 2 h at 37 °C. ER is the efflux ratio and is calculated by *P*_app_ (b-a/a-b).

i Parent compound (1 μM) was incubated for 2 h at 37 °C. ER is the efflux ratio and is calculated by *P*_app_ (b-a/a-b).

j Obtained by manual patch-clamp system. Five doses (30, 10, 3.33, 1.11, and 0.37 μM) were run in triplicate, *n* = 3, for IC_50_ determinations.

k Low DDI is predicted with only minimal inhibition of CYP2D6 observed that was negative in a follow-up TDI study.

l Ames fluctuation test performed by Eurofins Panlabs with four common strains (TA98, TA100, TA1537, and TA1535) with and without metabolic activation (±S9) at testing concentrations of 5, 10, 50, and 100 μM. Negative result valid up to 10 μM, after which bacterial cytotoxicity was observed.

m *In vitro* micronucleus test performed by Eurofins Panlabs with and without metabolic activation (±S9) at testing concentrations of 0.2, 0.5, 2, 5, 20, 50, 200, and 500 μM. Negative result valid up to 20 μM + S9 and 5 μM – S9, after which cytotoxicity was observed. Nonstandard abbreviations: sol. solubility; DDI, drug-drug interactions; H, human; R, rat; M, mouse; n.d., not determined.

We then probed the anticancer activity of **9** in four patient-derived leukemia cell lines with bosutinib and cisplatin as the positive controls in all experiments (Figure 2). We saw strong antiproliferative activity of **9** in Ph+ CML cell lines, KU812 and MEG-01, which harbor the most common p210 BCR-ABL1 isoform, with IC_50_ values in the single-digit (5.2 nM) to low double-digit (25 nM) nanomolar range. In the Ph+ acute lymphoblastic leukemia (ALL) cell line SUP-B15, which bears a shorter p190 isoform of oncogenic BCR-ABL1, **9** showed reduced activity with an IC_50_ value of 410 nM. Finally, to confirm that the antiproliferative activity is due to specific targeting of BCR-ABL1, **9** was assessed in the leukemia cell line Molt-4, which is negative for BCR-ABL1, and the compound exhibited a markedly decreased IC_50_ value of >2 μM, as also seen with bosutinib.

**Figure 2:**
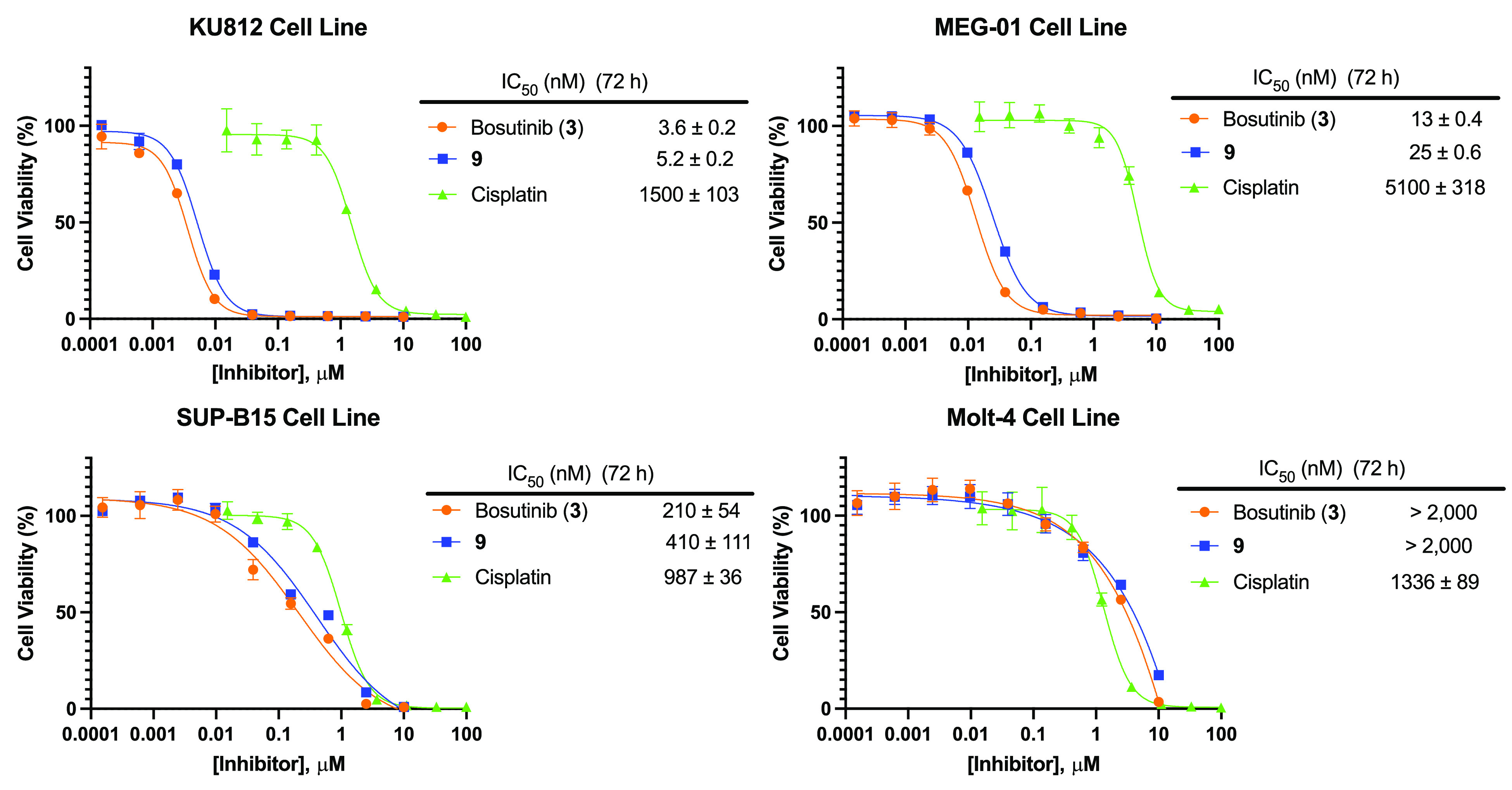
*In vitro* antiproliferative activity in patient-derived leukemia cell lines. Compound **9** displays potent antiproliferative activity in patient-derived Ph+ CML cell lines KU812 and MEG-01 (BCR-ABL1 isoform p210) and reduced activity in ALL cell line SUP-B15 (BCR-ABL1 isoform p190), while displaying minimal activity in Molt-4 (BCR-ABL1 negative). For all antiproliferative assays, points indicate the mean, and error bars indicate SD; *n* = 3 independent replicates; IC_50_ values (nM) are reported beside the dose–response curves and represent the mean ± SEM. IC_50_ (nM) values are unadjusted for FBS.

We then assessed the pharmacokinetic properties of **9** after administration into healthy CD1-mice (Table 3). No adverse events or signs of toxicity were observed for the compound at the tested doses, which is consistent with our previous study^1^ and in stark contrast to the common belief^8,9^ that hydroxylamines are inherently toxic. At 5 mg/kg intravenous (iv) and 50 mg/kg oral (po) dosing,^51^**9** exhibited high oral bioavailability (*F* = 71%) and exposure (AUC_inf_ = 11 518 h·nM; unbound AUC_u_ = 323 h·nM), an acceptable half-life (*t*_1/2_ = 2.18 h), and a high maximal plasma concentration (*C*_max_ = 1560 nM; unbound *C*_max_ = 44 nM). Overall, these experiments suggested that **9** is an orally bioavailable, potent inhibitor of BCR-ABL1 with reduced hERG affinity and reduced efflux in Caco-2 cells, all of which were achieved through an insignificant 2 amu hike in molecular weight by incorporation of a putative “structural alert.”^5,52^

**Table 3:** Pharmacokinetic Profile of **9** in CD1 Mice (*n* = 3, per Study Arm)^a^

dose	parameter	value
5 mg/kg iv	CL (mL/min/kg)^b^	52.5 ± 8.6
	*t*_*1/2*_ (hr)^c^	2.18 ± 0.24
	*V*_ss_ (L/kg)^d^	6.39 ± 1.14
50 mg/kg po	*C*_max_ (ng/mL)^e^	1560 ± 701
	*T*_max_ (hr)^f^	1.17 ± 0.76
	AUC_inf_ (h·ng/mL)^g^	11 519 ± 1756
	*t*_1/2_ (hr)^h^	2.47 ± 0.03
	*F* (%)^i^	71.1 ± 10.8

a *In vivo* pharmacokinetics (PK) was performed by Pharmaron Inc. in healthy male CD1 mice (*n* = 3, per route) via an intravenous infusion route (DMSO/10% captisol in saline = 1:99) and oral gavage route (0.5% CMC, 2.0% Tween 80, 0.06% acetic acid in H_2_O).

b Clearance obtained from intravenous infusion.

c Mean elimination half-life obtained from intravenous infusion.

d Volume of distribution at steady state.

e Peak plasma concentration.

f Time to reach peak plasma concentration.

g Area under concentration time curve from 0 to ∞.

h Mean elimination half-life obtained from oral gavage.

i Bioavailability (%) calculated with AUC_inf_ and nominal dose. All values represent the mean ± SD.

We next sought to probe the origins of improvement in drug efflux and hERG affinity imparted by the *N*-alkyl to *N*-noralkoxy switch observed with **9** by a matched-molecular pair (MMP)^53^ analysis (Figure 3). To this end, we synthesized the *N*-alkyl (**17**) and *N*-noralkoxy (**18**) derivatives of the 2-methoxyphenoxy-propyl-*N*-methylpiperazine scaffold present in bosutinib and determined log*D*_7.4_, aqueous solubility at pH 7.4 and the p*K*a values (see the Supporting Information for details). We found that both derivatives exhibited similar log*D*_7.4_ values (0.9 for **17** and 1.0 for **18**, respectively) and high solubility (>250 μM at pH 7.4). With regard to basicity, the piperazine **17** exhibited two p*K*a values (p*K*a_1_ = 4.2, p*K*a_2_ = 8.1), as is typical^54^ of 1,2-diamines with protonation of the one amine resulting in a dramatic reduction in basicity of the second because of the inductive electron-withdrawing effect of the ammonium ion. The alkoxy-substituted piperazine **18**, however, exhibited only a single p*K*a (p*K*a_1_ = 7.4), which we attribute to the terminal *N*-methylamine whose basicity is reduced from the more typical value seen in **17** by the additional electron-withdrawing effect of the δ-oxygen atom. The magnitude of this effect (Δp*K*a = −0.7) is comparable with the reduction in p*K*a of a simple tertiary amine (**19**) upon introduction of ether (**20**, Δp*K*a = −0.8) or hydroxy (**21**, Δp*K*a = −0.9) functionalities at the δ-position.^54^ The basicity of the hydroxylamine unit in **18** is reduced below the p*K*a of simple hydroxylamine (p*K*a = 5.9)^2^ by the electron-withdrawing effect of the ammonium ion arising from protonation of the terminal *N*-methylamine, thereby placing it outside the range of the measurement (pH ≈ 2–12). Thus, we propose that the observed improvement in drug efflux in Caco-2 cells and hERG affinity imparted by the *N*-alkyl to *N*-noralkoxy switch in piperazines is due to the reduction in p*K*a (both p*K*a_1_ and p*K*a_2_) wrought by the introduction of the electronegative oxygen atom.

**Figure 3:**
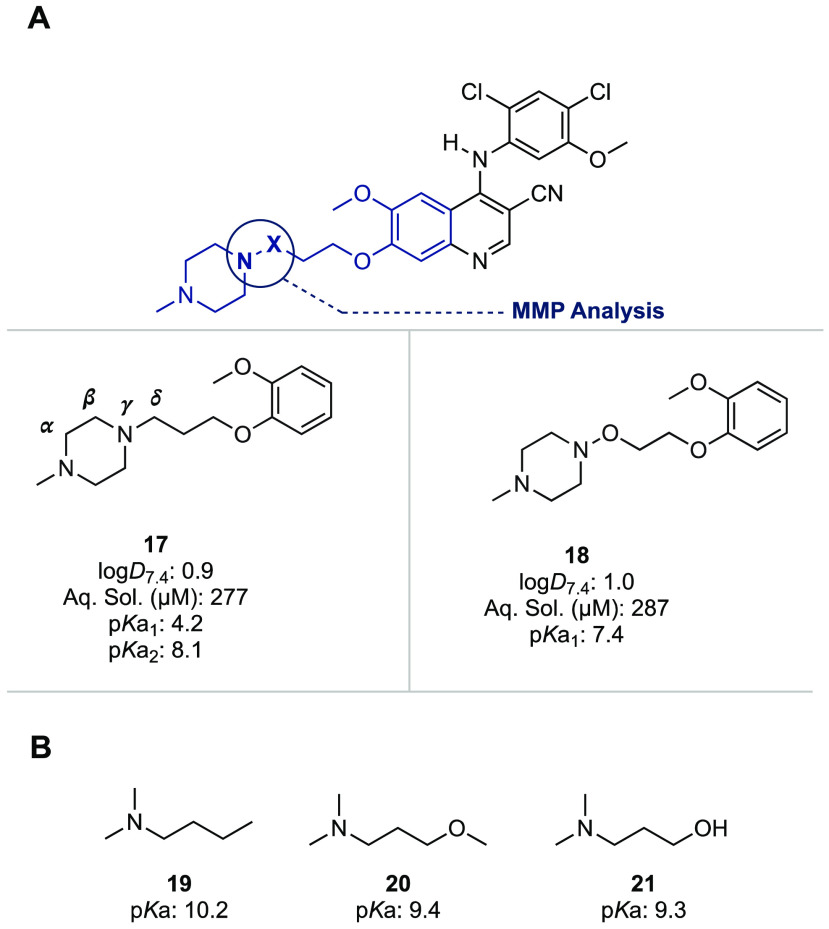
Matched molecular pair (MMP) analysis on the 2-methoxyphenoxy-propyl-*N*-methylpiperazine scaffold and p*K*a values of related δ-alkoxy substituted amines. (A) MMP analysis of a bosutinib fragment. All measurements were assayed by Pharmaron Inc., and values presented represent the mean of *n* = 2 technical replicates, unless otherwise specified. log*D*_7.4_ was determined by the shake-flask method. Solubility (μM) in aqueous buffer was measured at pH 7.4, *n* = 1. p*K*a values were determined by the pH-metric method. (B) Reported^54^ p*K*a values of related dimethylamines highlighting similarities in p*K*a values of δ-alkoxy substitution patterns.

Innovative bioisosteres that improve drug properties without significantly increasing molecular weight are highly sought after in drug discovery. In this letter, a direct bioisosteric replacement of the *N*-alkyl unit in a dual cSRC/BCR-ABL1 kinase inhibitor for an atypical amine bioisostere, the trisubstituted hydroxylamine unit, is reported. The insignificant 2 amu increase in molecular weight is achieved without the loss of biological activity and with improvement in key drug properties, such as hERG inhibition and drug efflux. Consistent with our previous work,^1^**9** critically lacks both mutagenicity and genotoxicity while maintaining oral bioavailability and achieving high free drug exposures *in vivo* with no evidence of acute toxicity. A MMP analysis suggests that the benefits conferred by the *N*-alkyl to *N*-noralkoxy switch arise from a reduction in basicity of the piperazine heterocycle. Collectively, these results lend additional support for the use of trisubstituted hydroxylamines as novel bioisosteres of *N*-alkyl units in biologically active molecules and should better position the community to apply this underrepresented functional group in the discovery of leads residing in previously uncharted chemical space.

## Supporting Information Available

Additional *in vitro* ADMET data, biological materials and methods, synthetic procedures, characterization data, Supplementary Tables S1–S28, Supplementary Figures S1–S7, NMR spectra, and references (PDF)

## Abbreviations

ABL1: Abelson murine leukemia viral oncogene homologue 1
ALL: acute lymphoblastic leukemia
Aq. Sol.: aqueous solubility
BCRP: breast cancer resistance protein
Caco-2: colon carcinoma cell line
*C*_max_: maximal concentration
cSRC: proto-oncogene tyrosine-protein kinase Src
CHO: Chinese hamster ovary
DDI: drug-drug interactions
FBS: fetal bovine serum
*f*_u_: fraction unbound
H: human
HEPCl_int_: intrinsic clearance in hepatocytes
^*i*^PrMgCl·LiCl: isopropylmagnesium chloride lithium chloride
LMCl_int_: intrinsic clearance in liver microsomes
log*D*_7.4_: logarithm of distribution at pH 7.4
M: mouse
MDCK: madine-darby canine kidney cell line
MMP: matched molecular pair
MTHP: 2-methyltetrahydropyranyl
nd: not determined
NSCLC: nonsmall-cell lung cancer
*P*_app_: apparent permeability
Ph: Philadelphia
R: rat
SD: standard deviation
SEM: standard error of the mean
Sol.: solubility
TDI: time-dependent inhibition
TKI: tyrosine kinase inhibitor
*T*_max_: time to reach maximal concentration
*V*_ss_: volume of distribution at steady state
