# Supplementary material for: Atypical N-Alkyl to N-Noralkoxy Switch in a Dual cSRC/BCR-ABL1 Kinase Inhibitor Improves Drug Efflux and hERG Affinity
Source: ACS Med Chem Lett. 2023 Dec 5;14(12):1869–75. doi: 10.1021/acsmedchemlett.3c00479 (PMC10726475; doi:10.1021/acsmedchemlett.3c00479)
Supplement: Supplementary file 1 — ml3c00479_si_001.pdf [file ml3c00479_si_001.pdf]

Supporting Information

For

Atypical *N*-Alkyl to *N*-Noralkoxy Switch in a Dual cSRC/BCR-ABL1 Kinase Inhibitor Improves Drug Efflux and hERG Affinity

Authors: Jarvis Hill[1,2], Robert M. Jones[3] and David Crich[1,2,4,*]

[1] Department of Biomedical and Pharmaceutical Sciences, University of Georgia, 250 West Green Street, Athens, GA 30602, USA.

[2] Department of Chemistry, University of Georgia, 302 East Campus Road, Athens, GA 30602, USA.

[3]P.O. Box 568, Oakley, UT 84055-0568, USA.

[4]Complex Carbohydrate Research Center, University of Georgia, 315 Riverbend Road, Athens, GA 30602, USA.

*To whom correspondence should be addressed: David.Crich@uga.edu (D.C.)

Table of Contents

1. Biological Methods and Data S2
2. Biological Materials and Data S3
3. General Procedures for Chemical Synthesis, Materials and Instrumentation S23
4. Synthesis Procedures S24
5. Catalog of Spectra S32
6. References S63

1. Biological Methods and Data

Cell lines

The MEG-01, SUP-B15, KU812 and Caco-2 cell lines were obtained from ATCC. The Molt-4 cell line was obtained from SIBS. MCKII-MDR1 cells were obtained from the Netherlands Cancer Institute. HEK293 cells were obtained from Invitrogen. MEG-01, Molt-4, and KU812 cells were cultured in RPMI1640 (Invitrogen) +10%FBS. SUP-B15 cells were cultured in IMDM (Hyclone) +20% FBS + 0.05 mM β-ME. HEK293 cells were cultured in DMEM (Gibco) with 10% FBS, 0.1 mM NEAA, 25 mM HEPES, 100 U/mL penicillin-streptomycin, 5 μg/mL blasticidin and 400 μg/mL geneticin. All cells were cultured in a humidified incubator with 5% CO_2 at 37 °C.

Cell viability assay

Viability assays using MEG-01, SUP-B15, KU812 and Molt-4 cells were performed at Crown BioScience. Cells were plated into 96-well plates at 6,000-8,000 cells per well and dosed in triplicate (n = 3) in a nine-point, fourfold dilution series with compounds (0.15 nM to 10 μM) in DMSO and incubated for 72 h. After 72 h, cell viability was assayed by CellTiter-Glo Luminescent Viability Assay (Promega). Dose-response curves were generated and used to calculate the IC_{50} values which were calculated on GraphPad Prism from the non-linear regression equation fitted with a sigmoidal dose response and are presented as the mean ± SEM.

***In vitro* ADMET**

Lipophilicity, solubility, plasma protein binding, metabolic stability in liver microsomes and hepatocytes, permeability studies, hERG channel inhibition and CYP inhibition profile was determined by Pharmaron Inc. using methods previously derscribed.[1]

Mutagenicity and Genotoxicity Tests

Mutagenicity and genotoxicity was assessed by Eurofins Panlabs using the AMES fluctuation assay and *in vitro* micronucleus test (MNT) according to methods previously described.[1]

p*K*a Determination

Compounds **17** and **18** underwent p*K*a determination at Pharmaron Inc., using the pH-metric method. Titration data was obtained from a titration of both compounds and the p*K*a test results obtained by Yasuda-Shedlovsky extrapolation for the pH-metric p*K*a method. The range for p*K*a determination was pH = 2-12.

Animal studies

Animal experiments were performed at Pharmaron Inc. and animal use was approved by Pharmaron's Institutional Animal Care and Use Committee (IACUC) in Pharmaron (Pharmaron IACUC, Protocol #PK-M-07182022) following the guidance of AAALAC. Six- to eight-week-old male CD1 mice (approximately 20-30 g) obtained from Si Bei Fu Laboratory Animal Technology Co., were used in the pharmacokinetic studies. Animals were housed at 20-25 °C with humidity ranging from 40-70% relative humidity, were exposed to 12 h light and dark cycles, and were supplied with food and water ab libitum.

***In vivo* studies**

Pharmacokinetic studies of 9 in CD1 mice

Standard pharmacokinetic assessment of **9** was done by IV (intravenous) tail vein injection (formulation: DMSO: 10% captisol in saline = 1:99) and PO (oral) oral gavage (formulation: 0.5% CMC, 2.0% Tween 80, 0.06% Acetic acid in water) followed by blood sampling at 8 time points for IV (0.0833, 0.25, 0.5, 1,2, 4, 7, 24 h post dose) and 7 time points for PO (0.25, 0.5, 1, 2, 4, 8, 24 h post dose) with n = 3 animals per

dosing route (n = 6 total). Approximately 0.03 mL of blood was collected from the dorsal metatarsal vein at each time point. Blood at each sampling point was transferred into a plastic micro centrifuge tube containing K_2-EDTA and collection tubes with blood samples and anticoagulant were inverted several times for proper mixing of the tube contents and placed on ice prior to centrifugation for plasma. Blood samples were centrifuged at 4 °C, 4,000 g for 5 min to obtain plasma. Samples were stored in a freezer at – 75 °C prior to analysis. Concentrations of test articles in the plasma samples were determined using LC-MS/MS and WinNonlin 8.3 (Phoenix^{TM}) was used for pharmacokinetic calculations. The values obtained were plotted on GraphPad Prism software and are presented as the mean ± SD.

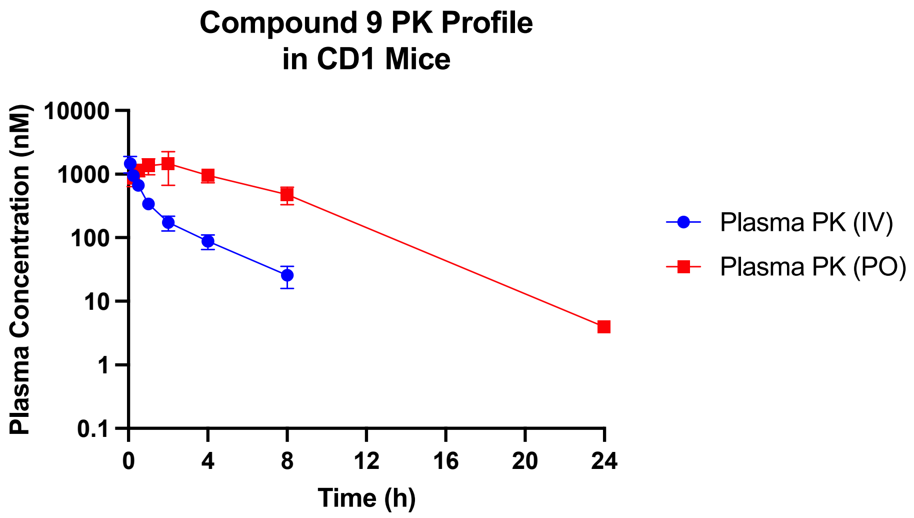

Figure S1. Total plasma vs time profile (0 to 24 h) of **9** after administration into CD1 mice at a single dose of 5 mg/kg IV and 50 mg/kg PO. For pharmacokinetic profiles, points indicate the mean and error bars indicate SD; n = 3 animals per route (n = 6 total). For tabled values, refer to Table 3 in the manuscript.

Statistical analyses

Statistical analysis was performed using GraphPad Prism 9.0. Data are presented as mean ± SD or SEM as indicated when $n = \geq 3$, or as the geometric mean when n = 2. For *in vitro* ADME and kinase activity studies, data is presented as mean of n = 2 independent replicates. For *in vivo* PK experiments, data is presented as the mean ± SD, n = 3 animals per study arm. For *in vitro* short-term growth delay experiments, IC_{50} values were determined from the nonlinear regression equation fitted with a sigmoidal dose response curve and are presented as the mean ± SD, n = 3 independent replicates and IC_{50} values are reported beside the dose-response curve and represent the mean ± SEM. hERG IC_{50} values between bosutinib and **9** were compared using an unpaired Student's T-test. A P value of < 0.05 was considered statistically significant.

2. Biological Materials and Data

Biochemical human RTK kinase enzymatic radiometric assay, KinaseProfiler

Table S1 | Biochemical IC_{50} results for compounds against BCR-ABL1 and cSRC kinases.[a]

**Compound**	**Bosutinib**	**9**
**BCR-ABL1 IC_{50} (nM)**		
wt	<1	< 1
H396P	< 1	< 1
M351T	< 1	< 1
Q252H	< 1	< 1
T315I	44	47
Y253F	< 1	< 1
**cSRC IC_{50} (nM)**[a]		
wt	2	2

[a]Values < 1 nM were below the sensitivity of the test and as such are reported as < 1 nM.

Plasma protein binding by equilibrium dialysis

Table S2 | Plasma details used in the plasma protein binding assay.

Item	Supplier
Human plasma (mixed gender)	Ltd-People’s Hospital of Shandong Weifang Hi-Tech Industrial Development Zone (Batch No. P-KP(382-431)-20210827)
Rat plasma (SD/mixed gender)	BiolVT (Batch No. RAT501985)
Mouse plasma (CD-1/mixed gender)	IPHASE or BiolVT (Batch No. MSE427337)

Table S3 | Results for plasma protein binding in human, rat and mouse plasma.

Compound	Species	%Bound	%Unbound	%Recovery	%Remaining at 6 h
Ketoconazole	Human	99.18	0.82	98.56	101.83
Bosutinib	Human	95.47	4.53	94.01	102.03
**9**	Human	95.05	4.95	92.41	101.74
Ketoconazole	Rat	99.33	0.67	102.55	104.21
Bosutinib	Rat	92.80	7.20	91.06	90.71
**9**	Rat	93.55	6.45	96.84	102.38
Ketoconazole	Mouse	99.42	0.58	96.82	96.27
**9**	Mouse	97.17	2.83	99.45	106.02

Metabolic stability in human and rat liver microsomes

Table S4 | Microsome details used in the microsome materials assay.

Item	Supplier
Human Liver Microsomes, Pooled, Mixed Gender	BD Gentest (Cat No. 452117; Lot No. 38297)
Rat Liver Microsomes, Pooled, Male SD	BD Gentest (Cat No. 452501, Lot No. 1300002)

Table S5 | Results for metabolic stability in human and rat liver microsomes expressed as % remaining.

Compound	Species	Assay Format	Remaining Percentage (%)				
			0.5 min	15 min	30 min	45 min	60 min
Verapamil	Human	+Cofactors	100.00	10.95	BLOD	BLOD	BLOD
		-Cofactors	100.00	-	-	-	102.68
Bosutinib	Human	+Cofactors	100.00	12.40	2.94	1.16	BLOD
		-Cofactors	100.00	-	-	-	91.23
**9**	Human	+Cofactors	100.00	19.17	BLOD	BLOD	BLOD
		-Cofactors	100.00	-	-	-	95.07
Verapamil	Rat	+Cofactors	100.00	4.12	3.75	4.53	3.66
		-Cofactors	100.00	-	-	-	98.27
Bosutinib	Rat	+Cofactors	100.00	14.79	5.02	2.62	1.20
		-Cofactors	100.00	-	-	-	108.70
**9**	Rat	+Cofactors	100.00	26.34	BLOD	BLOD	BLOD
		-Cofactors	100.00	-	-	-	95.46

Abbreviations: BLOD, below level of detection.

Metabolic stability in hepatocytes

Table S6 | Hepatocyte details used in the hepatocyte stability assay.

**Item**	**Supplier**
Human Hepatocytes, Mixed-Gender	BiolVT (Cat No. X008001, Lot No. QZW)
SD Rat Hepatocytes, Male	BiolVT (Cat. No M00005, Lot No. DVB)
CD1 Mouse Hepatocytes, Male	TPCS (Cat No. CMH-100CD-SQ, Lot No. CMH100CD-V01299)
Beagle Dog Hepatocytes, Male	BiolVT (Cat. No. M00205, Lot No. UHC)

Table S7 | Results for hepatocyte stability data in human, rat, mouse and dog hepatocytes.

**Compound**	**Species**	**Remaining Percentages (%)**					
		**0.5 min**	**15 min**	**30 min**	**60 min**	**90 min**	**120 min**
Verapamil	Human	100.00	48.08	31.70	12.32	BLOD	BLOD
Bosutinib	Human	100.00	73.99	79.28	53.95	34.15	20.73
**9**	Human	100.00	90.07	69.58	44.09	23.58	13.59
Verapamil	Rat	100.00	7.10	BLOD	BLOD	BLOD	BLOD
Bosutinib	Rat	100.00	50.17	21.78	5.83	1.83	0.73
**9**	Rat	100.00	27.50	5.80	BLOD	BLOD	BLOD
Verapamil	Mouse	100.00	6.39	BLOD	BLOD	BLOD	BLOD
**9**	Mouse	100.00	52.02	34.17	15.85	9.79	5.69
Verapamil	Dog	100.00	54.45	31.71	15.55	7.46	4.09
**9**	Dog	100.00	86.51	73.17	39.27	19.26	9.31

Abbreviations: BLOD, below level of detection.

Caco-2 permeability

Table S8 | Caco-2 cellular details used in the Caco-2 cellular permeability assay.

**Item**	**Supplier**
Caco-2 cells	ATCC (ATCC No. HTB-37)
HTS Transwell 96 Well	Corning (Cat No. 3391)

Table S9 | Results for Caco-2 cellular permeability.

**Compound**	**$P_{app\text{(a-b)}}$ (10^{-6}, cm/s)**	**$P_{app\text{(b-a)}}$ (10^{-6}, cm/s)**	**Efflux Ratio**	**Recovery% (AP-BL)**	**Recovery% (BL-AP)**
Metoprolol	23.08	21.82	0.95	105.54	100.41
Digoxin	0.34	15.58	45.63	102.53	101.40
Bosutinib	0.86	10.87	12.70	52.12	70.73
**9**	1.05	4.04	3.88	39.68	54.25

Abbreviations: P_{app}, apparent permeability; AP, apical; BL, basolateral.

MDCKII-MDR1 Permeability

Table S10 | MDCKII-MDR1 cellular details used in the MDCKII-MDR1 permeability assay.

Item	Supplier
MDCKII-MDR1 cells	Netherlands Cancer Institute (Amsterdam)
HTS Transwell 96 Well	Corning (Cat No. 3391)

Table S11 | Results for MDCKII-MDR1 cellular permeability.

Compound	$P_{app(a\text{-}b)}$ (10^{-6}, cm/s)	$P_{app(b\text{-}a)}$ (10^{-6}, cm/s)	Efflux Ratio	Recovery% (AP-BL)	Recovery% (BL-AP)
Metoprolol	27.89	30.96	1.11	99.89	96.11
Digoxin	0.26	10.20	38.63	96.32	92.45
Bosutinib	0.38	11.34	29.85	56.02	65.52
**9**	0.52	10.28	19.78	45.83	58.85

Abbreviations: P_{app}, apparent permeability; AP, apical; BL, basolateral.

hERG Safety evaluation by manual patch-clamp system

Table S12 | HEK293 cellular details used in hERG safety evaluation.

Item	Supplier
HEK 293 Cell Line	Invitrogen (Cat No. K1236)
TrypLE™ Express	Gibco (Cat No. 12604)
Dofetilide	TRC (Cat No. D525700)

Table S13 | hERG safety evaluation results.

Compound	hERG IC_{50} (μM)[a]
Dofetilide[b]	0.015 ± 0.0008
Bosutinib	1.01 ± 0.4
**9**	3.41 ± 0.3

[a]IC_{50} values are presented as the mean ± SEM. [b]Dofetilide tested at 5 concentrations (0.00185, 0.00556, 0.01667, 0.05000, 0.15000 μM) and run in triplicate.

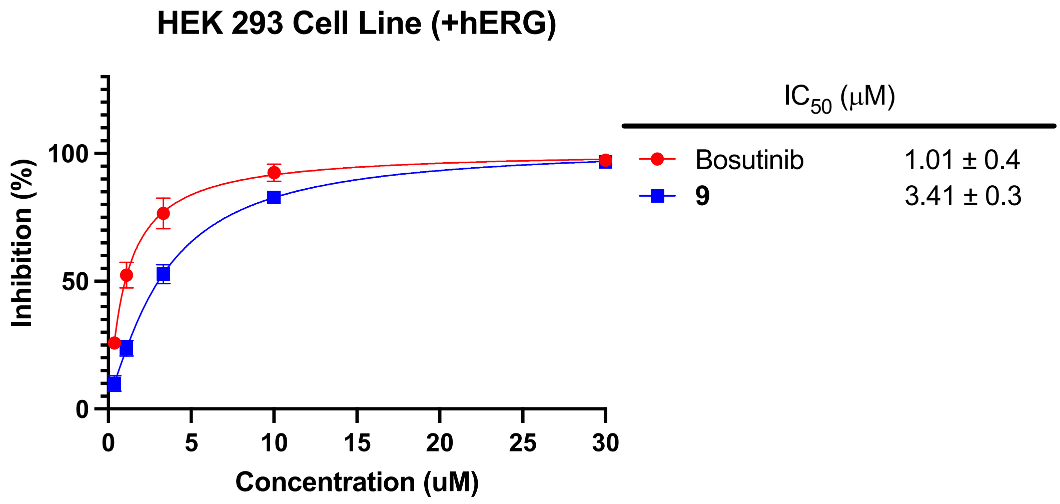

Figure S2. Compound **9** has low hERG inhibitory potential; points indicate mean, and error bars indicate the SD; n = 3 independent replicates; IC_{50} values (μM) are reported beside the dose-response curves and represent mean ± SEM.

CYP Inhibition of CYP1A2, CYP2C9, CYP2D6 and CYP3A4 in human liver microsomes

Table S14 | CYP450 control compound details used in the CYP inhibition assay.

CYP Isoform	Positive Inhibitors	Final Concentration (µM)
CYP1A2	Furafylline	0, 0.0075, 0.025, 0.075, 0.25, 0.75, 2.5, 7.5, 25
CYP2C9	Sulfaphenazole	0, 0.0015, 0.005, 0.015, 0.05, 0.15, 0.5, 1.5, 5
CYP2D6	Quinidine	0, 0.0015, 0.005, 0.015, 0.05, 0.15, 0.5, 1.5, 5
CYP3A4	Ketoconazole	0, 0.0015, 0.005, 0.015, 0.05, 0.15, 0.5, 1.5, 5

Table S15 | CYP450 substrate concentration details.

CYP Isoform	Substrate	Working Concentration (µM)	Final Concentration (µM)	Incubation Time
CYP1A2	Phenacetin	800	40	20 min
CYP2C9	Diclofenac	120	6	5 min
CYP2D6	Dextromethorphan	40	2	20 min
CYP3A4	Midazolam	20	1	5 min

Table S16 | Results for CYP450 inhibition assay.

Compound	IC_{50} (µM)[a]			
	CYP1A2	CYP2C9	CYP2D6	CYP3A4-M
Furafylline	2.08	-	-	-
Sulfaphenazole	-	0.25	-	-
Quinidine	-	-	0.032	-
Ketoconazole	-	-	-	0.019
**9**	>30	13.3	7.60	24.7

[a]IC_{50} values are presented as the mean (*n* = 2 independent replicates).

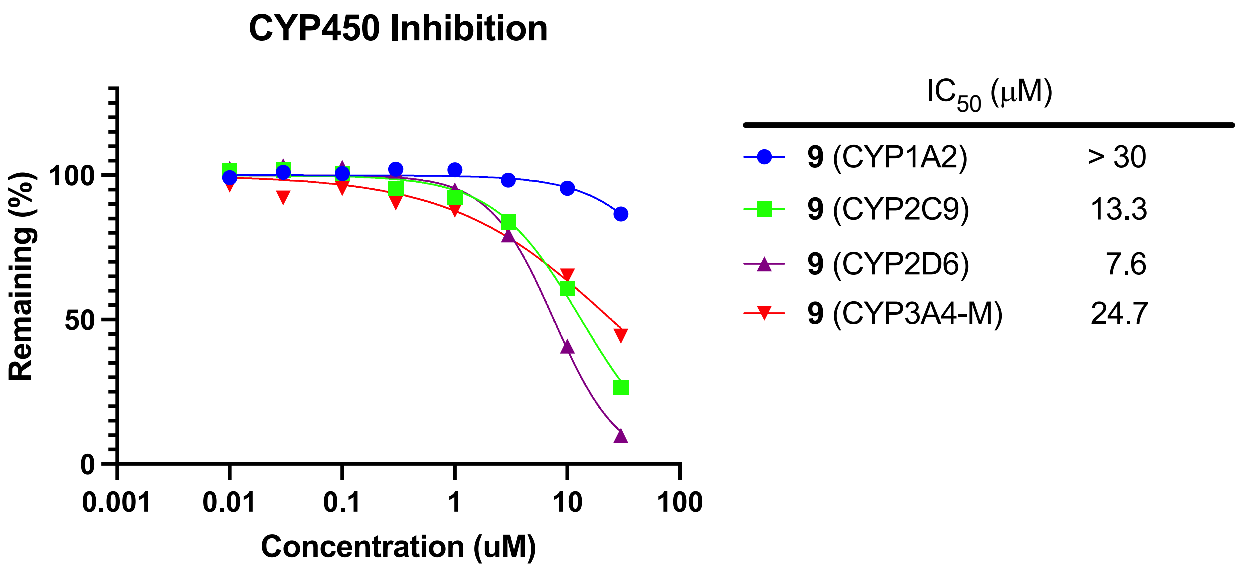

Figure S3. Low DDI is predicted for compound **9**. Only moderate CYP2D6 inhibition was observed. Points indicate mean; $n = 2$ independent replicates. IC_{50} (μM) are reported beside the dose-response curves and represent the mean.

Direct and time-dependent inhibition of CYP2D6 in human liver microsomes

Table S17 | Microsome details used in direct and time-dependent CYP2D6 inhibition study.

Item	Supplier
Pooled Human Liver Microsomes, Mixed Gender	BD Gentest (Cat. No. 452117, Lot. No. 38297)

Table S18 | CYP2D6 control compound concentration details.

CYP Isoform	Positive Control	Working Concentration (µM)	Final Concentration (µM)
CYP2D6	Paroxetine	0.006, 0.02, 0.06, 0.2, 0.6, 2	0.03, 0.1, 0.3, 1, 3, 10

Table S19 | Final CYP2D6 substrate concentrations used in assay.

CYP Isoform	Substrate	Working Concentration (µM)	Final Concentration (µM)	Incubation Time
CYP2D6	Bufuralol	40	2	20 min

Table S20 | Results for CYP2D6 time-dependent inhibition assay in human liver microsomes.

Compound	Pre-incubation	CYP2D6	
		IC_{50} (µM)[a]	Inhibition percentage(%) at Top conc.
Paroxetine	0 min	0.21	91.68
	30 min without NADPH	0.20	92.23
	30 min with NADPH	0.044	96.01
**9**	0 min	6.99	81.66
	30 min without NADPH	6.58	81.09
	30 min with NADPH	6.51	80.12

[a]IC_{50} values are presented as the mean (n = 2 independent replicates).

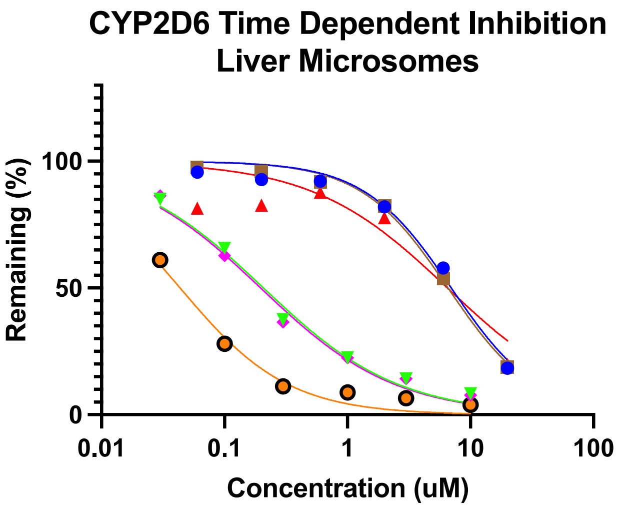

	IC_{50} (μM)
**9** (0 min)	6.99
**9** (30 min without NADPH)	6.58
**9** (30 min with NADPH)	6.51
Paroxetine (0 min)	0.21
Paroxetine (30 min without NADPH)	0.20
Paroxetine (30 min with NADPH)	0.044

Figure S4. Compound **9** was negative in a follow-up CYP2D6 time-dependent inhibition (TDI) study. Points indicate mean; $n = 2$ independent replicates. IC_{50} (μM) are reported beside the dose-response curves and represent the mean.

AMES fluctuation test

Table S21 | *Salmonella* strains used in the AMES fluctuation assay.

Salmonella Strain	DNA Target	His Mutation	Reversion Event
TA98	CGCGCGCG	hisD3052	Frameshifts
TA100	GGG	hisG46	Base-pair substitution
TA1535	GGG	hisG46	Base-pair substitution
TA1537	Near CCC run	hisC3076	Frameshifts

Table S22 | Results for AMES fluctuation assay of **9**.[a]

Compound and Test	Test Concentration	Count (# of wells)	Positive Significance (- to +++)	Fisher Exact Test (p-value)
**AMES Test (TA98-S9)**				
2-Aminoanthracene	1.0E-06 M	1	-	1.0000
2-Nitrofluorene	3.0E-05 M	30	+++	0.0000
9-Aminoacridine	1.0E-05 M	2	-	0.5000
Streptozotocin	2.5E-06 M	1	-	1.0000
**9**	5.0E-06 M	2	-	0.5000
**9**	1.0E-05 M	1	-	1.0000
**9**	5.0E-05 M	0	-	0.5000
**9**	1.0E-04 M	0	-	0.5000
**AMES Test (TA98+S9)**				
2-Aminoanthracene	2.0E-06 M	48	+++	0.0000
2-Nitrofluorene	3.0E-05 M	28	+++	0.0000
9-Aminoacridine	1.0E-05 M	1	-	0.5000
Streptozotocin	2.5E-06 M	6	+	0.0132
**9**	5.0E-06 M	1	-	0.5000
**9**	1.0E-05 M	1	-	0.5000
**9**	5.0E-05 M	1	-	0.5000
**9**	1.0E-04 M	0	-	1.0000

**AMES Test (TA100-S9)**				
2-Aminoanthracene	1.0E-06 M	2	-	0.5000
2-Nitrofluorene	3.0E-05 M	2	-	0.5000
9-Aminoacridine	1.0E-05 M	0	-	0.5000
Streptozotocin	2.5E-06 M	39	+++	0.0000
**9**	5.0E-06 M	4	-	0.1808
**9**	1.0E-05 M	2	-	0.5000
**9**	5.0E-05 M	0	-	0.5000
**9**	1.0E-04 M	0	-	0.5000
**AMES Test (TA100+S9)**				
2-Aminoanthracene	1.0E-06 M	17	++	0.0012
2-Nitrofluorene	3.0E-05 M	0	-	0.0586
9-Aminoacridine	1.0E-05 M	2	-	0.3387
Streptozotocin	2.5E-06 M	19	+++	0.0003
**9**	5.0E-06 M	3	-	0.5000
**9**	1.0E-05 M	3	-	0.5000
**9**	5.0E-05 M	0	-	0.0586
**9**	1.0E-04 M	0	-	0.0586
**AMES Test (TA1535-S9)**				
2-Aminoanthracene	1.0E-06 M	0	-	1.0000
2-Nitrofluorene	3.0E-05 M	0	-	1.0000
9-Aminoacridine	1.0E-05 M	0	-	1.0000
Streptozotocin	2.5E-06 M	46	+++	0.0000
**9**	5.0E-06 M	0	-	1.0000
**9**	1.0E-05 M	0	-	1.0000
**9**	5.0E-05 M	0	-	1.0000
**9**	1.0E-04 M	0	-	1.0000
**AMES Test (TA1535+S9)**				

2-Aminoanthracene	1.0E-06 M	12	+++	0.0009
2-Nitrofluorene	3.0E-05 M	0	-	0.5000
9-Aminoacridine	1.0E-05 M	1	-	1.0000
Streptozotocin	2.5E-06 M	48	+++	0.0000
**9**	5.0E-06 M	6	-	0.0556
**9**	1.0E-05 M	1	-	1.0000
**9**	5.0E-05 M	0	-	0.5000
**9**	1.0E-04 M	0	-	0.5000
**AMES Test (TA1537-S9)**				
2-Aminoanthracene	1.0E-06 M	0	-	1.0000
2-Nitrofluorene	3.0E-05 M	6	+	0.0132
9-Aminoacridine	1.0E-05 M	25	+++	0.0000
Streptozotocin	2.5E-06 M	1	-	0.5000
**9**	5.0E-06 M	0	-	1.0000
**9**	1.0E-05 M	0	-	1.0000
**9**	5.0E-05 M	0	-	1.0000
**9**	1.0E-04 M	0	-	1.0000
**AMES Test (TA1537+S9)**				
2-Aminoanthracene	2.0E-06 M	11	++	0.0018
2-Nitrofluorene	3.0E-05 M	2	-	0.5000
9-Aminoacridine	1.0E-05 M	31	+++	0.0000
Streptozotocin	2.5E-06 M	0	-	0.5000
**9**	5.0E-06 M	0	-	0.5000
**9**	1.0E-05 M	0	-	0.5000
**9**	5.0E-05 M	0	-	0.5000
**9**	1.0E-04 M	0	-	0.5000

[a]Significance of the positive counts between treatment and control were calculated using a one-tailed Fisher's exact test. Significance levels are reported as follows: weak positive, if $P < 0.05$ (denoted as "+"); strong positive, if $P < 0.01$ (denoted as "++"); very strong positive, if $P < 0.001$ (denoted as "++"). Hyphens (-) indicate negative results.

Table S23 | Results for background bacterial cytotoxicity assay of **9**.

Compound and Test	Test Concentration	Mean % Control (n = 3)	Cytotoxicity (% of control)[a]	Flag[b]
**Bacterial Cytotoxicity (TA98-S9)**				
**9**	6.0E-07 M	102.9	103	-
**9**	1.2E-06 M	91.6	92	-
**9**	2.5E-06 M	92.9	93	-
**9**	5.0E-06 M	90.6	91	-
**9**	1.0E-05 M	90.6	91	-
**9**	2.5E-05 M	81.9	82	-
**9**	5.0E-05 M	36.6	37	Cytox.
**9**	1.0E-04 M	2.9	3	Cytox.
**Bacterial Cytotoxicity (TA100-S9)**				
**9**	6.0E-07 M	96.1	96	-
**9**	1.2E-06 M	88.1	88	-
**9**	2.5E-06 M	92.1	92	-
**9**	5.0E-06 M	93.3	93	-
**9**	1.0E-05 M	105.8	106	-
**9**	2.5E-05 M	89.3	89	-
**9**	5.0E-05 M	1.8	2	Cytox.
**9**	1.0E-04 M	2.6	3	Cytox.
**Bacterial Cytotoxicity (TA1535-S9)**				
**9**	6.0E-07 M	105.0	105	-
**9**	1.2E-06 M	103.5	104	-
**9**	2.5E-06 M	103.9	104	-
**9**	5.0E-06 M	107.3	107	-
**9**	1.0E-05 M	102.0	102	-
**9**	2.5E-05 M	81.3	81	-
**9**	5.0E-05 M	2.9	3	Cytox.

**9**	1.0E-04 M	2.9	3	Cytox.
**Bacterial Cytotoxicity (TA1537-S9)**				
**9**	6.0E-07 M	103.4	103	-
**9**	1.2E-06 M	102.1	102	-
**9**	2.5E-06 M	103.1	103	-
**9**	5.0E-06 M	102.1	102	-
**9**	1.0E-05 M	98.6	99	-
**9**	2.5E-05 M	68.5	68	-
**9**	5.0E-05 M	2.1	2	Cytox.
**9**	1.0E-04 M	2.1	2	Cytox.

[a]Cytotoxicity is presented as % of control growth. [b]A cytotoxicity value of less than 60% is flagged and the compound is considered as toxic (denoted as Cytox.) at the respective concentration. A hyphen (-) denotes no cytotoxicity at the respective concentration.

Micronucleus test

Table S24 | Results for *in vitro* micronucleus test of **9**.

Compound and Test	Test Concentration	Scored Cells	%Cytotoxicity CBPI Index	%Cytotoxicity Cell Numbers	%Micronucleated Cells	*P*-value[a]	Result	Flag
**Micronucleus (CHO+S9, HCA)**								
Control + S9	0 M	4323	-8.5	39.6	0.62	N/A	N/A	-
Cyclophosphamide	3.6 E-05 M	2050	-2.4	59.0	3.07	0.0001	+	-
**9**	2.0E-07 M	2399	-22.5	45.8	0.21	0.0462	-	-
**9**	5.0E-07 M	2103	-17.8	46.4	0.38	0.1677	-	-
**9**	2.0E-06 M	2093	-16.8	43.9	0.47	0.2854	-	-
**9**	5.0E-06 M	2174	-16.8	34.3	0.57	0.4342	-	-
**9**	2.0E-05 M	2208	-15.4	52.1	0.36	0.1254	-	-
**9**	5.0E-05 M	N/A	N/A	N/A	N/A	N/A	N/A	Cytox.
**9**	2.0E-04 M	N/A	N/A	N/A	N/A	N/A	N/A	Cytox.
**9**	5.0E-04 M	N/A	N/A	N/A	N/A	N/A	N/A	Cytox.
**Micronucleus (CHO-S9, HCA)**								
Control - S9	0 M	4316	0.0	0.0	0.33	N/A	N/A	-
Mitomycin C	3.0E-07 M	2023	8.8	20.7	1.04	0.0180	+	-
**9**	2.0E-07 M	2412	3.9	-16.6	0.38	0.3857	-	-
**9**	5.0E-07 M	2141	3.7	-13.8	0.32	0.4918	-	-
**9**	2.0E-06 M	2205	-1.1	-12.0	0.26	0.3343	-	-

**9**	5.0E-06 M	2175	6.1	-9.6	0.22	0.2772	-	-
**9**	2.0E-05 M	N/A	N/A	N/A	N/A	N/A	N/A	Cytox.
**9**	5.0E-05 M	N/A	N/A	N/A	N/A	N/A	N/A	Cytox.
**9**	2.0E-04 M	N/A	N/A	N/A	N/A	N/A	N/A	Cytox.
**9**	5.0E-04 M	N/A	N/A	N/A	N/A	N/A	N/A	Cytox.

[a]Significance of the positive counts between treatment and control were calculated using a one-tailed t-test with two sample equal variance. Significance levels are reported as follows: "+" if $P < 0.05$ by t-test and % of micronucleated cells at least 3-fold higher than background levels. "+/-" if $P < 0.05$ by t-test and % of micronucleated cells at least 2-fold higher than background levels. "-" if $P > 0.05$ by t-test and % of micronucleated cells less than 2-fold higher than background levels. Hyphens (-) indicate negative results. Cytotoxicity (denoted as Cytox.) refers to high cytotoxicity resulting in an insufficient number of scorable cells (> 80% cytotoxicity).

In vitro CTG assay

Table S25 | Materials used in cell viability assays.

Item	Supplier
RPMI 1640	Invitrogen (Cat No. C22400500BT)
IMDM	Hyclone (Cat No. SH3028.01)
FBS	ExCell Bio (Cat No. FND500)
96-Well Polystyrene Microplates	Corning (Cat No. 3610)
CellTiter-Glo Luminescent Cell Viability Assay	Promega (Cat No. G7572)
Backseal Black Adhesive Bottom Seal	Perkin Elmer (Cat No. 6005189)
Cisplatin	Qilu Pharma (Batch No. 6J015A89)

Table S26 | Cell line information for cell viability assays.

Cell Line Name	Tissue Origin	Culture Property	Cell Culture Medium[a]	Compound Treatment Time	Seeding Density
MEG-01	Blood	Adherent & Suspension	RPMI1640+10%FBS	72 h	8000
Molt-4	Blood	Suspension	RPMI1640+10%FBS	72 h	8000
SUP-B15	Bone Marrow	Suspension	IMDM+20%FBS+0.05mM β-ME	72 h	6000
KU812	Peripheral Blood	Suspension	RPMI1640+10%FBS	72 h	8000

[a]Cells were cultured in a 37 °C incubator with 5% CO_2.

3. General Procedures for Chemical Synthesis, Materials and Instrumentation

All reactions were conducted in oven dried glassware capped with a rubber septa under an argon atmosphere unless otherwise stated. All organic solutions were concentrated under reduced pressure on a rotary evaporator and water bath. Flash-column chromatography was performed using silica gel (Fischer Silica Gel Sorbent (230-400 Mesh, Grade 60)). Thin-layer chromatography (TLC) was carried out with 250 µM glass back silica (XHL) plates with fluorescent indicator (254 nm). TLC plates were visualized by exposure to ultraviolet light (UV) and/or submersion in ceric ammonium molybdate (CAM) in ethanol followed by heating on a hot plate (120 °C, 10-15 s).

Solvents were purchased from Sigma-Aldrich and used without further purification. *tert*-butyldimethyl(2-((2-methyltetrahydro-2*H*-pyran-2-yl)peroxy)ethoxy)silane[1,2] (**11**) were prepared according to literature procedures and spectral data were in accord with that previously reported in the literature. *N*-Methylpiperazine was purchased from Sigma-Aldrich. Isopropylmagnesium chloride lithium chloride complex solution (1.3 M in THF) was purchased from Sigma-Aldrich. Tetrabutylammonium fluoride (1 M in THF) was purchased from TCI. Thionyl chloride was purchased from Alfa Aesar. Sodium Hydride (60% dispersion in mineral oil) was purchased from Sigma-Aldrich. Pyridine-hydrochloride was purchased from Sigma-Aldrich. 4-Chloro-7-hydroxy-6-methoxyquinoline-3-carbonitrile was purchased from Ambeed. 2,4-Dichloro-5-methoxyaniline was purchased from AK Scientific. Bosutinib (**3**) was purchased from MedChemExpress (Cat No. HY-10158). The purity of the assayed compound (**9**) was determined to be > 95% by UHPLC (Page S62) prior to study initiation. The purity of commercial bosutinib (**3**) was confirmed by nuclear magnetic resonance (NMR) and high-resolution mass spectrometry (HRMS) prior to *in vitro* study initiation.

NMR spectra of all compounds were obtained in either $CDCl_3$ (δ_H 7.26 and δ_C 77.16 ppm, respectively) or Toluene-D_8 (δ_H 7.09, 6.98, 7.00, 2.09 and δ_C 137.86, 129.24, 128.33, 125.49, 20.40 ppm, respectively) using a 500 MHz, EZC500 JEOL instrument at 298 K unless otherwise specified. The chemical shifts (δ) are calculated with respect to residual solvent peak and are given in ppm. Multiplicities are abbreviated as follows: s (singlet), m (multiplet), b (broad), d (doublet), t (triplet), q (quartet), hept (heptet). HRMS were obtained on a ThermoFisher Orbitrap Q-Exactive using electrospray ionization (ESI). Ultra high performance liquid chromatography (UHPLC) trace of compound **9** was obtained using a ThermoFisher Vanguish UHPLC with PDA detector and an Acclaim 120 ^{18}C 4.6 x 50 mm column and the %purity determined using the Avalon peak area algorithm. Melting point of solid **15** was determined on a Barstead Electrothermal 9100.

Safety note: While we have encountered no decomposition, standard precaution should be exercised when working with peroxides (avoid light, reducing agents, exposure to excessive heat and work behind a glass shield). In the preparation of **12**, magnesium amide generation evolves propane (g). Caution should be exercised on larger scale. See Ref. 1 for a detailed experimental set-up.

4. Synthetic Procedures

Synthesis of 1-(2-((*tert*-butyldimethylsilyl)oxy)ethoxy)-4-methylpiperazine (**12**) *via* Literature Procedure[1]

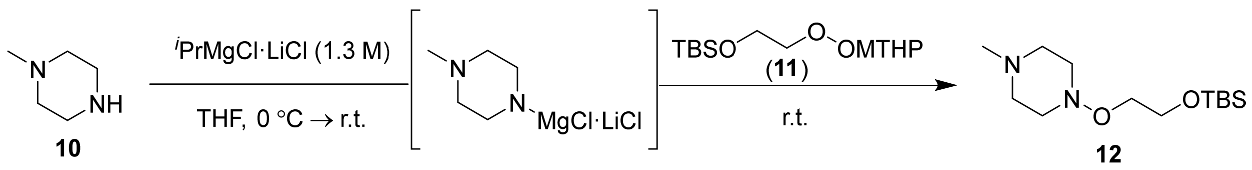

N-methylpiperazine (**10**) (6.31 mL, 56.9 mmol, 3.0 eq.) was dissolved in anhydrous THF (47.5 mL) at 0 °C and ^{i}PrMgCl·LiCl (1.3M in THF) (36.4 mL, 47.4 mmol, 2.5 eq.) was added slowly. The solution was warmed to r.t. and stirred for 45 min.* After which, **11**[1-5] (5.5 g, 18.95 mmol, 1.0 eq.) was added dropwise as a solution in anhydrous THF (47.5 mL) and the mixture stirred for 3 h. The solution was then quenched with $NaHCO_3$ (100 mL) and the layers separated. The aqueous layer was extracted with EtOAc (3x, 75 mL) and the combined organic layers were dried over Na_2SO_4, filtered and concentrated *in vacuo*. The residue obtained was purified by flash column chromatography on silica (eluent: 40:55:5 Hexanes:EtOAc:Et_3N) to afford the title compound **12** (3.26 g, 11.89 mmol, 62%) with spectral data in accord with that previously reported in the literature.[1]

Physical State: Light yellow oil.

TLC R_f = 0.30 (40:55:5 Hexanes:EtOAc:Et_3N; CAM)

^{1}H NMR (500 MHz, Toluene-D_8): δ 3.77-3.75 (m, 2H), 3.72-3.70 (m, 2H), 3.10-3.08 (m, 2H), 2.76 (br s, 2H), 2.47-2.45 (m, 2H), 2.07-2.02 (m, 5H). 0.97 (s, 9H), 0.07 (s, 6H).

^{13}C NMR (126 MHz, Toluene-D_8)*: δ 73.1, 62.0, 55.8, 54.5, 45.5, 26.1, 18.5, -5.2.

*Variable temperature NMR experiments were performed to resolve methylene peaks adjacent to N-O bond.[6-9]

Synthesis of 2-((4-methylpiperazin-1-yl)oxy)ethan-1-ol (**13**) *via* Literature Procedure[1]

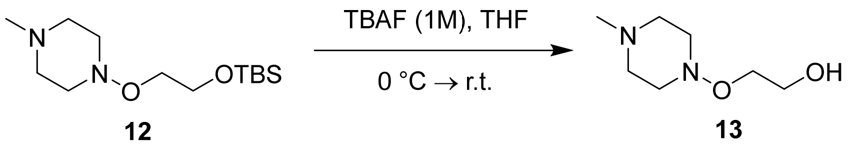

Compound **12** (3.20 g, 11.67 mmol, 1.0 eq.) was dissolved in anhydrous THF (140 mL) at 0 °C and TBAF (1M in THF) (23.34 mL, 23.34 mmol, 2.0 eq.) was added dropwise to this mixture. The solution was warmed to r.t. and stirred for1 h. After which, the mixture was concentrated *in vacuo* and the residue obtained was purified by flash column chromatography (eluent: 90:10 EtOAc:Et_3N) to afford the title compound **13** (1.21 g, 7.56 mmol, 65%) with spectral data in accord with that previously reported in the literature.[1]

Physical State: Yellow oil.

TLC R_f = 0.10 (90:10 EtOAc:Et_3N; CAM)

^{1}H NMR (500 MHz, Toluene-D_8): δ 3.68-3.66 (m, 2H), 3.65-3.61 (m, 2H), 3.01 (br d, *J* = 10.3 Hz, 2H), 2.69-2.64 (m, 2H), 2.40 (br d, *J* = 11.4 Hz, 2H), 1.96 (s, 3H), 1.94-1.89 (m, 2H).

^{13}C NMR (126 MHz, Toluene-D_8)*: δ 72.2, 63.2, 55.4, 54.2, 45.3.

*Variable temperature NMR experiments were performed to resolve methylene peaks adjacent to N-O bond.[6-9]

Synthesis of 4-chloro-6-methoxy-7-(2-((4-methylpiperazin-1-yl)oxy)ethoxy)quinolone-3-carbonitrile (**15**)

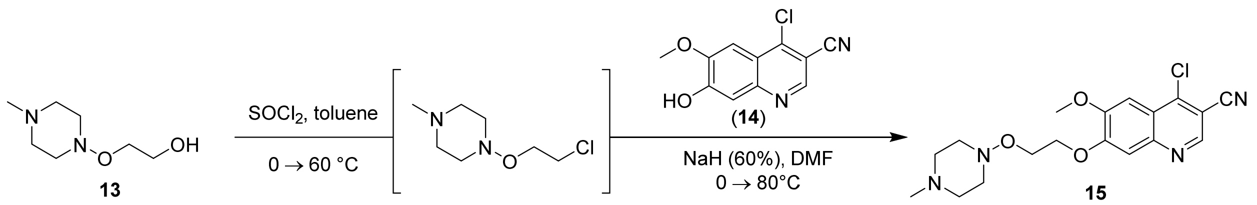

To a stirred solution of **13** (1.15 g, 7.18 mmol, 1.0 eq.) in anhydrous toluene (30 mL) at 0 °C was added $SOCl_2$ (1.05 mL, 14.36 mmol, 2.5 eq.) dropwise. The solution was stirred for 5 min, after which, the mixture was heated to 60 °C with stirring for 3 h. The reaction mixture was then cooled to r.t. and the solvent was removed *in vacuo*. The residue obtained was dissolved in EtOAc (75 mL) and washed with a saturated aq. K_2CO_3 soln. (2x, 75 mL). The organic layers were combined, dried over Na_2SO_4, filtered and concentrated *in vacuo*. The so obtained crude alkyl chloride was used directly in the next step without further purification.

To a stirred solution of 4-chloro-7-hydroxy-6-methoxyquinoline-3-carbonitrile (**14**) (2.53 g, 10.77 mmol, 1.5 eq.) in anhydrous DMF (30 mL) at 0 °C was added NaH (60% dispersion in mineral oil) (429 mg, 10.77 mmol, 1.5 eq.) and the solution was stirred for 30 min with gradual warming to r.t. After which, the crude alkyl chloride obtained in the previous step was added dropwise as a solution in anhydrous DMF (5 mL) at r.t. After stirring for 5 min, the solution was heated to 80 °C with stirring for 6 h. The reaction mixture was then cooled to r.t. and the solvent removed *in vacuo*. The resulting residue was co-concentrated with toluene (3x, 50 mL) and subsequently re-dissolved in EtOAc (75 mL). The solution washed successively with saturated K_2CO_3 soln. (2x, 75 mL) and aq. brine (2x, 75 mL) and the organic layer was dried over Na_2SO_4, filtered and concentrated *in vacuo*. The residue obtained was purified by flash column chromatography (eluent: 90:5:5 EtOAc:MeOH:Et_3N) to afford the title compound (**15**) (950 mg, 2.53 mmol, 35% over 2 steps).

Physical State: Light yellow powder.

TLC R_f = 0.50 (90:5:5 EtOAc:MeOH:Et_3N; CAM, UV)

^{1}H NMR (500 MHz, $CDCl_3$): δ 8.76 (s, 1H), 7.45 (s, 1H), 7.40 (s, 1H), 4.37 (t, *J* = 5.0 Hz, 2H), 4.16 (t, *J* = 5.0 Hz, 2H), 4.05 (s, 3H), 3.21 (br d, *J* = 10.1 Hz, 2H), 2.78-2.76 (m, 4H), 2.27-2.25 (m, 5H).

^{13}C NMR (126 MHz, $CDCl_3$)*: δ 155.0, 152.5, 148.3, 147.2, 143.9, 121.2, 115.6, 109.4, 105.6, 102.2, 69.3, 67.7, 56.5, 55.5, 54.1, 45.6.

HRMS-ESI (m/z): $[M+H]^+$ calculated for $[C_{18}H_{22}N_4O_3{}^{35}Cl]^+$: 377.1375, found 377.1371.

m.p: 139.1 - 141.1 °C (mean of *n* = 3 determinations)

*Variable temperature NMR experiments were performed to resolve methylene peaks adjacent to N-O bond.[6-9]

Synthesis of 4-((2,4-dichloro-5-methoxyphenyl)amino)-6-methoxy-7-(2-((4-methylpiperazin-1-yl)oxy)ethoxy)quinoline-3-carbonitrile (**9**)

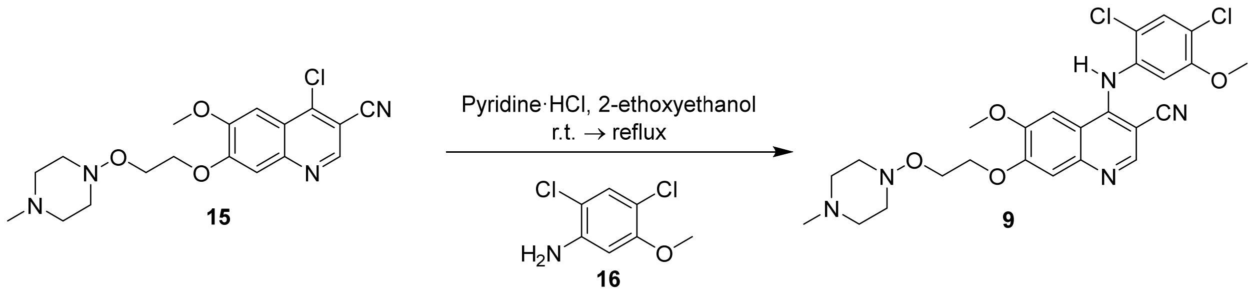

To a stirred solution of **15** (925 mg, 2.46 mmol, 1.0 eq.) in 2-ethoxyethanol (12.5 mL) at r.t. was added Pyridine·HCl (569 mg, 4.92 mmol, 2.0 eq.) followed by 2,4-dichloro-5-methoxyaniline (**16**) (709 mg, 3.69 mmol, 1.5 eq.). The solution was heated to reflux (135 °C) with stirring for 10 h. After which, the reaction mixture was cooled to r.t. and the solvent removed *in vacuo*. The residue obtained was co-concentrated with toluene (3x, 30 mL). After which, the residue was purified by flash column chromatography on silica (eluent: 90:5:5 EtOAc:MeOH:Et_3N to 85:10:5 EtOAc:MeOH:Et_3N) to afford the title compound (**9**) (374 mg, 0.704 mmol, 29%).

Physical State: Light yellow solid.

TLC R_f = 0.20 (90:5:5 EtOAc:MeOH:Et_3N; CAM, UV)

^{1}H NMR (500 MHz, $CDCl_3$): δ 8.71 (s, 1H), 7.49 (s, 1H), 7.44 (s, 1H), 6.90 (s, 1H), 6.73 (s, 1H), 6.46 (s, 1H), 4.36 (t, J = 5.0 Hz, 2H), 4.15 (t, J = 5.0 Hz, 2H), 3.77 (s, 3H), 3.67 (s, 3H), 3.23-3.21 (m, 2H), 2.78 (br s, 4H), 2.28-2.24 (m, 5H).

^{13}C NMR (126 MHz, $CDCl_3$)*: δ 154.4, 154.0, 150.5, 150.0, 147.72, 147.66, 137.0, 130.7, 118.6, 117.5, 116.5, 115.0, 110.1, 105.8, 101.3, 94.2, 69.4, 67.5, 56.7, 56.2, 55.4, 53.9, 45.5.

HRMS-ESI (m/z): $[M+H]^+$ calculated for $[C_{25}H_{28}O_4N_5{}^{35}Cl_2]$: 532.1518, found: 532.1505.

m.p: 183.1 - 184.7 °C (mean of n = 3 determinations)

*Variable temperature NMR experiments were performed to resolve methylene peaks adjacent to N-O bond.[6-9]

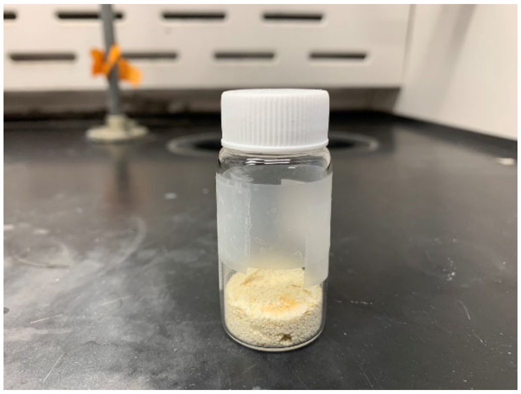

Figure S5. 374 mg of inhibitor (**9**).

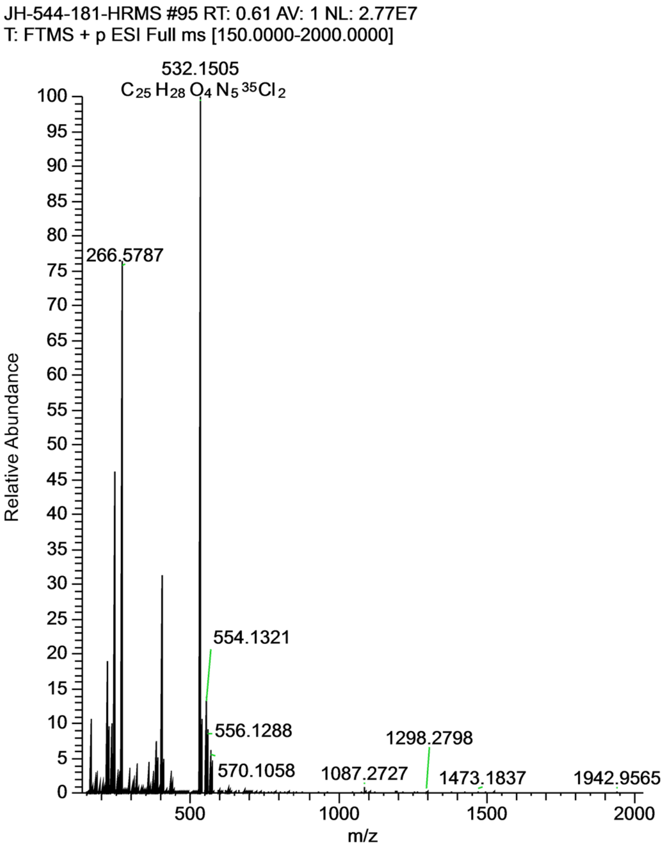

Figure S6. HRMS of inhibitor (**9**).

Synthesis of 3-(2-methoxyphenoxy)propyl-4-methylbenzenesulfonate (**22**)

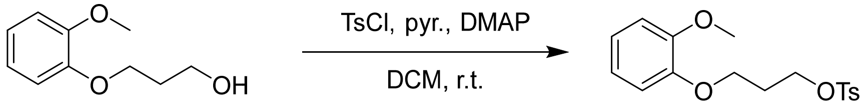

22

Alcohol[2] (600 mg, 3.29 mmol, 1.0 eq.) was dissolved in anhydrous DCM (20 mL). Pyridine (530 µL, 6.58 mmol, 2.0 eq.) was then added to the mixture followed by DMAP (40 mg, 0.329 mmol, 0.1 eq) and the solution was stirred for 5 min. After which, TsCl (1.25 g, 6.58 mmol, 2.0 eq.) was added portion-wise over a period of 5 min and the solution stirred for 12 h. The reaction mixture was then diluted with DCM (20 mL) and quenched via addition of H_2O (20 mL). The layers were separated and the organic layer washed successively with 1 N HCl (1x, 20 mL), aq. $NaHCO_3$ (1x, 20 mL) and aq. brine (1x, 20 mL). The organic layer was dried over Na_2SO_4, filtered and concentrated *in vacuo*. The residue obtained was purified by flash column chromatograph on silica (eluent: 25:75 EtOAc:Hexanes) to afford the title compound (**22**) (482 mg, 1.43 mmol, 44%) with spectral data in accord with that previously reported in the literature.[2]

Physical State: Yellow oil.

TLC R_f = 0.20 (25:75 EtOAc:Hexanes; UV, CAM)

^{1}H NMR (500 MHz, $CDCl_3$): δ 7.76 (d, *J* = 8.1 Hz, 2H), 7.28-7.22 (m, 2H), 6.94-6.91 (m, 1H), 6.89-6.84 (m, 2H), 6.79-6.77 (m, 1H), 4.28 (t, *J* = 6.0 Hz, 2H), 4.00 (t, *J* = 6.0 Hz, 2H), 3.80 (s, 3H), 2.39 (s, 3H), 2.16 (p, *J* = 6.0 Hz, 2H).

^{13}C NMR (126 MHz, $CDCl_3$): δ 149.6, 148.1, 144.8, 133.0, 129.9, 128.0, 121.6, 121.0, 113.8, 112.0, 67.5, 64.6, 55.9, 29.1, 21.8.

Synthesis of 1-(3-(2-methoxyphenoxy)propyl)-4-methylpiperazine (**17**).

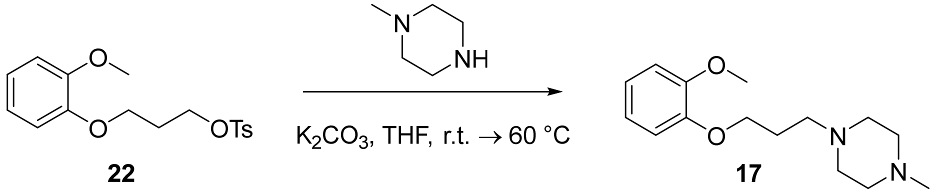

Compound **22** (450 mg, 1.34 mmol, 1.0 eq.) was dissolved in anhydrous THF (14 mL) at r.t. *N*-methylpiperazine (743 μL, 6.70 mmol, 5.0 eq.) was then added followed by K_2CO_3 (370 g, 2.68 mmol, 5.0 eq.) and the solution heated to 60 °C with stirring for 14 h. The mixture was then cooled to r.t. and diluted with EtOAc (30 mL). The organic layer was washed with 1N NaOH (2x, 30 mL), dried over Na_2SO_4, filtered and concentrated *in vacuo*. The residue obtained was purified by flash column chromatography on silica (eluent: 10:90 Et_3N:EtOAc) to afford the title compound (**17**) (154 mg, 0.582 mmol, 43% yield).

Physical State: Light yellow oil.

TLC R_f = 0.25 (10:90 Et_3N:EtOAc; UV, CAM)

^{1}H NMR (500 MHz, $CDCl_3$): δ 6.92-6.86 (m, 4H), 4.07 (t, J = 6.7 Hz, 2H), 3.85 (s, 3H), 2.72-2.29 (m, 10H), 2.28 (s, 3H), 2.05-1.98 (m, 2H).

^{13}C NMR (126 MHz, $CDCl_3$): δ 149.7, 148.6, 121.2, 121.0, 113.6, 112.1, 67.6, 56.1, 55.3, 55.2, 55.3, 46.2, 26.8.

HRMS-ESI (m/z): $[M+H]^+$ calculated for $[C_{15}H_{25}N_2O_2]^+$: 265.1911, found: 265.1909.

Synthesis of 1-(2-(2-methoxyphenoxy)ethoxy)-4-methylpiperazine (**18**).

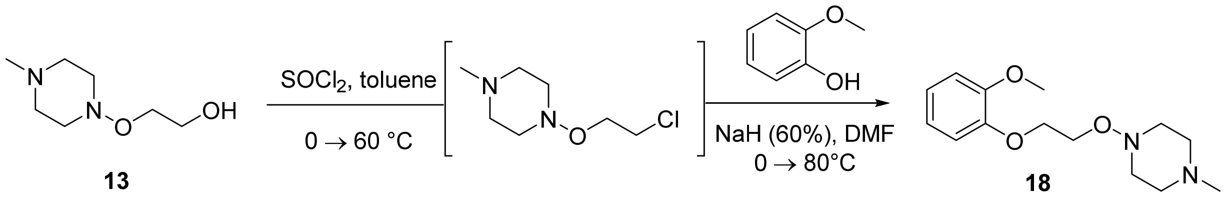

To a stirred solution of **13** (575 mg, 3.59 mmol, 1.0 eq.) in anhydrous toluene (15 mL) at 0 °C was added $SOCl_2$ (651 µL, 8.98 mmol, 2.5 eq.) dropwise. The solution was stirred for 5 min, after which, the mixture was heated to 60 °C with stirring for 3 h. The reaction mixture was then cooled to r.t. and the solvent removed *in vacuo*. The residue obtained was dissolved in EtOAc (50 mL) and washed with a saturated aq. K_2CO_3 soln. (2x, 30 mL). The organic layers were combined, dried over Na_2SO_4, filtered and concentrated *in vacuo*. The so obtained crude alkyl chloride was used directly in the next step without further purification.

To a stirred solution of 2-methoxyphenol (800 µL, 7.18 mmol, 2.0 eq.) in anhydrous DMF (15 mL) at 0 °C was added NaH (60% dispersion in mineral oil) (286 mg, 7.18 mmol, 2.0 eq.) and the solution was stirred for 30 min with gradual warming to r.t. After such time, the crude alkyl chloride obtained in the previous step was added dropwise as a solution in anhydrous DMF (3 mL) at r.t. After stirring for 5 min, the solution was heated to 80 °C with stirring for 16 h. The reaction mixture was then cooled to r.t. and the solvent removed *in vacuo*. The residue obtained was then co-concentrated with toluene (3x, 15 mL), dissolved in EtOAc (50 mL) and washed with 1N NaOH (2x, 30 mL). The organic layer was dried over Na_2SO_4, filtered and concentrated *in vacuo*. The residue obtained was purified by flash column chromatography on silica (eluent: 3:6:1 Hexanes:EtOAc:Et_3N) to afford the title compound (**18**) (246 mg, 0.924 mmol, 26% yield over 2 steps).

Physical state: Yellow oil.

TLC R_f = 0.50 (3:6:1 Hexanes:EtOAc:Et_3N; UV, CAM)

^{1}H NMR (500 MHz, $CDCl_3$): δ 6.98-6.83 (m, 4H), 4.18 (dd, *J* =5.8, 4.6 Hz, 2H), 4.09-4.02 (m, 2H), 3.85 (s, 3H), 3.22-3.20 (m, 2H), 2.76-2.74 (m, 4H), 2.26-2.20 (m, 5H).

^{13}C NMR (126 MHz, $CDCl_3$)*: δ 149.9, 148.6, 121.6, 121.0, 114.3, 112.3, 70.0, 67.6, 56.1, 55.5, 54.3, 45.6.

HRMS-ESI (m/z): $[M+H]^+$ calculated for $[C_{14}H_{23}N_2O_3]^+$: 267.1703, found: 267.1694.

*Variable temperature NMR experiments were performed to resolve methylene peaks adjacent to N-O bond.[6-9]

5. Catalog of Spectra

^{1}**H NMR** (500 MHz, Toluene-D$_8$) spectrum of 1-(2-((*tert*-butyldimethylsilyl)oxy)ethoxy)-4-methylpiperazine (**12**).

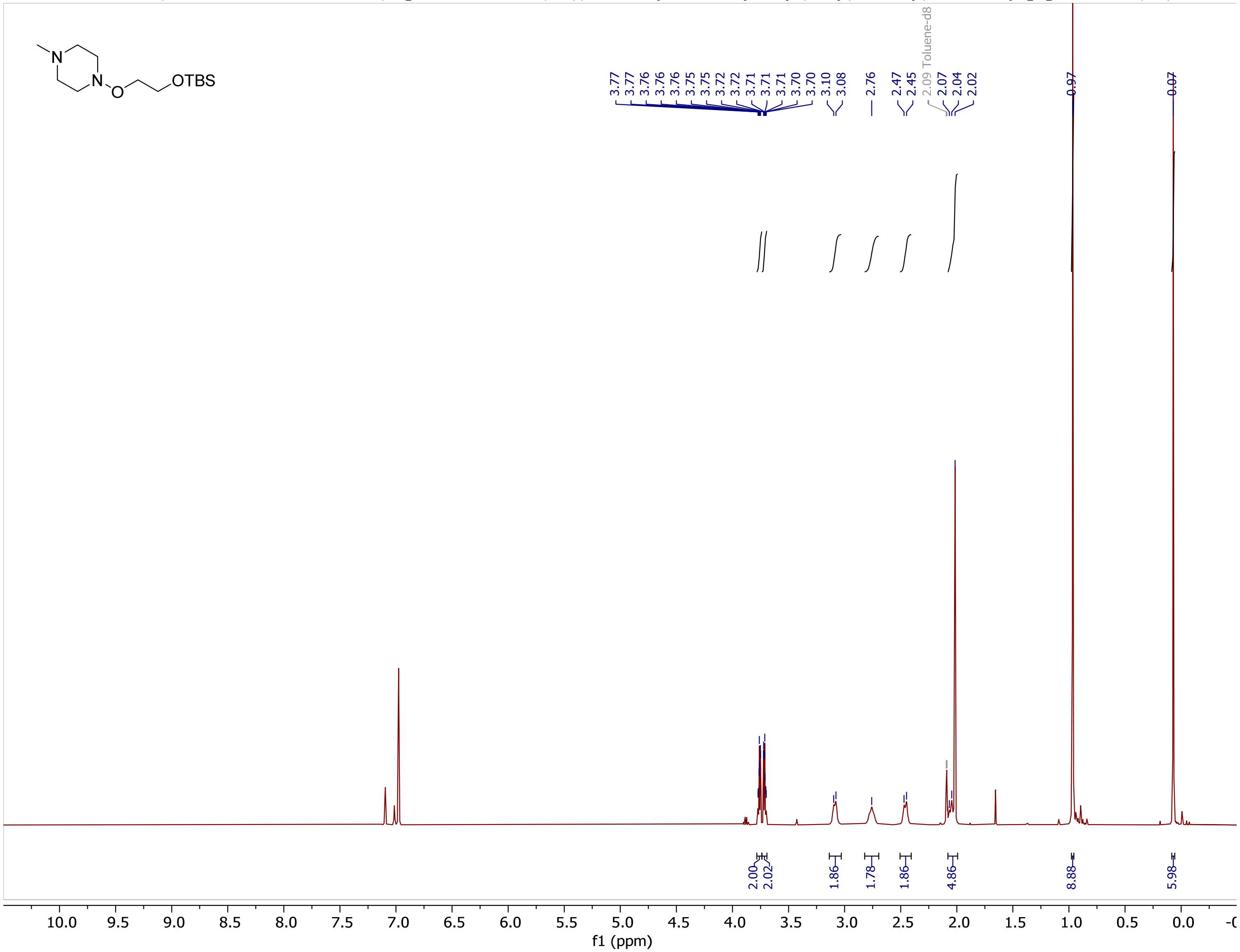

^{13}**C NMR** (126 MHz, Toluene-D$_8$) spectrum of 1-(2-((*tert*-butyldimethylsilyl)oxy)ethoxy)-4-methylpiperazine (**12**).

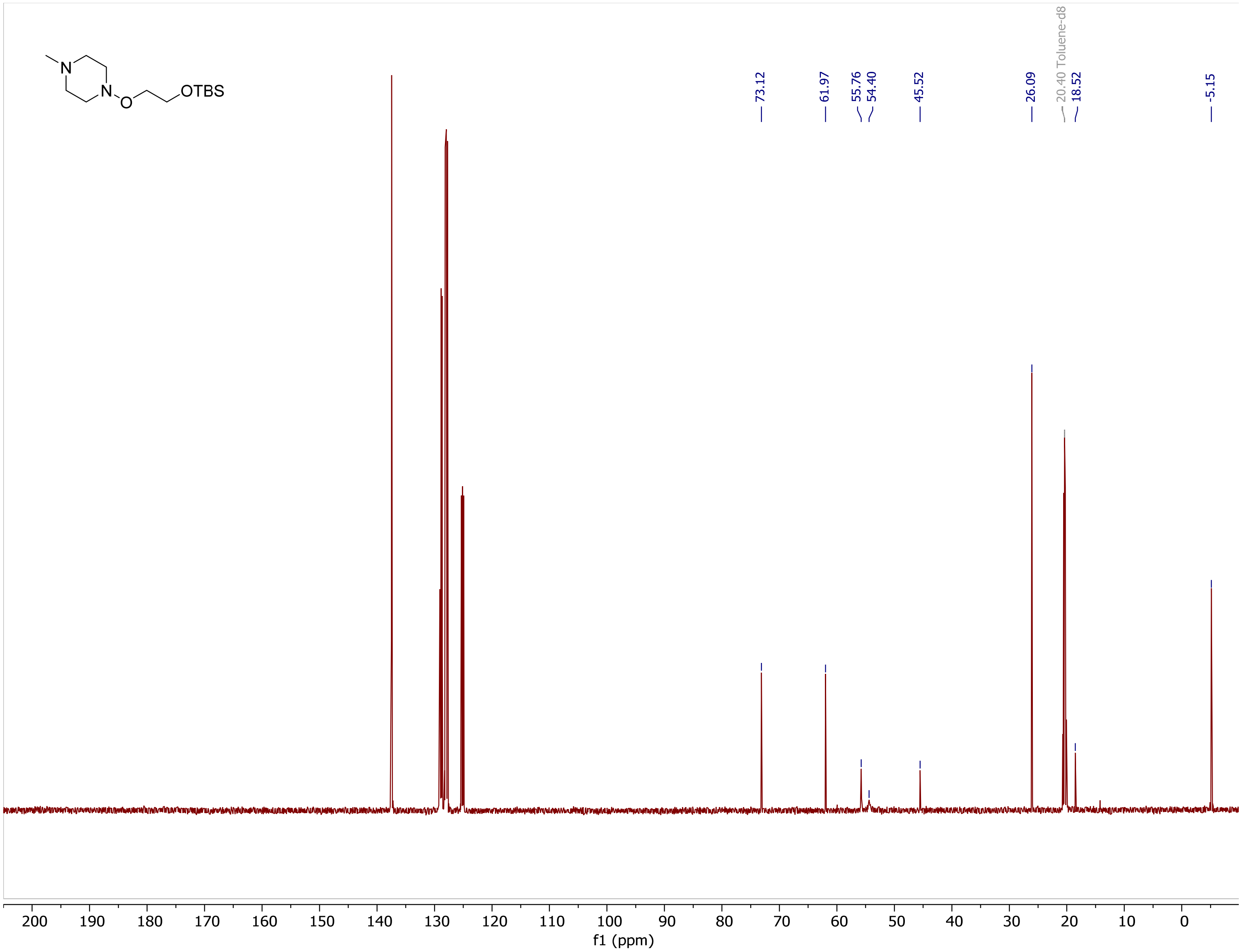

HSQC NMR (500 MHz, Toluene-D_8) spectrum of 1-(2-((*tert*-butyldimethylsilyl)oxy)ethoxy)-4-methylpiperazine (**12**).

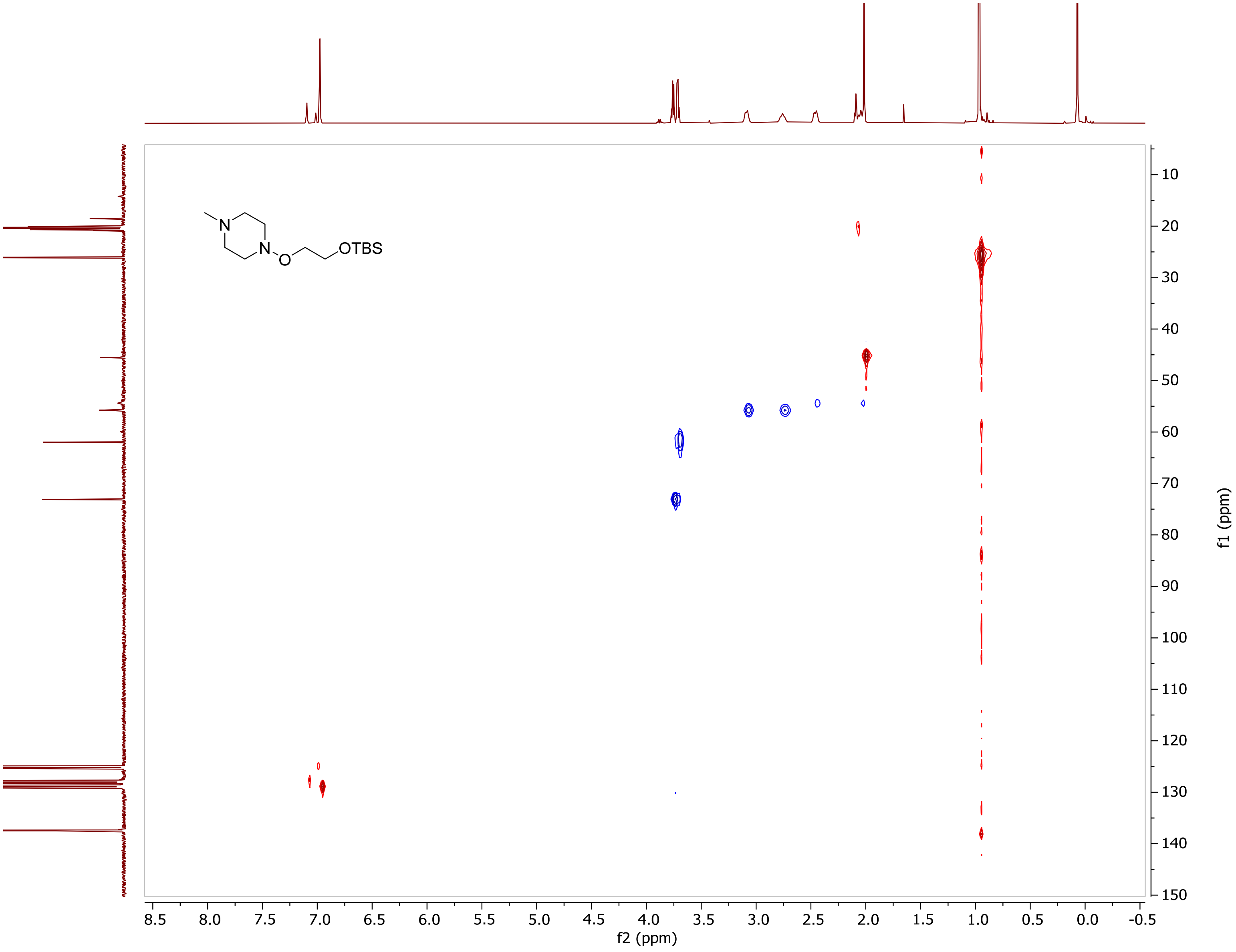

Expanded region of stacked variable temperature **^{13}C NMR** (126 MHz, Toluene-D$_8$) spectrum of 1-(2-((*tert*-butyldimethylsilyl)oxy)ethoxy)-4-methylpiperazine (**12**) at a) 343 K and b) 298 K.

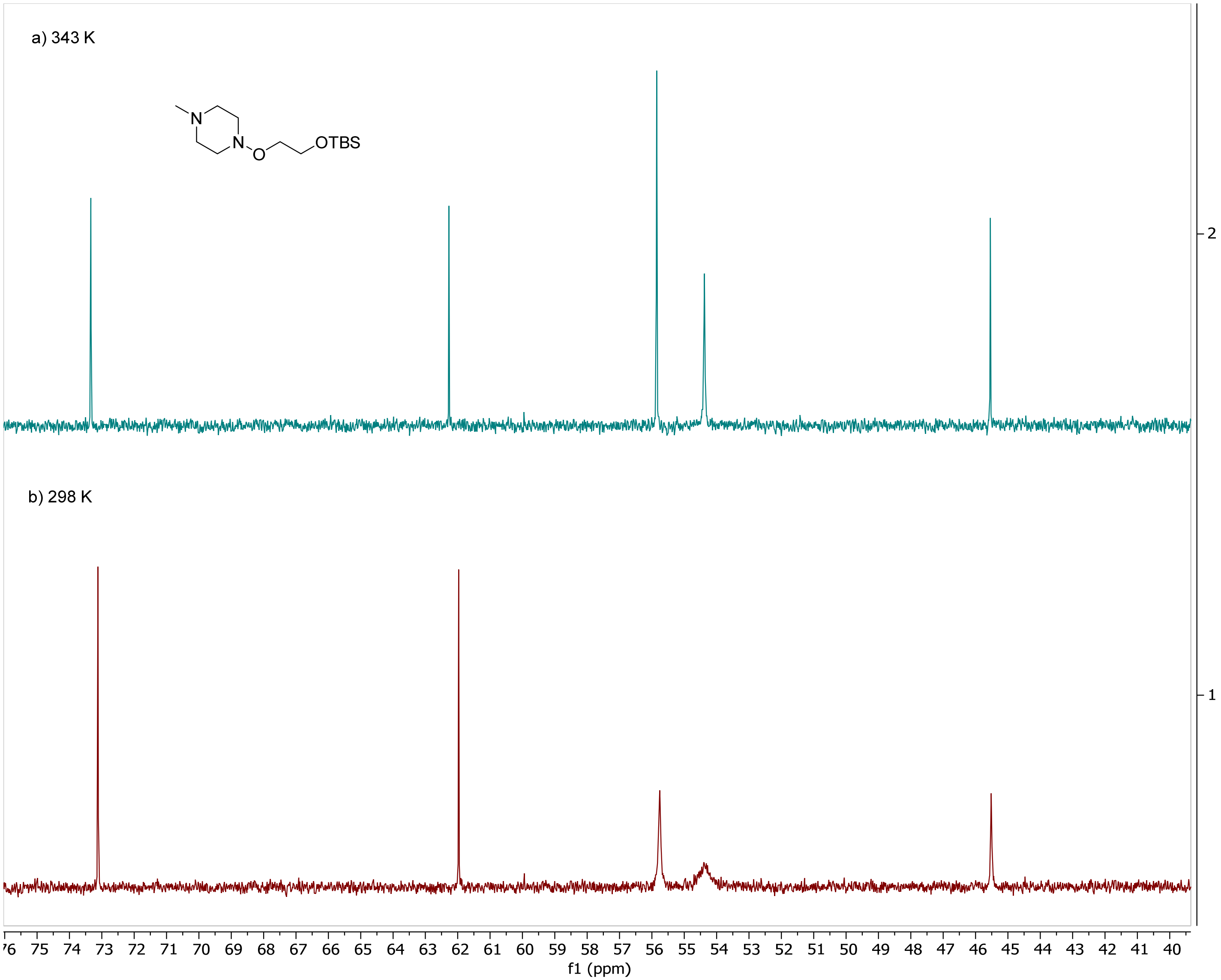

[1]**H NMR** (500 MHz, Toluene-D$_8$) spectrum of 2-((4-methylpiperazin-1-yl)oxy)ethan-1-ol (**13**).

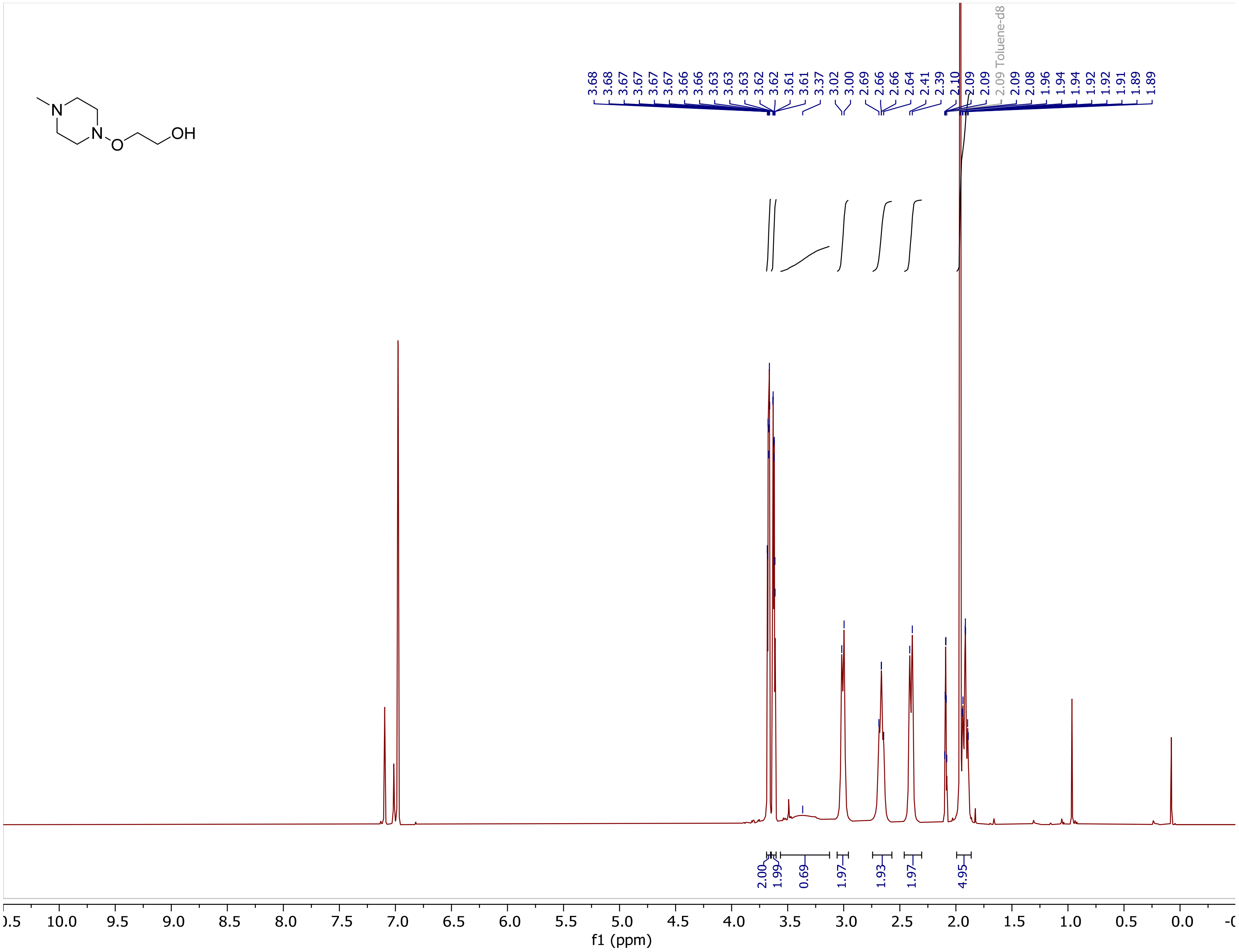

^{13}C NMR (126 MHz, Toluene-D$_8$) spectrum of 2-((4-methylpiperazin-1-yl)oxy)ethan-1-ol (**13**).

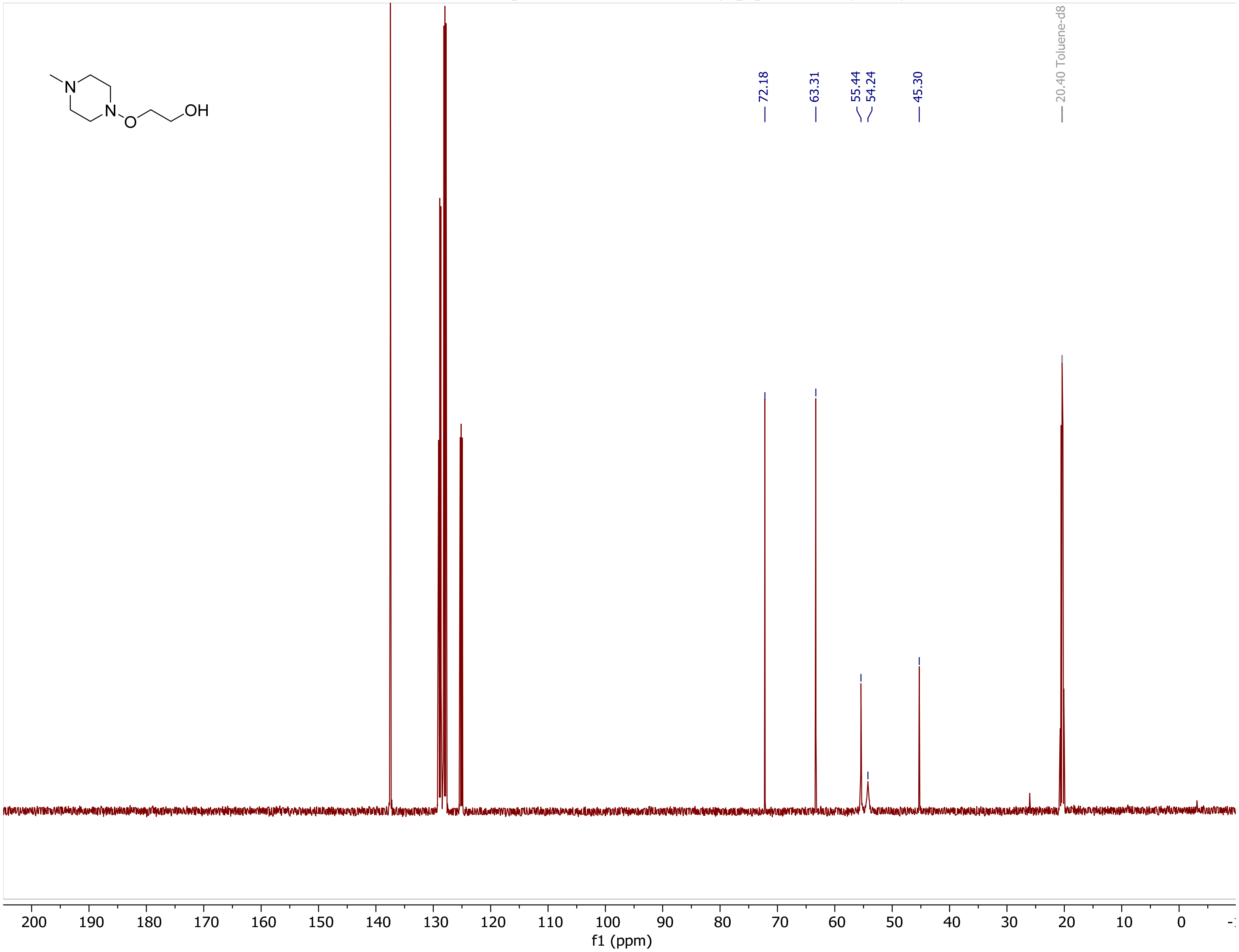

HSQC NMR (500 MHz, Toluene-D_8) spectrum of 2-((4-methylpiperazin-1-yl)oxy)ethan-1-ol (**13**).

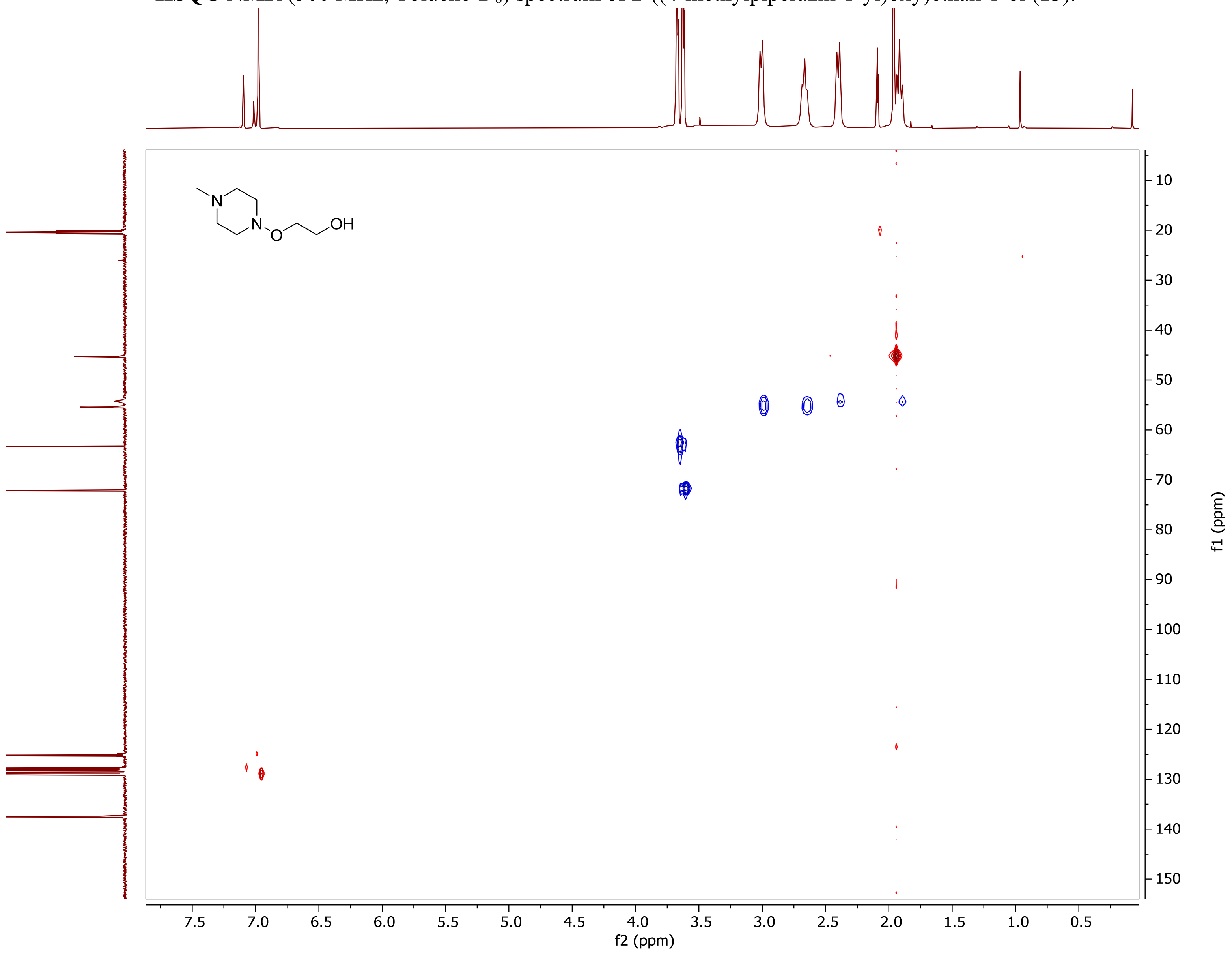

Expanded region of stacked variable temperature ^{13}C **NMR** (126 MHz, Toluene-D$_8$) spectrum of 2-((4-methylpiperazin-1-yl)oxy)ethan-1-ol (**13**) at a) 348 K and b) 298 K.

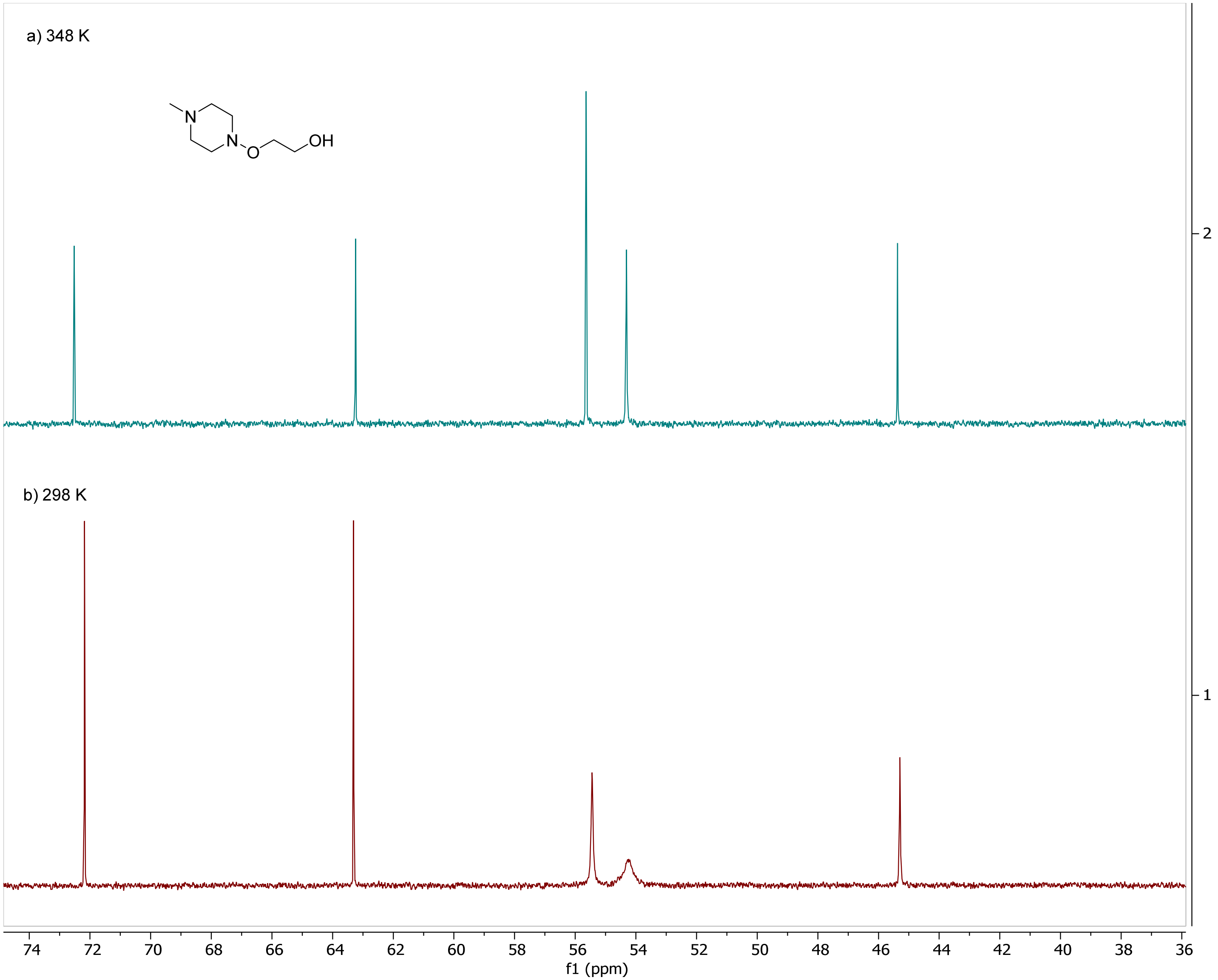

[1]**H NMR** (500 MHz, $CDCl_3$) spectrum of 4-chloro-6-methoxy-7-(2-((4-methylpiperazin-1-yl)oxy)ethoxy)quinolone-3-carbonitrile (**15**).

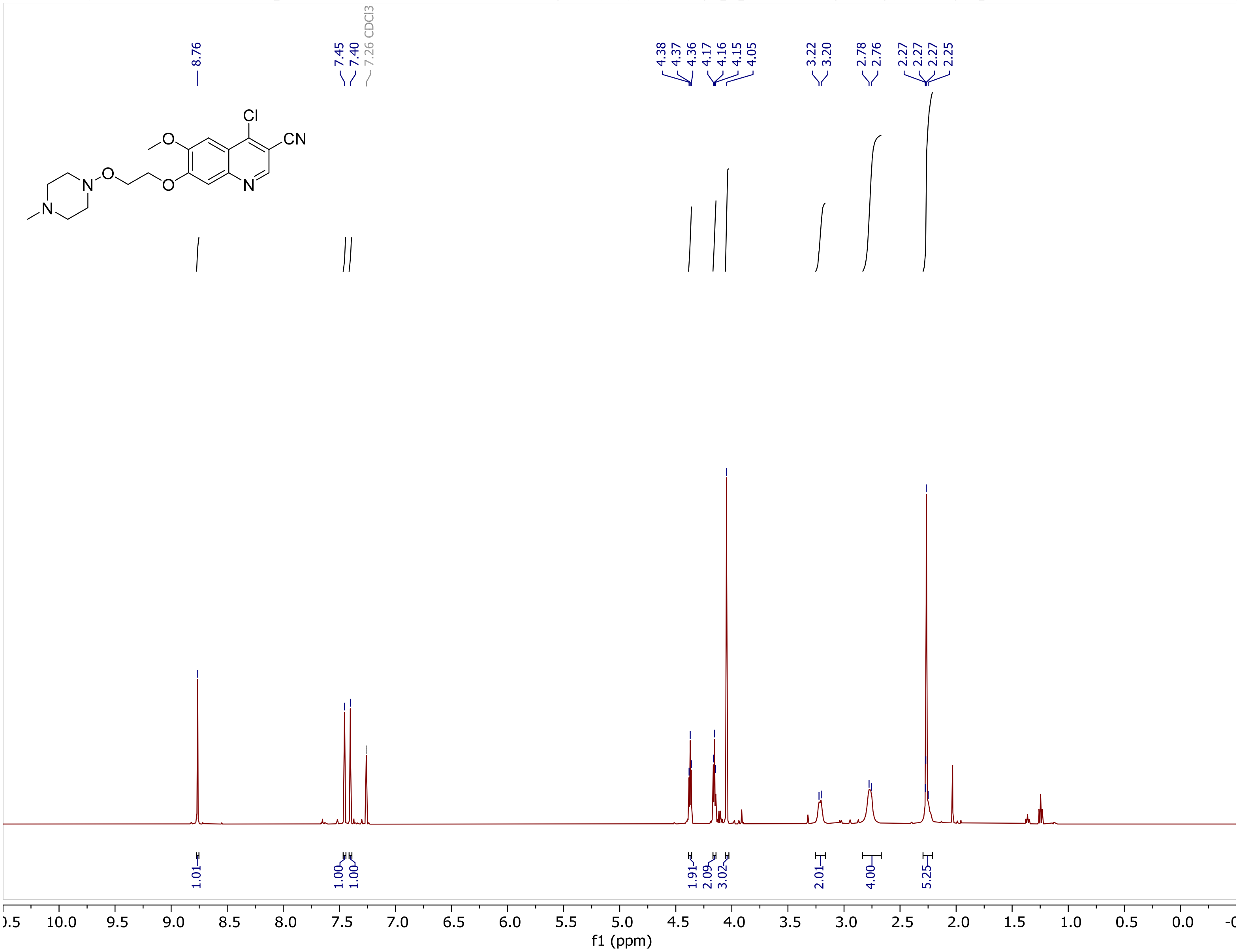

^{13}C NMR (126 MHz, $CDCl_3$) spectrum of 4-chloro-6-methoxy-7-(2-((4-methylpiperazin-1-yl)oxy)ethoxy)quinolone-3-carbonitrile (**15**).

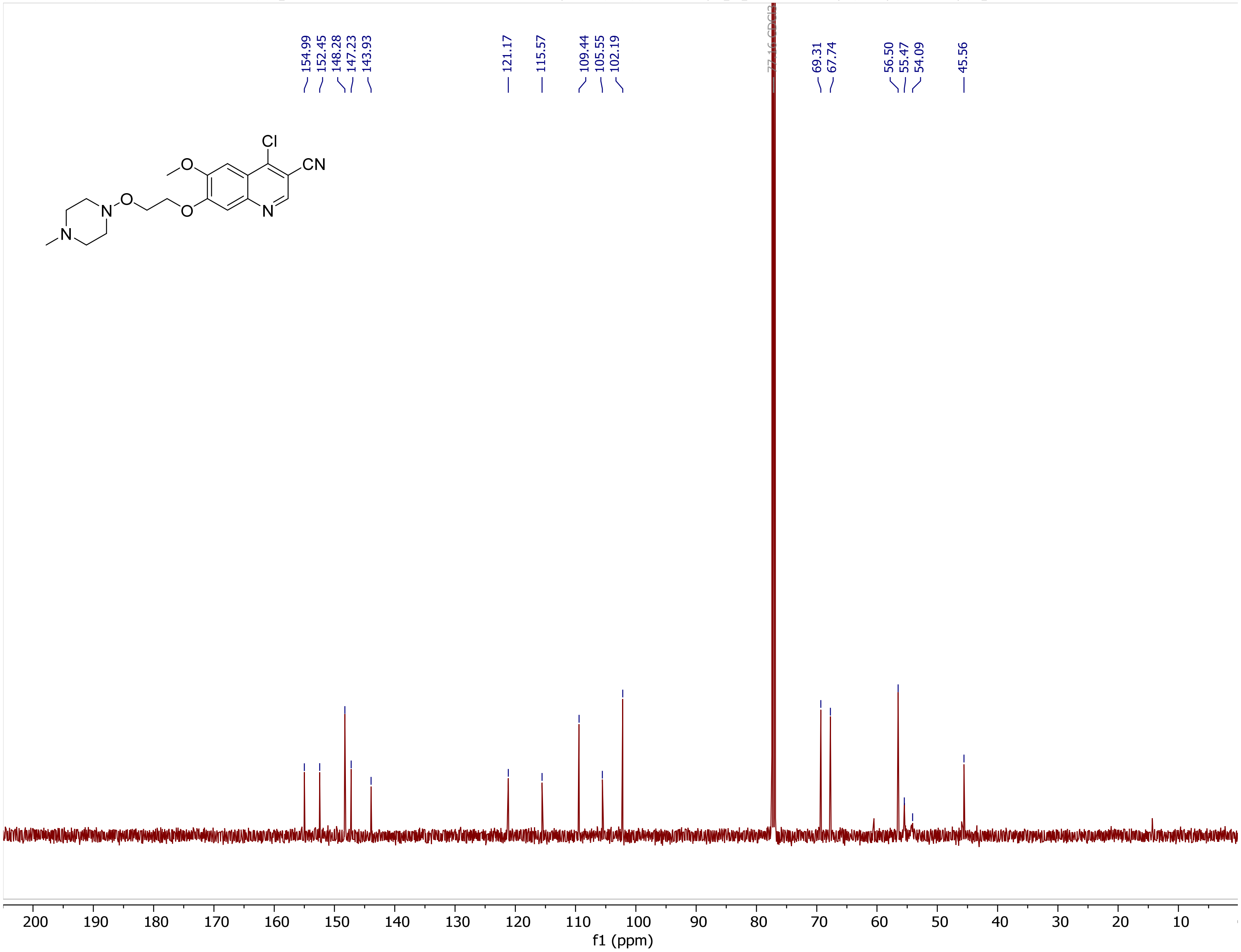

HSQC NMR (500 MHz, $CDCl_3$) spectrum of 4-chloro-6-methoxy-7-(2-((4-methylpiperazin-1-yl)oxy)ethoxy)quinolone-3-carbonitrile (**15**).

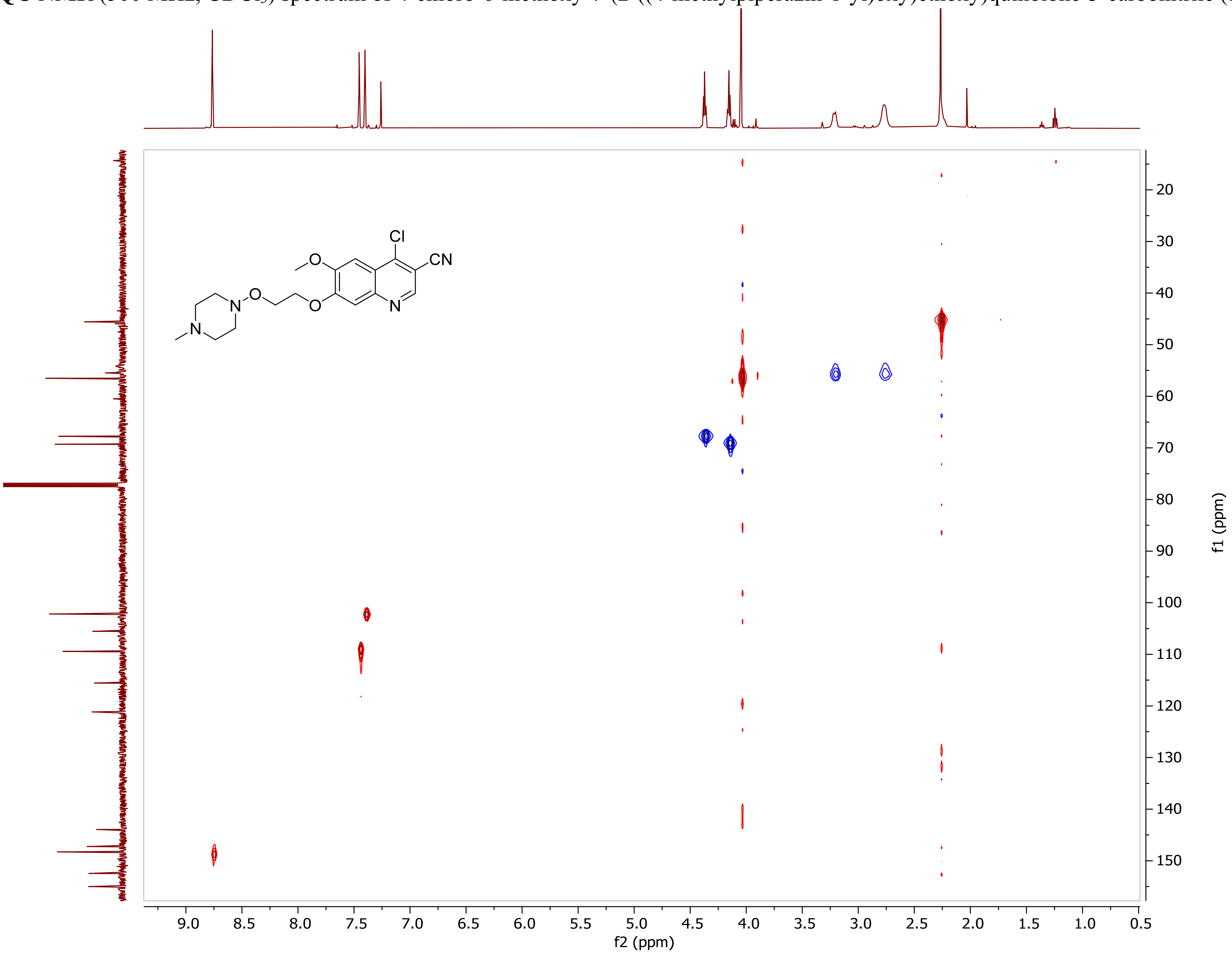

Expanded region of stacked variable temperature **^{13}C NMR** (126 MHz, $CDCl_3$) spectrum of 4-chloro-6-methoxy-7-(2-((4-methylpiperazin-1-yl)oxy)ethoxy)quinolone-3-carbonitrile (**15**) at a) 323 K and b) 298 K.

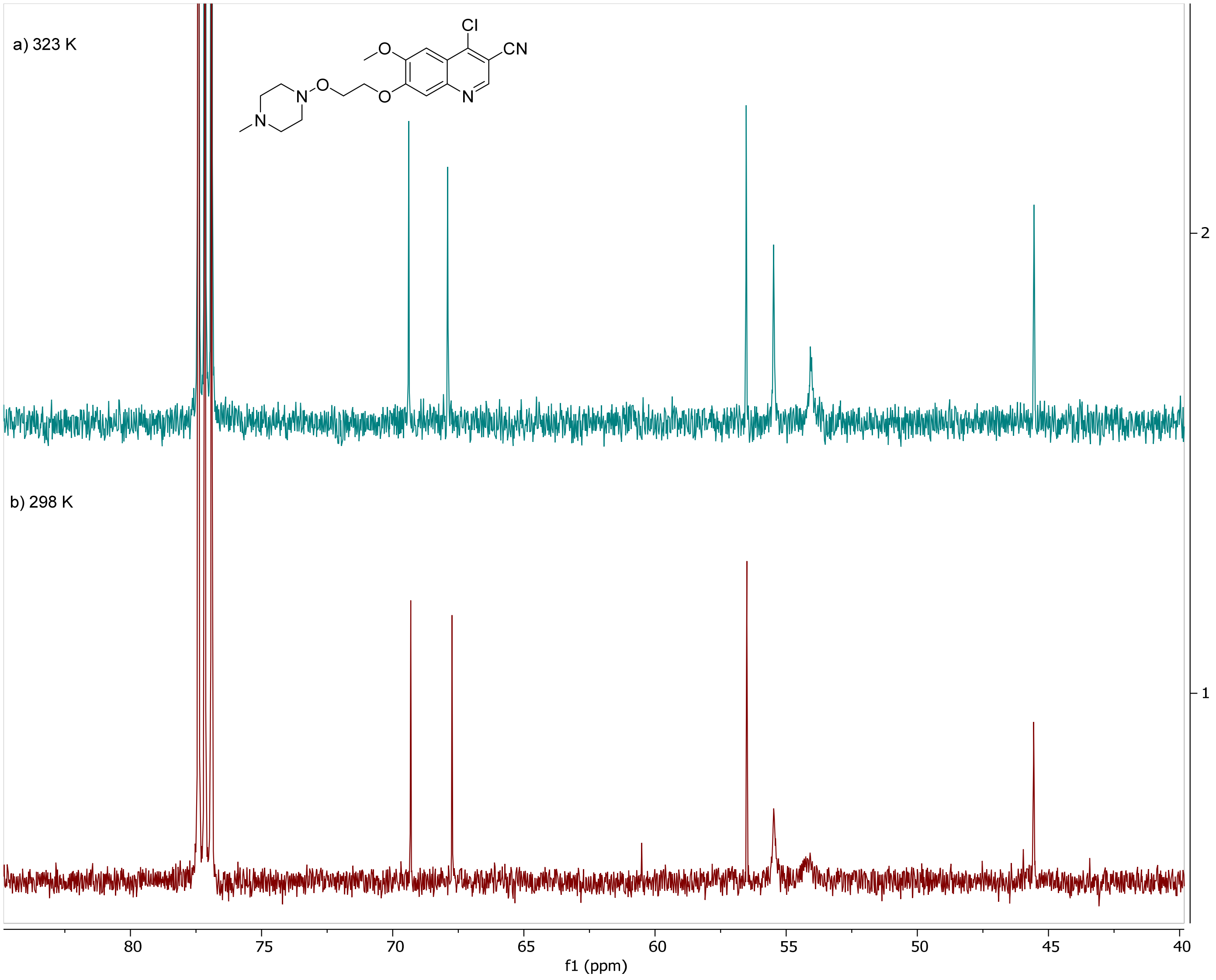

^{1}H NMR (500 MHz, $CDCl_3$) spectrum of 4-((2,4-dichloro-5-methoxyphenyl)amino)-6-methoxy-7-(2-((4-methylpiperazin-1-yl)oxy)ethoxy)quinoline-4-carbonitrile (**9**).

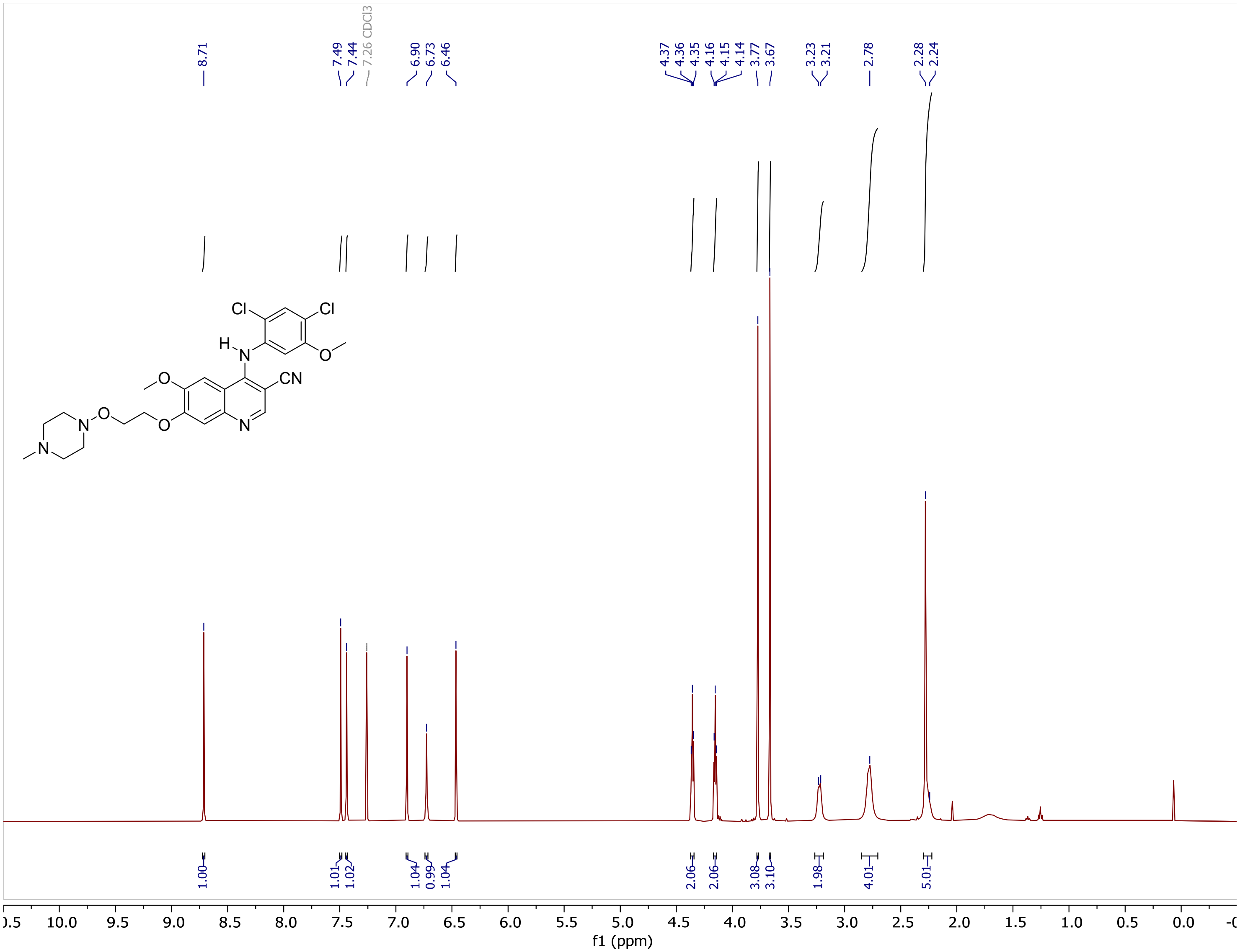

Stacked ^{1}H NMR (500 MHz, $CDCl_3$) spectrum of a) 4-((2,4-dichloro-5-methoxyphenyl)amino)-6-methoxy-7-(2-((4-methylpiperazin-1-yl)oxy)ethoxy)quinoline-4-carbonitrile (**9**) and b) Bosutinib (**3**).

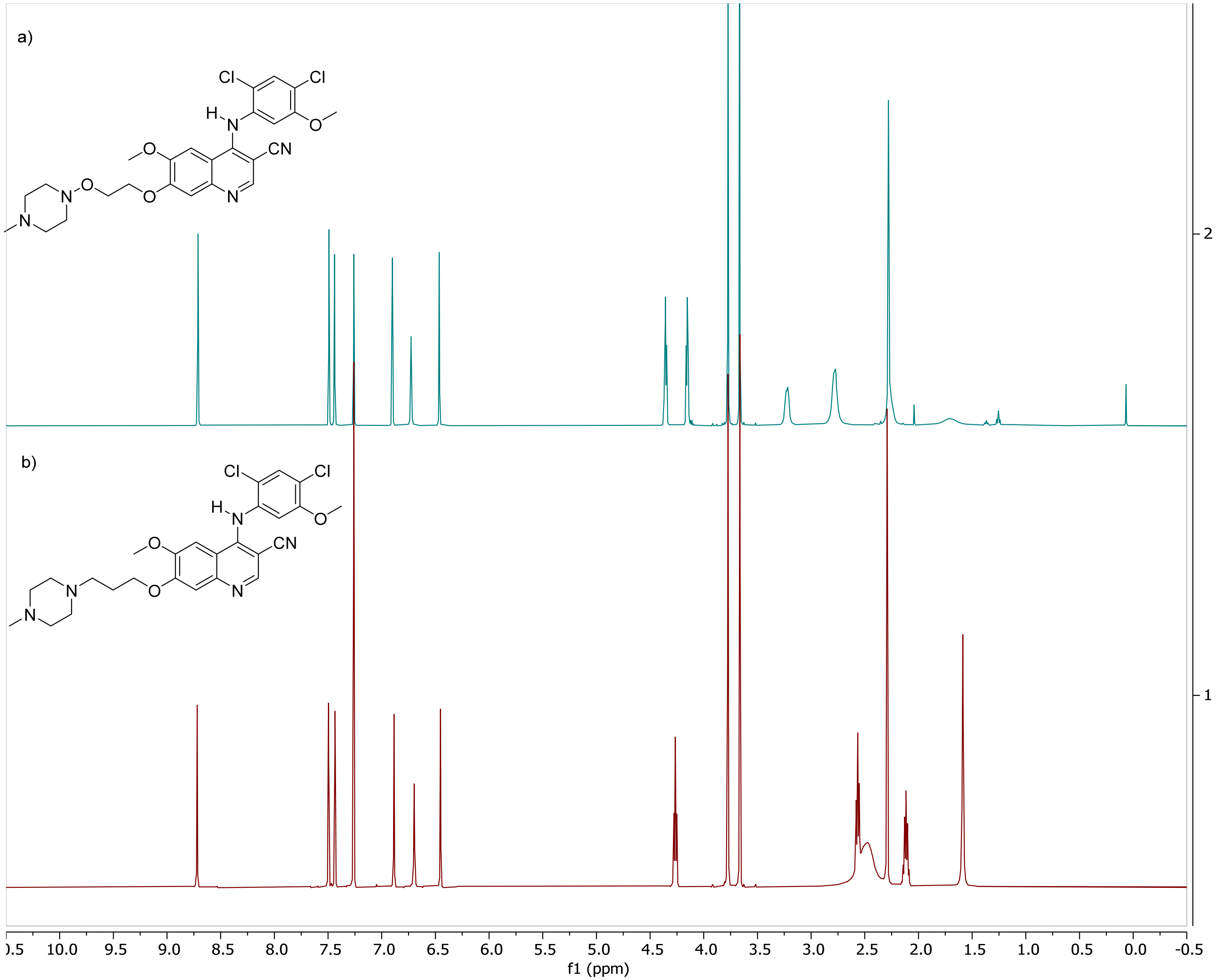

^{13}C NMR (126 MHz, $CDCl_3$) spectrum of 4-((2,4-dichloro-5-methoxyphenyl)amino)-6-methoxy-7-(2-((4-methylpiperazin-1-yl)oxy)ethoxy)quinoline-4-carbonitrile (**9**) (294 K).

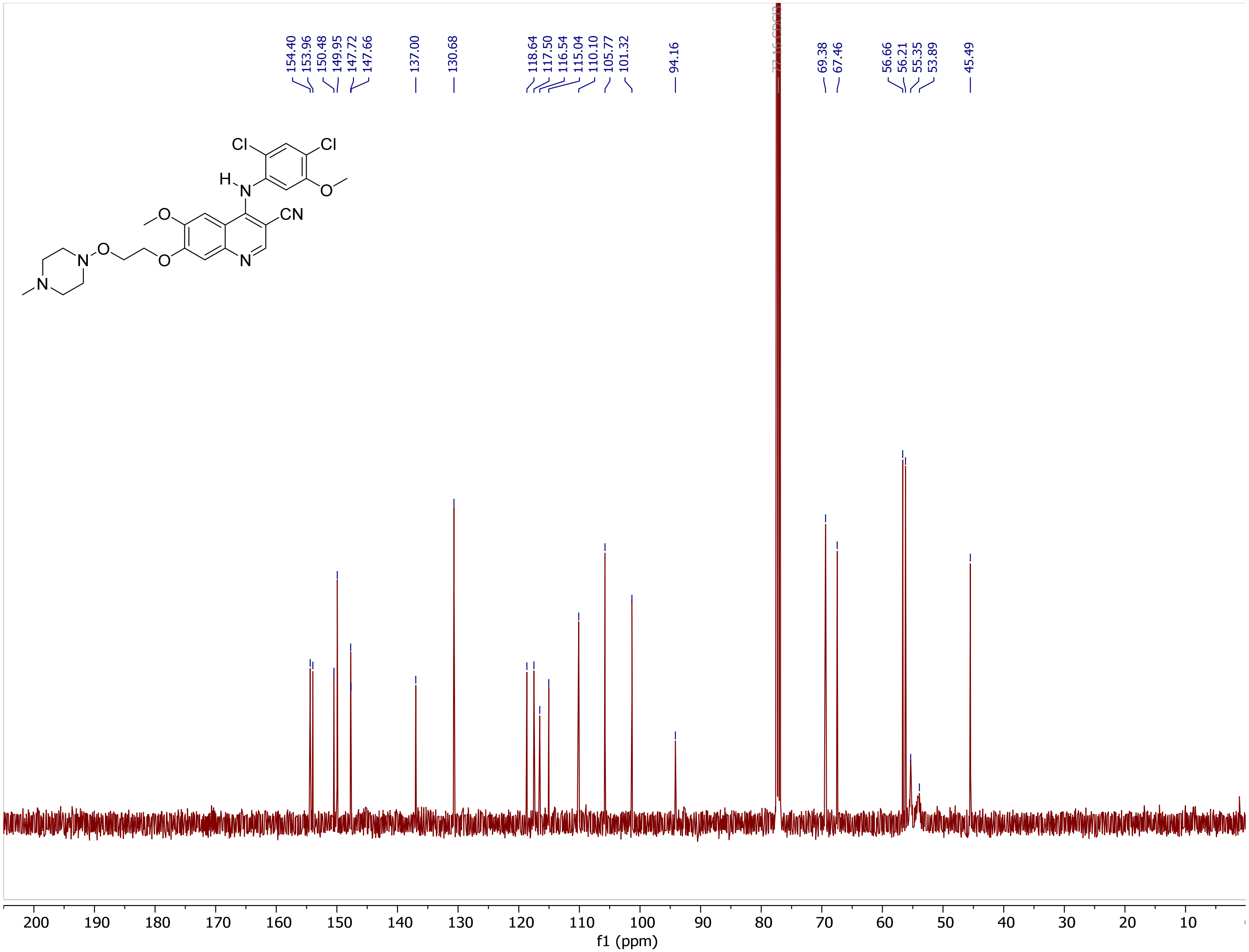

Expanded region of stacked variable temperature **^{13}C NMR** (126 MHz, $CDCl_3$) spectrum of 4-((2,4-dichloro-5-methoxyphenyl)amino)-6-methoxy-7-(2-((4-methylpiperazin-1-yl)oxy)ethoxy)quinoline-4-carbonitrile (**9**) at a) 328 K and b) 294 K.

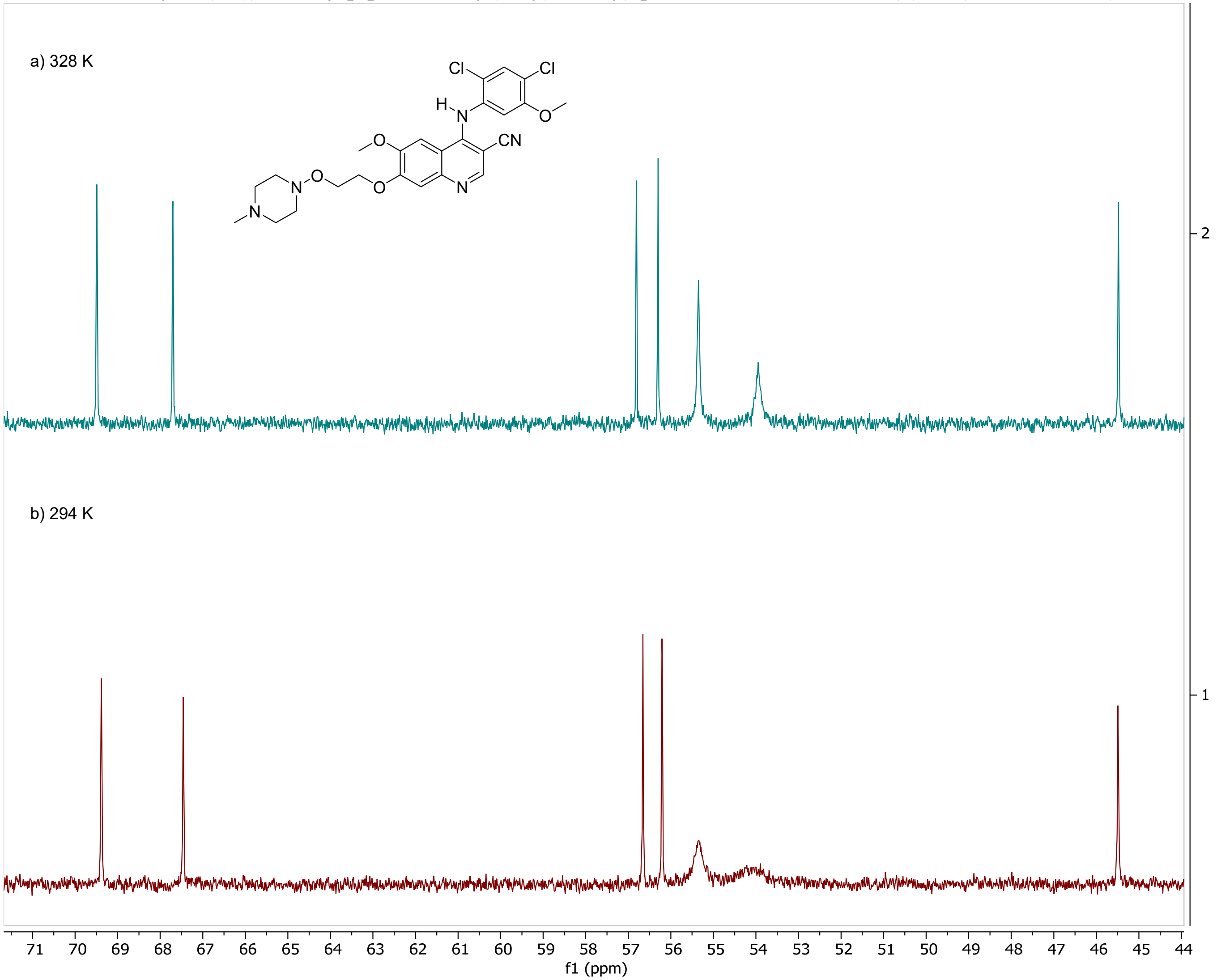

HSQC NMR (500 MHz) spectrum of 4-((2,4-dichloro-5-methoxyphenyl)amino)-6-methoxy-7-(2-((4-methylpiperazin-1-yl)oxy)ethoxy)quinoline-4-carbonitrile (**9**).

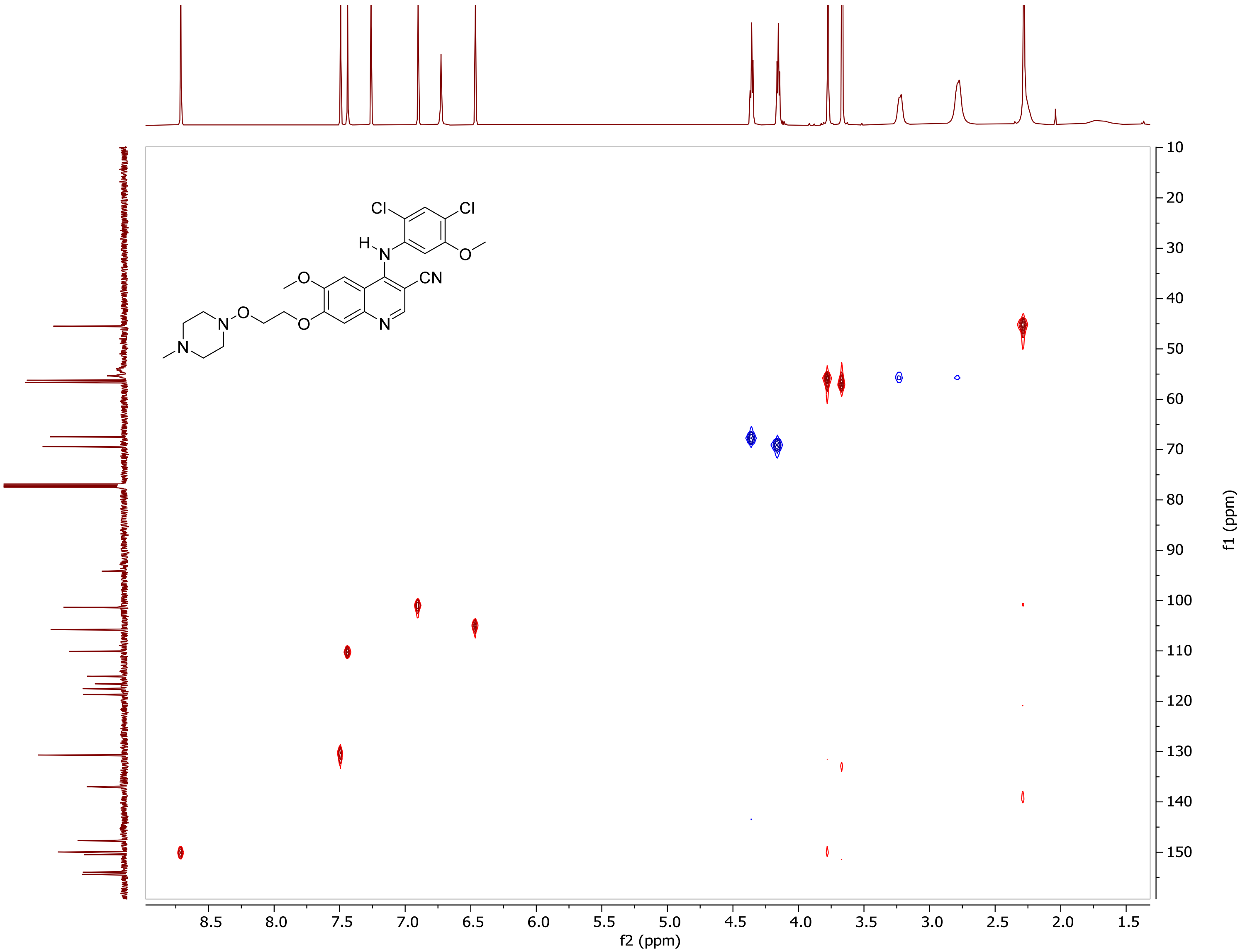

DQF COSY NMR (500 MHz) spectrum of 4-((2,4-dichloro-5-methoxyphenyl)amino)-6-methoxy-7-(2-((4-methylpiperazin-1-yl)oxy)ethoxy)quinoline-4-carbonitrile (**9**).

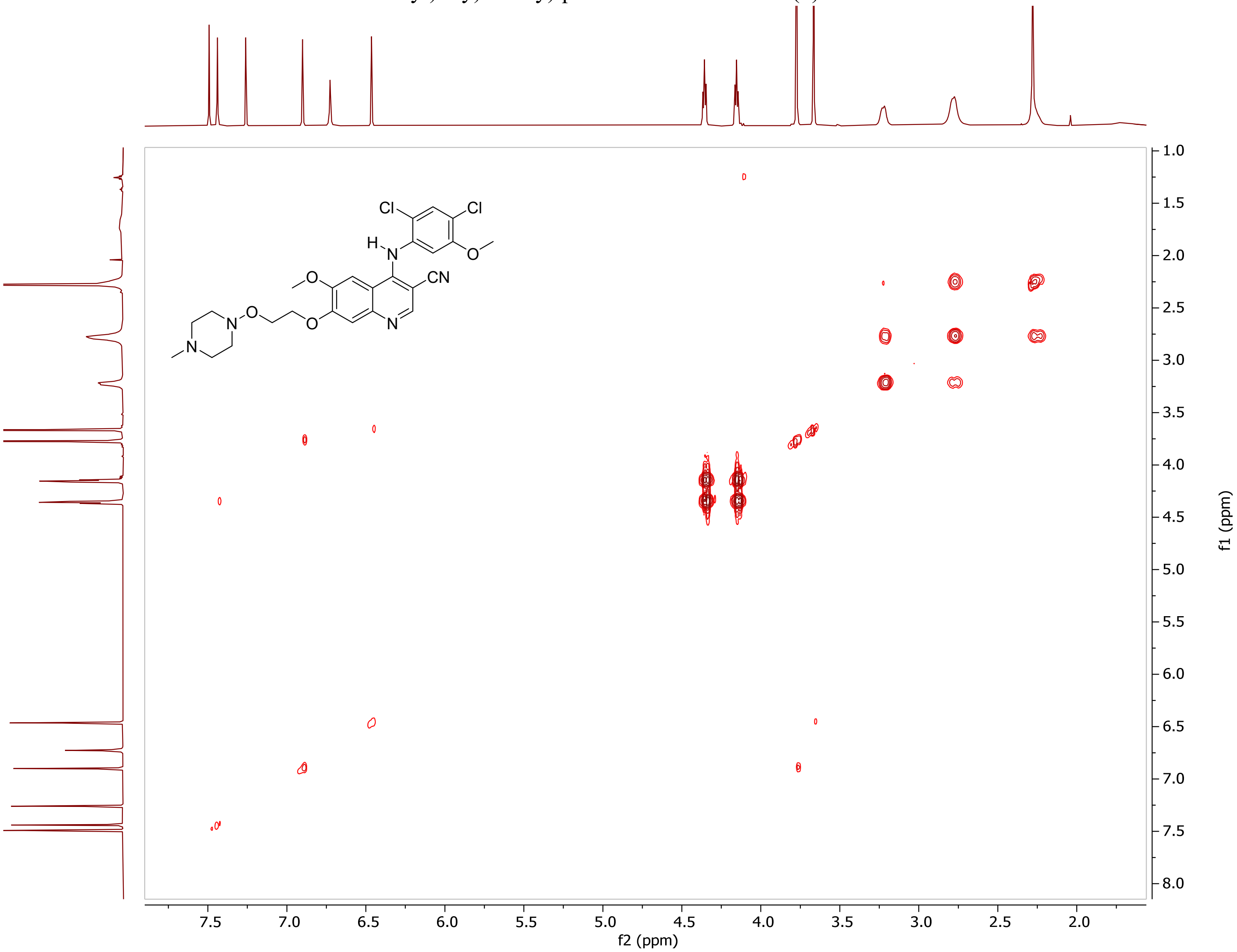

[1]**H NMR** (500 MHz, $CDCl_3$) spectrum of 3-(2-methoxyphenoxy)propyl-4-methylbenzenesulfonate (**22**).

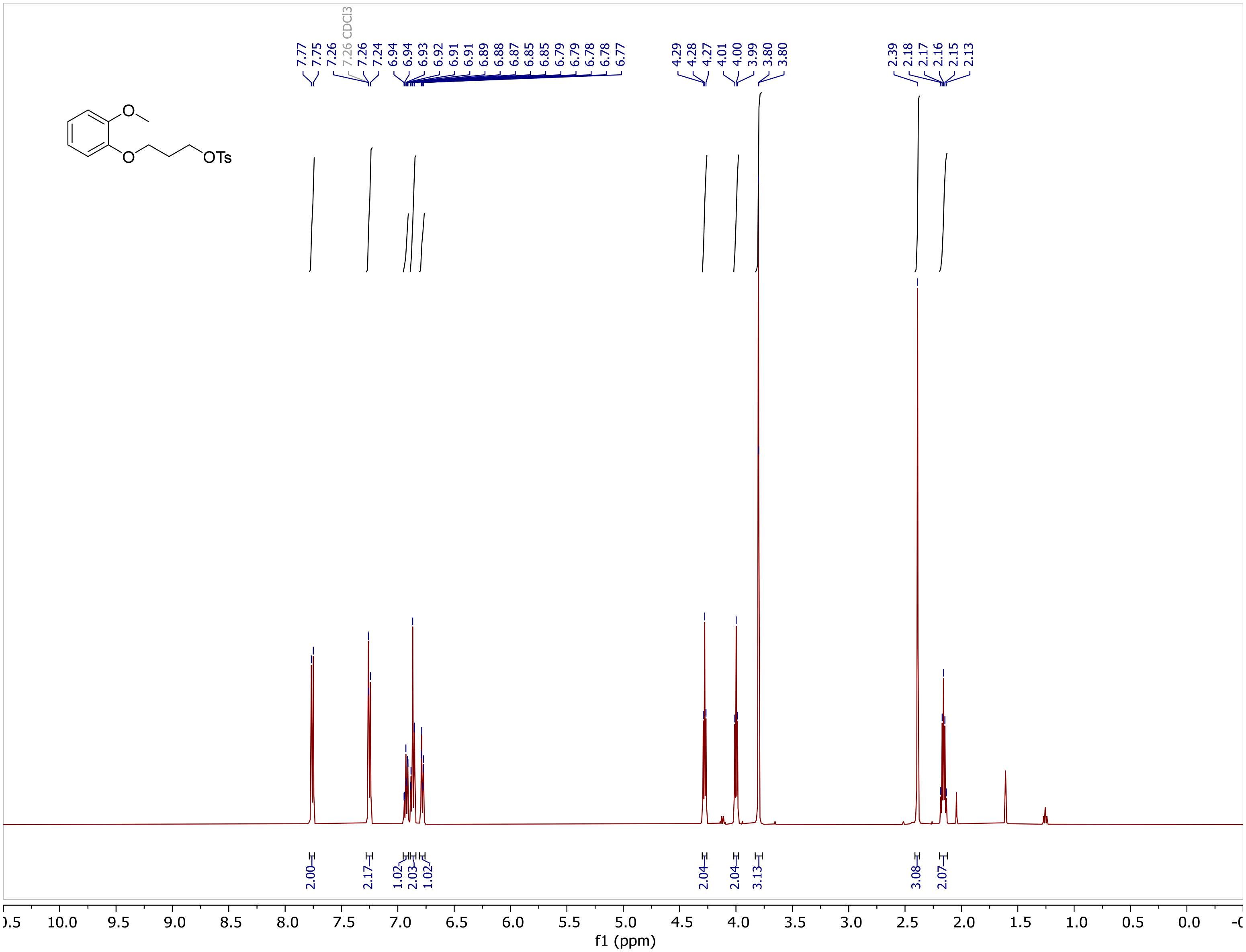

^{13}C NMR (126 MHz, $CDCl_3$) spectrum of 3-(2-methoxyphenoxy)propyl-4-methylbenzenesulfonate (**22**).

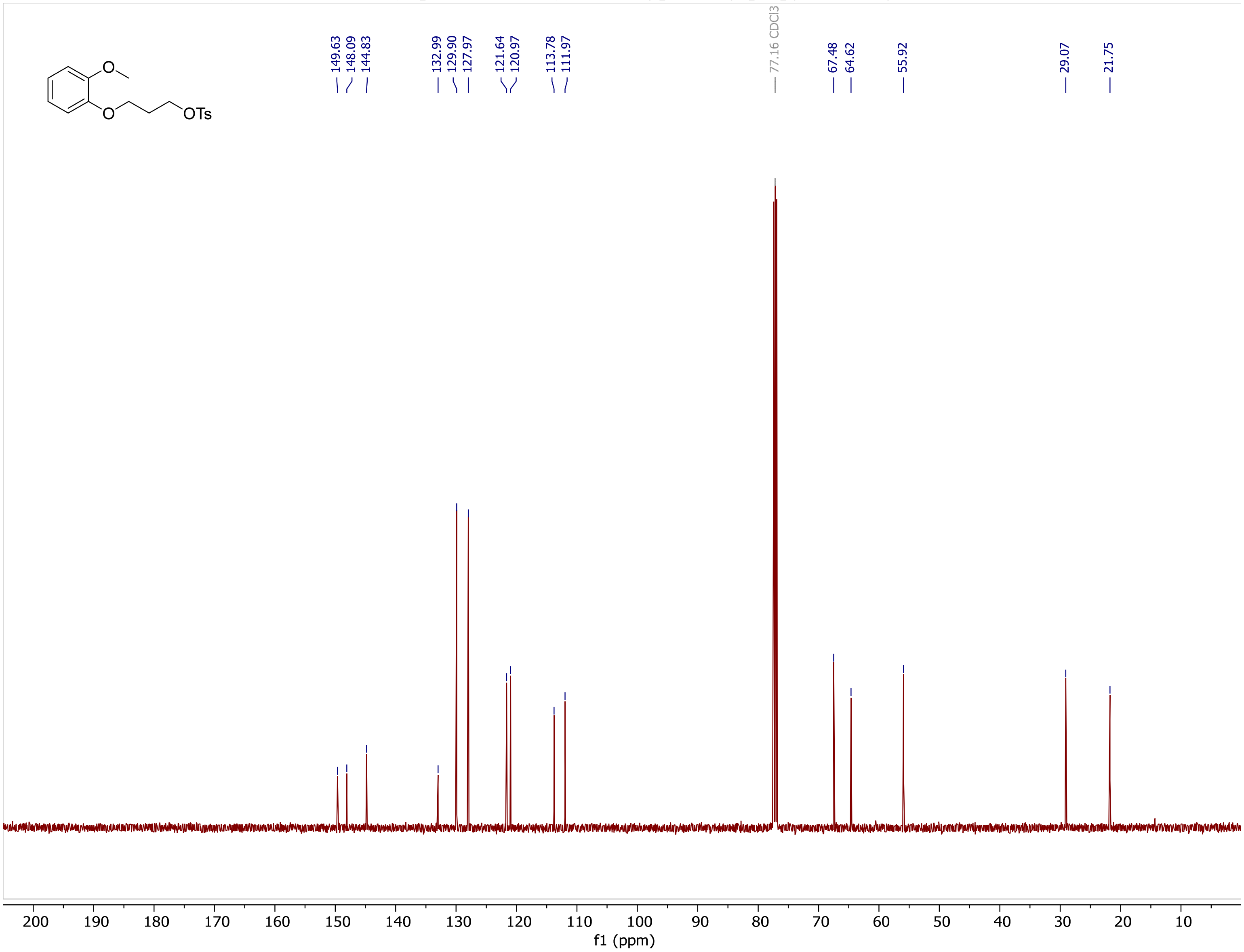

HSQC NMR (500 MHz, $CDCl_3$) spectrum of 3-(2-methoxyphenoxy)propyl-4-methylbenzenesulfonate (**22**).

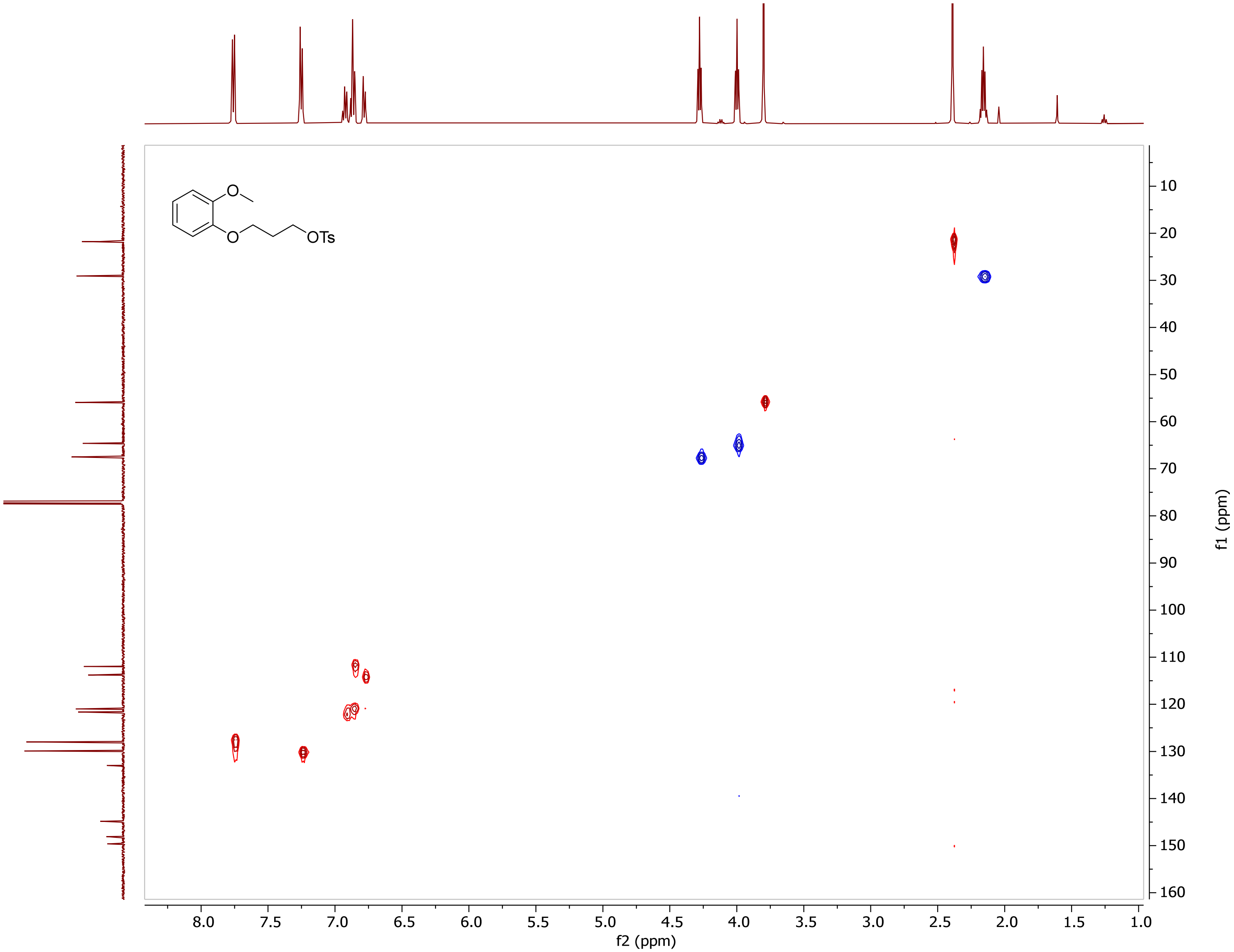

^{1}H NMR (500 MHz, $CDCl_3$) spectrum of 1-(3-(2-methoxyphenoxy)propyl)-4-methylpiperazine (**17**).

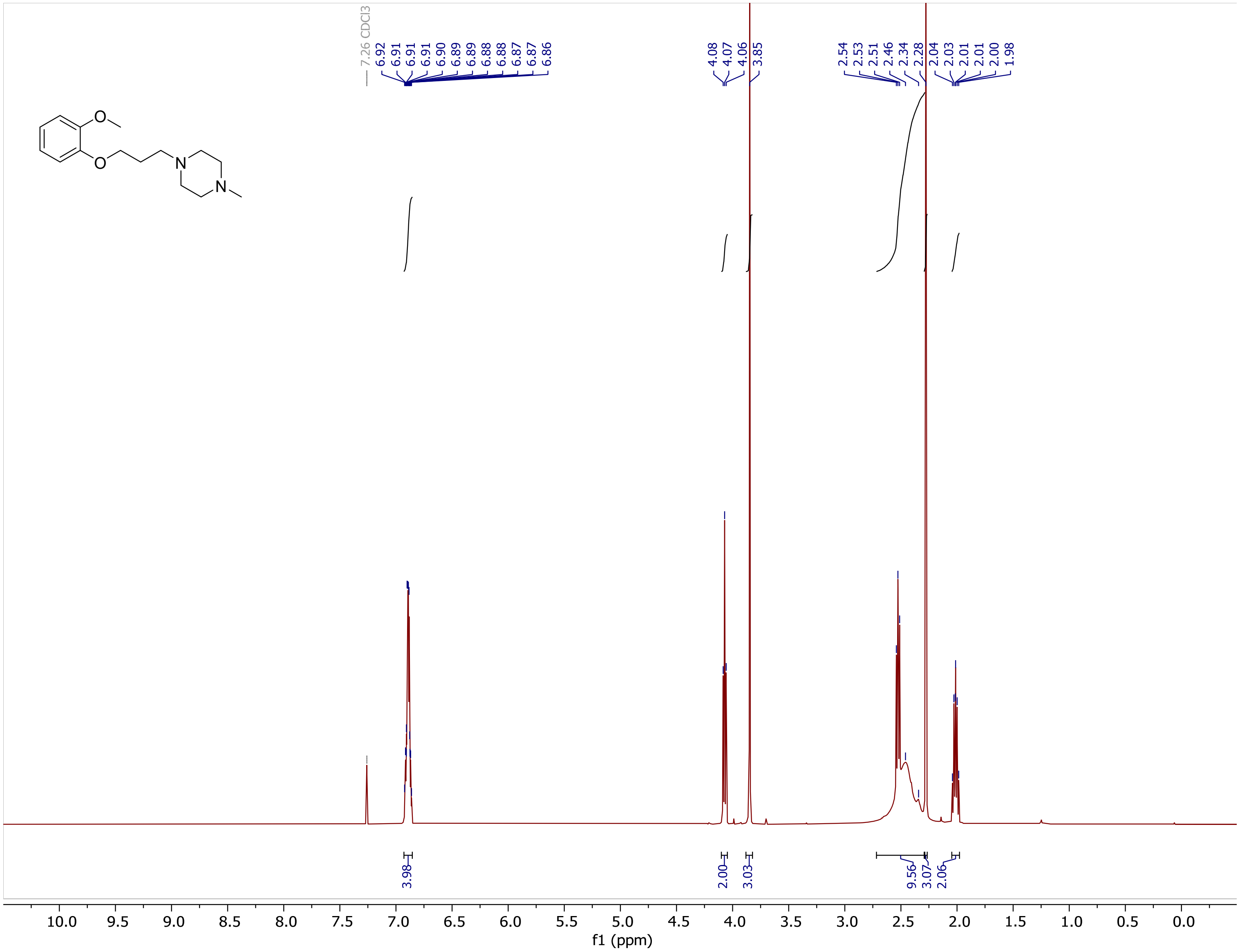

^{13}C NMR (126 MHz, $CDCl_3$) spectrum of 1-(3-(2-methoxyphenoxy)propyl)-4-methylpiperazine (**17**).

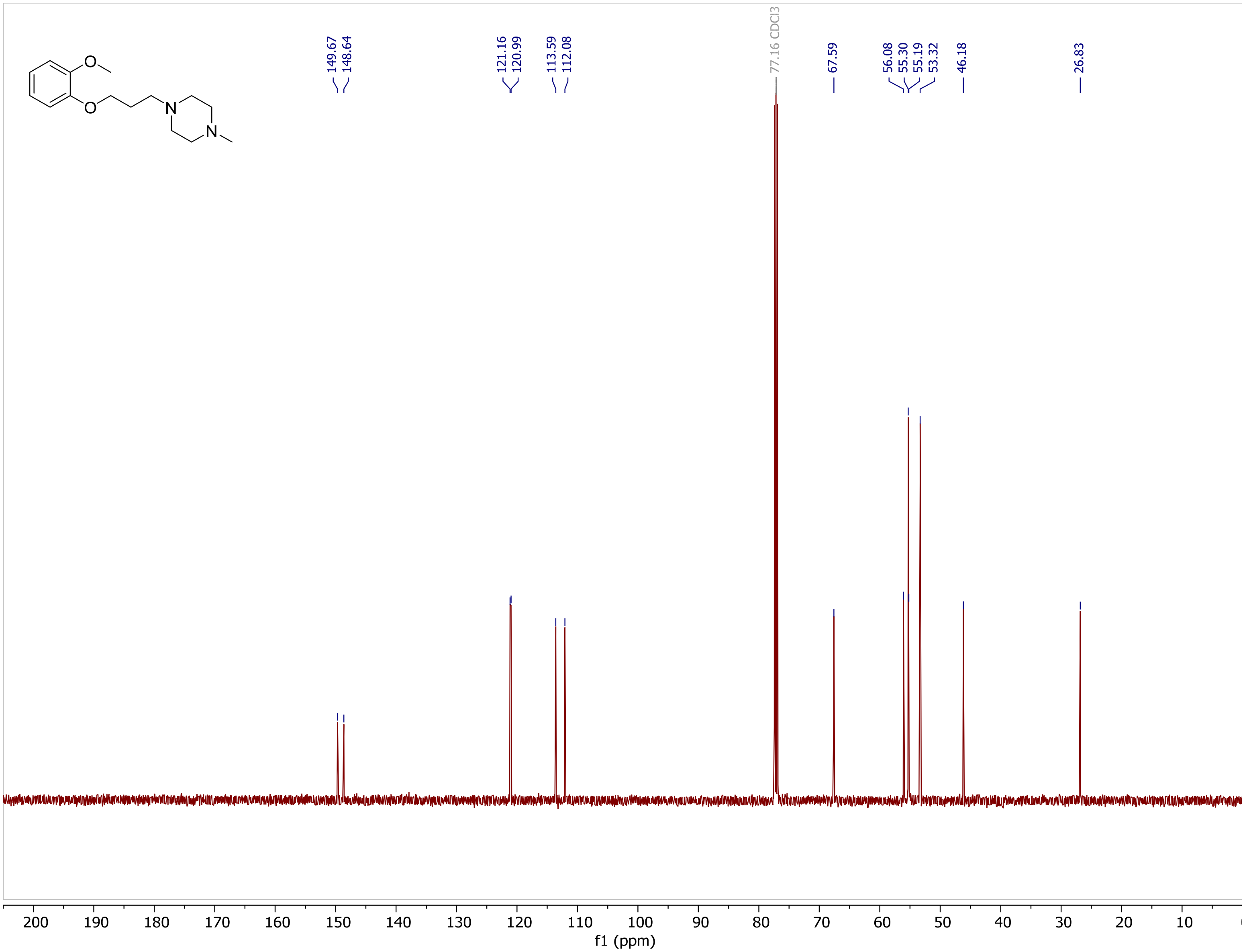

HSQC NMR (500 MHz, $CDCl_3$) spectrum of 1-(3-(2-methoxyphenoxy)propyl)-4-methylpiperazine (**17**).

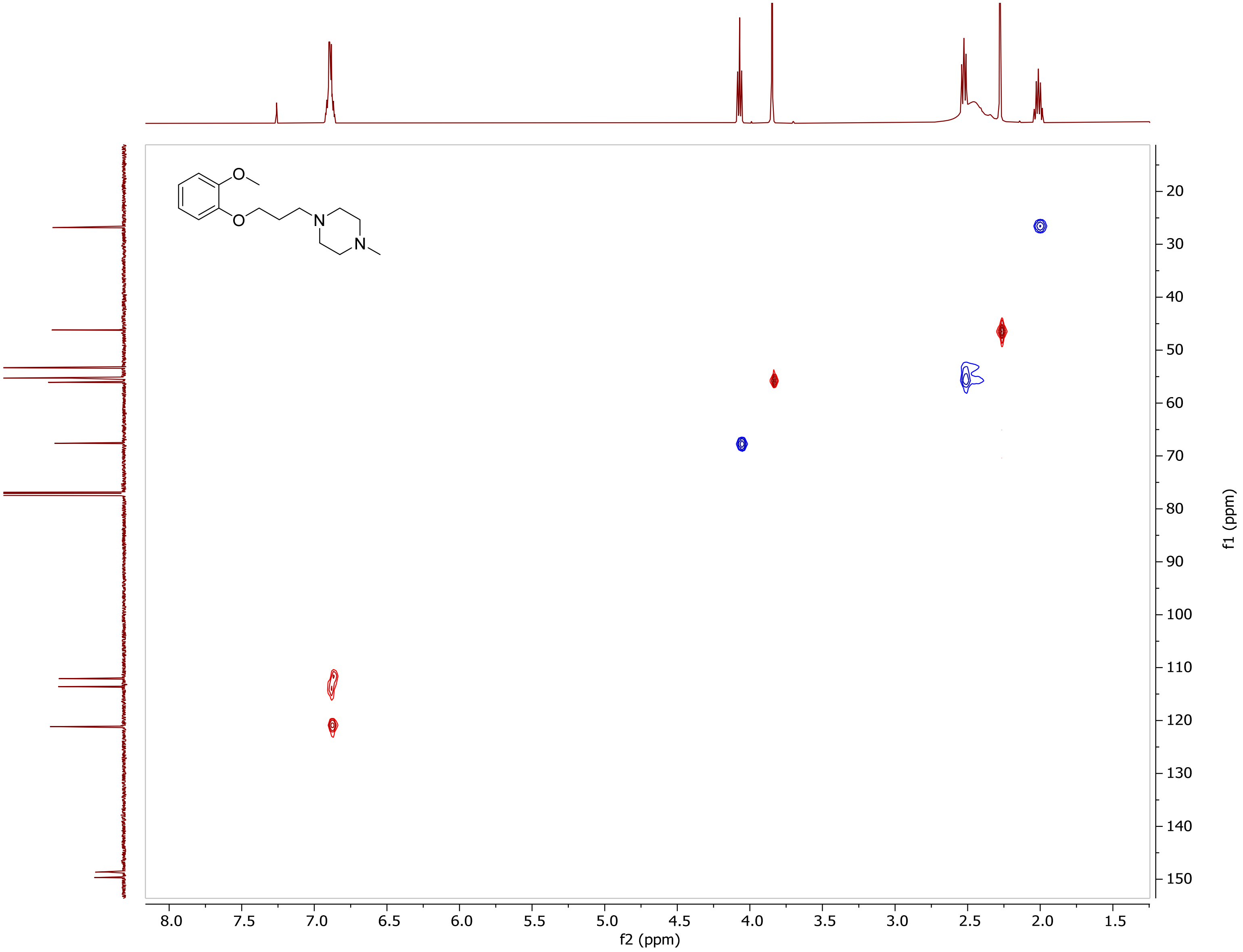

DQF-COSY NMR (500 MHz, $CDCl_3$) spectrum of 1-(3-(2-methoxyphenoxy)propyl)-4-methylpiperazine (**17**).

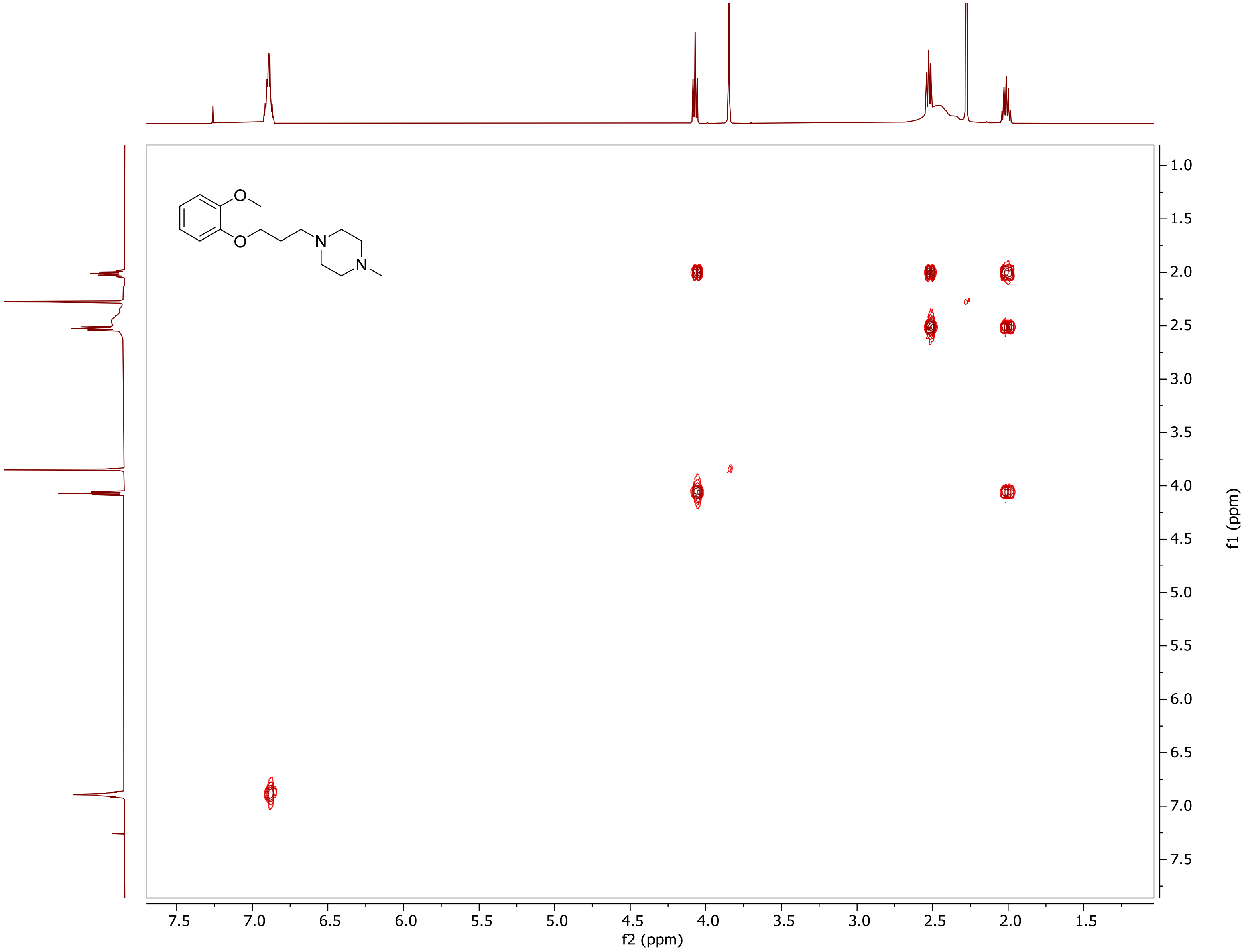

^{1}H NMR (500 MHz, $CDCl_3$) spectrum of 1-(2-(2-methoxyphenoxy)ethoxy)-4-methylpiperazine (**18**).

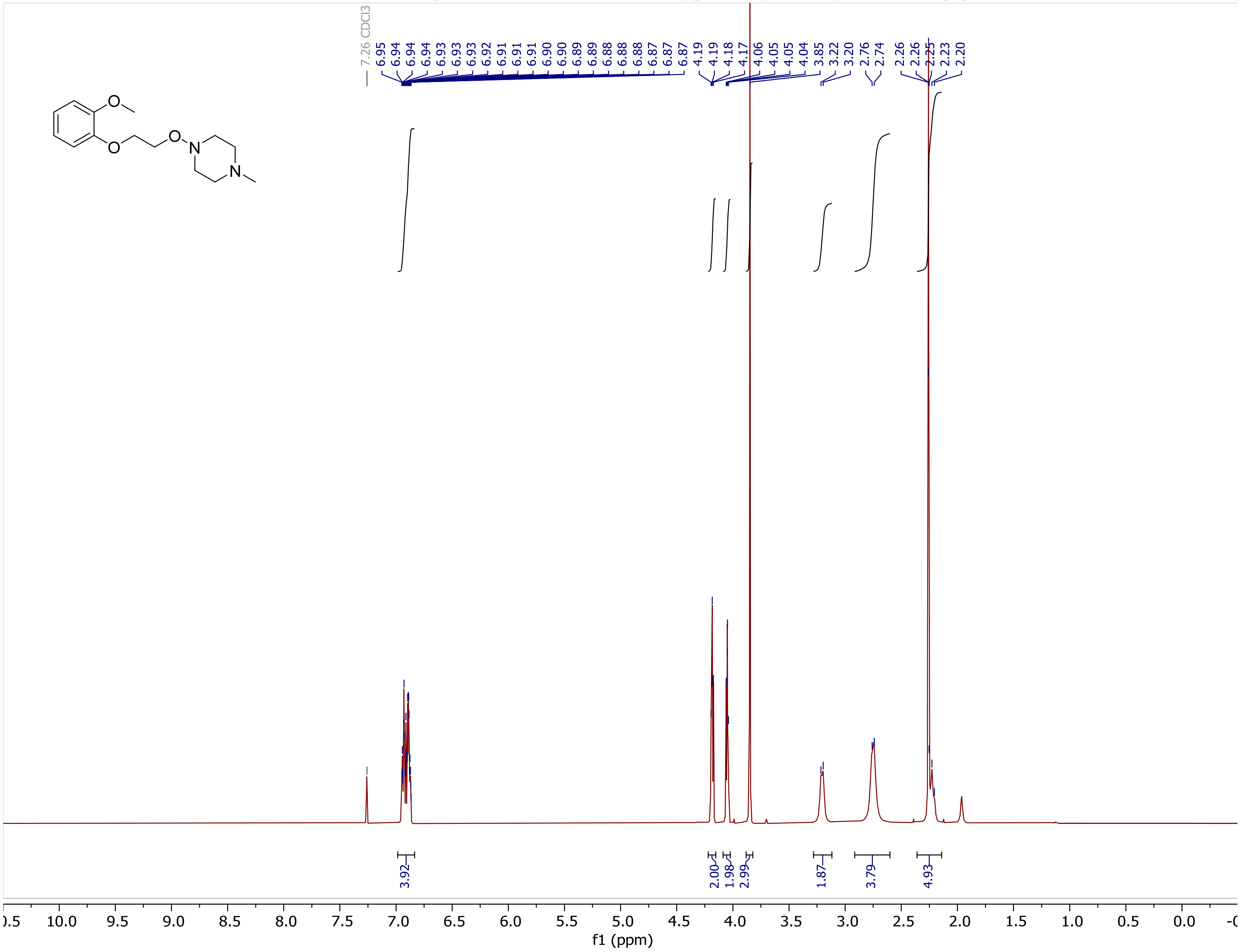

^{13}C NMR (126 MHz, $CDCl_3$) spectrum of 1-(2-(2-methoxyphenoxy)ethoxy)-4-methylpiperazine (**18**).

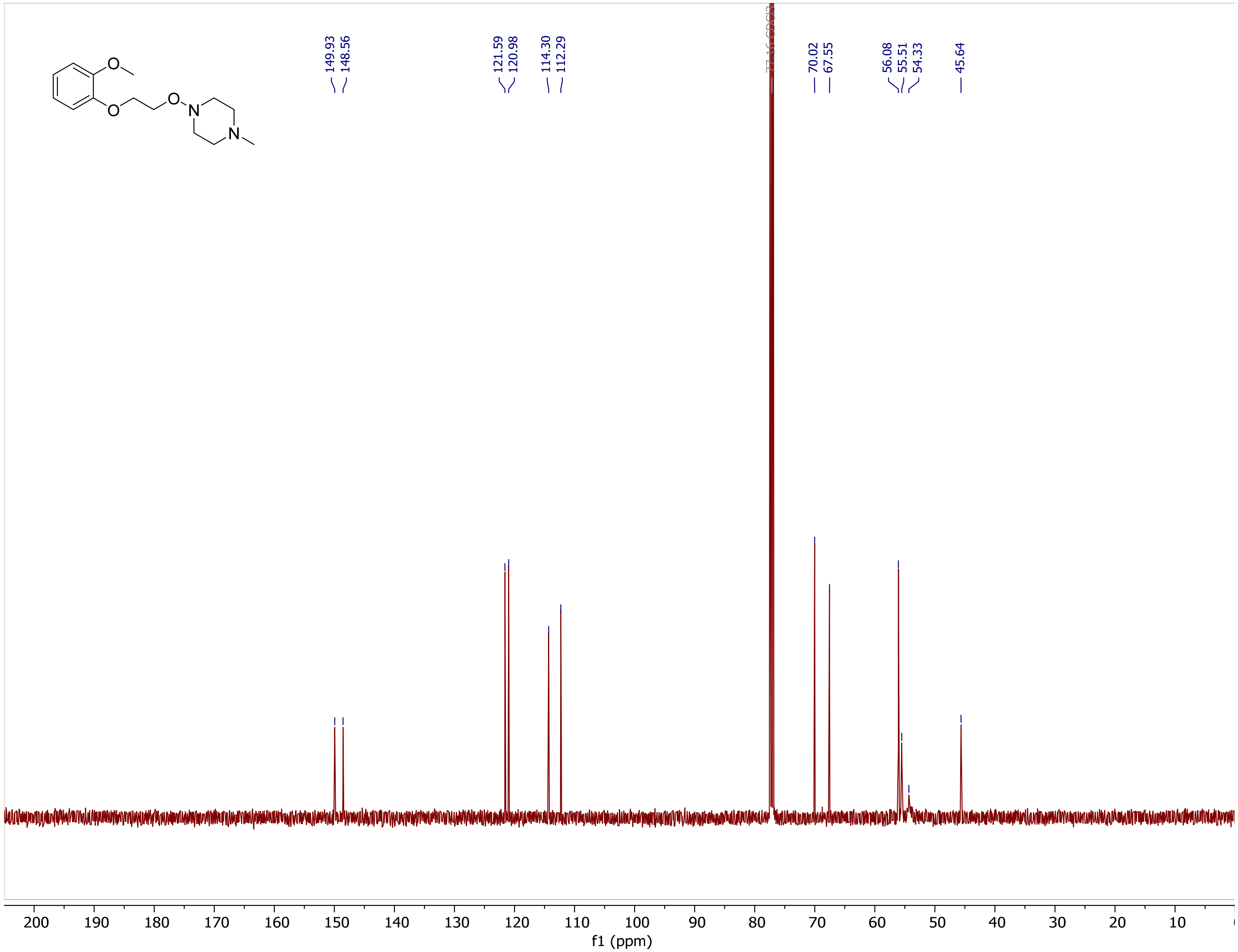

Expanded region of stacked variable temperature **^{13}C NMR** (126 MHz, $CDCl_3$) spectrum of 1-(2-(2-methoxyphenoxy)ethoxy)-4-methylpiperazine (**18**) at a) 328 K and b) 298 K.

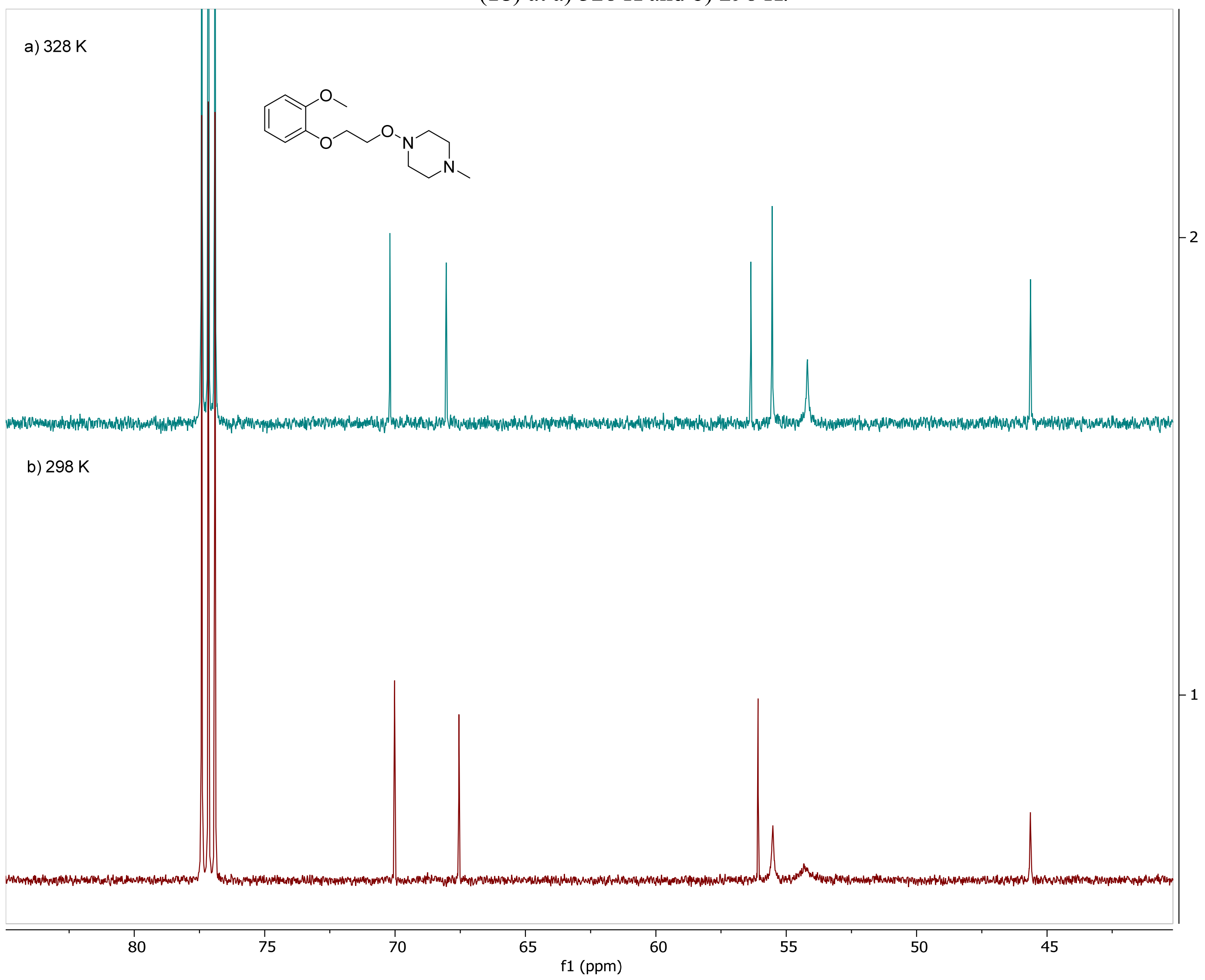

HSQC NMR (500 MHz, $CDCl_3$) spectrum of 1-(2-(2-methoxyphenoxy)ethoxy)-4-methylpiperazine (**18**).

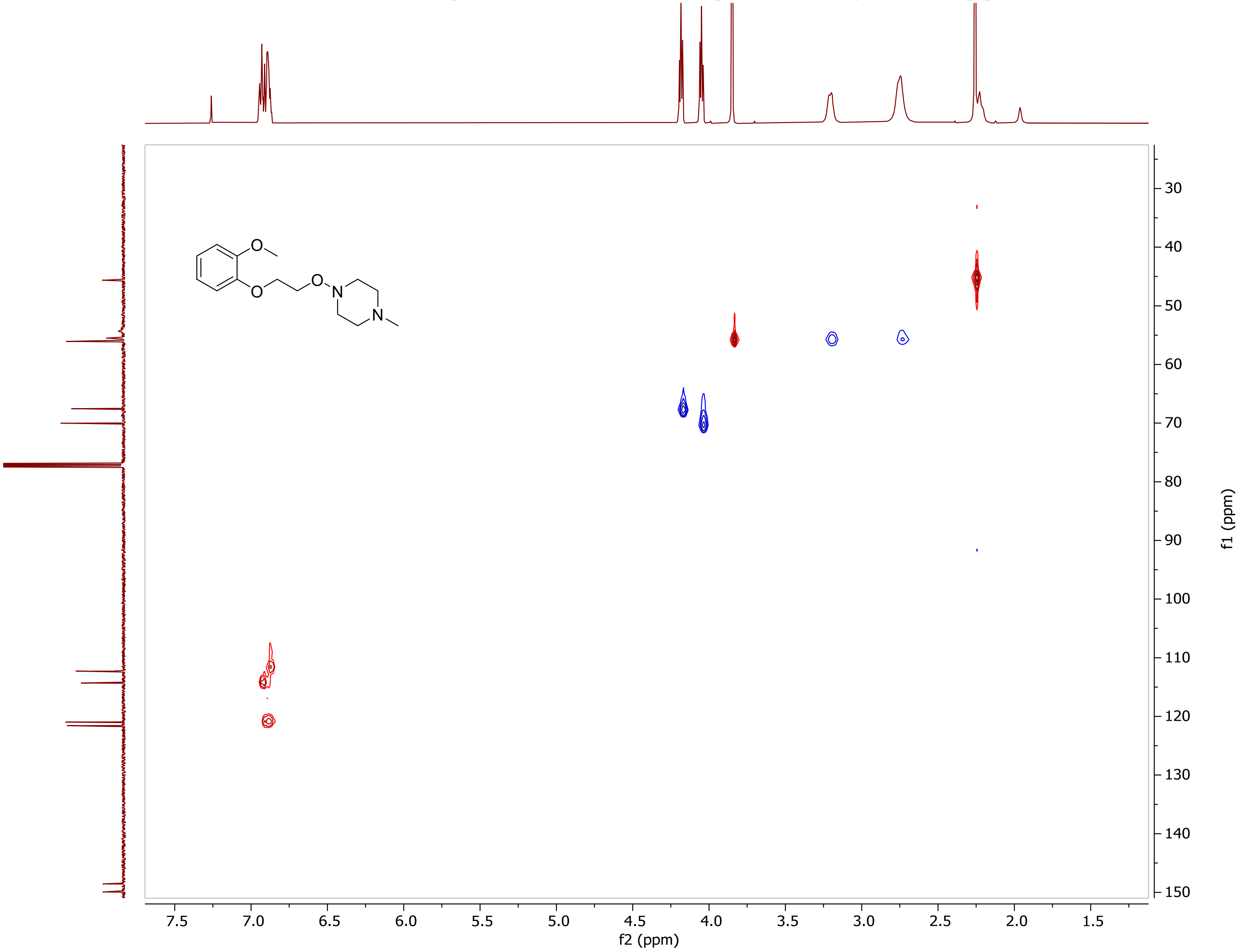

DQF-COSY NMR (500 MHz, $CDCl_3$) spectrum of 1-(2-(2-methoxyphenoxy)ethoxy)-4-methylpiperazine (**18**).

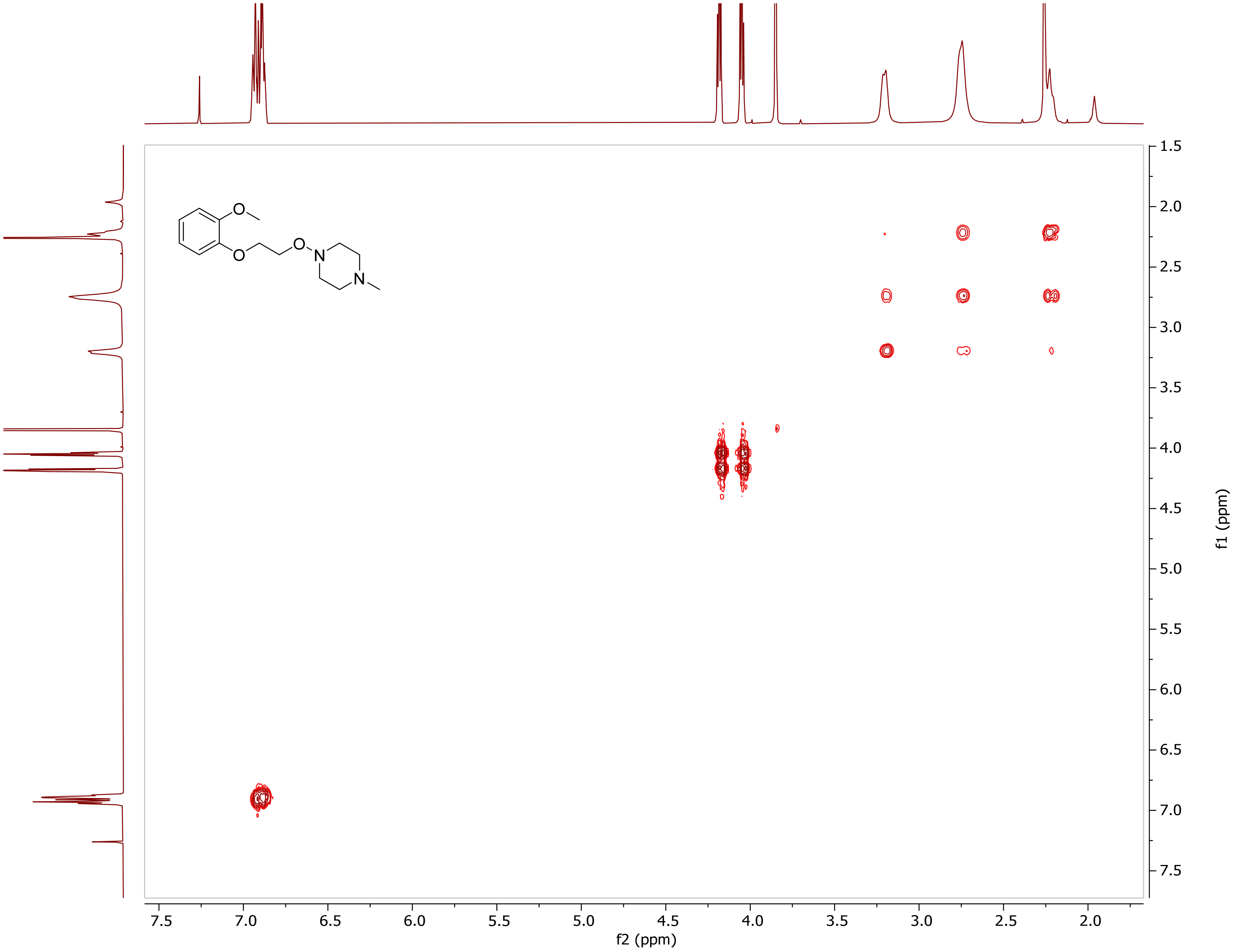

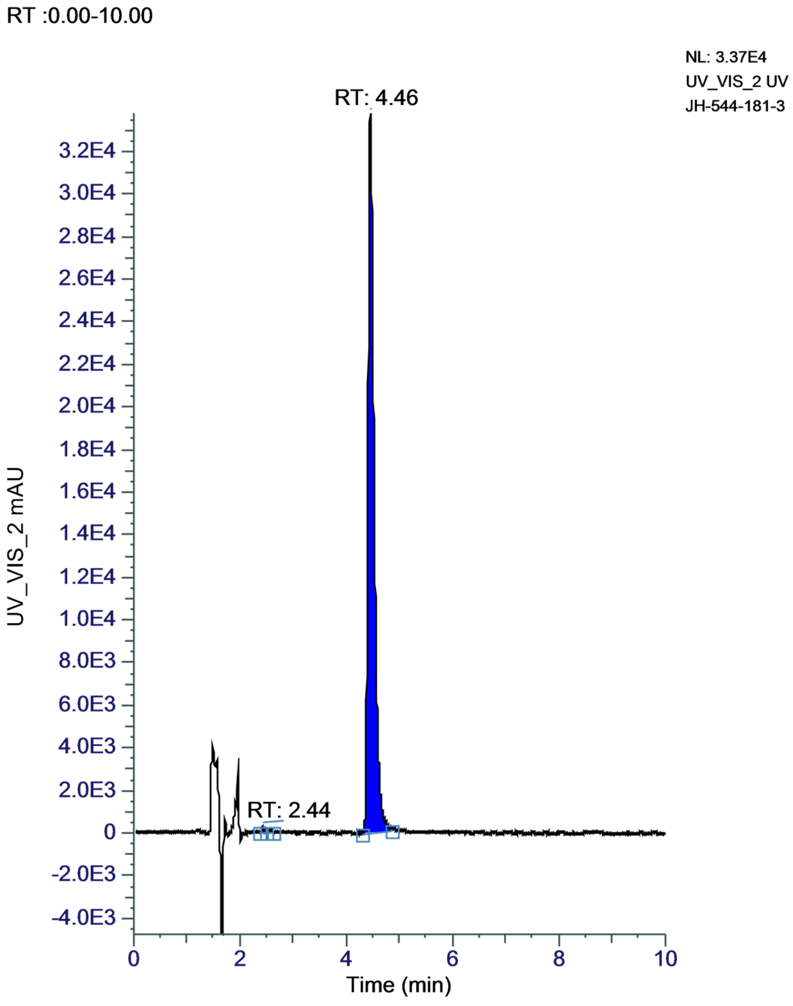

Figure S7. UHPLC trace of **9**. UHPLC/UV area percent purity of compound **9** at UV = 254 nm was determined to be 99.5% using automated Avalon peak area algorithm (peak list below).

Table S27. Peak List from Avalon peak area algorithm of compound **9** at UV = 254 nm.

Retention Time (rt) (min)	Start rt (min)	End rt (min)	Peak Area	Area (%)
2.44	2.38	2.52	9.981E+2	0.37
2.59	2.52	2.65	4.57E+2	0.17
4.46	4.31	4.87	2.708E+5	99.47

Table S28. HPLC method. Mobile Phase A: CH_3CN. Mobile phase B: 0.1 % (v/v) formic acid in ultrapure H_2O. Flow rate: 1.000 [$mL \cdot min^{-1}$], injection volume: 1.5 µL. Sample spiked with 0.1 mL formic acid.

Time (min)	Mobile Phase A (%)	Mobile Phase B (%)
0	30	70
10	30	70

6. References

1) Hill, J.; Jones, R. M.; Crich, D. Discovery of a hydroxylamine-based brain penetrant EGFR inhibitor for metastatic non-small-cell lung cancer. *J. Med. Chem.* **2023**, *66* (22), 15477-15492.

2) Hill, J.; Crich, D. The *N,N,O*-trisubstituted hydroxylamine isostere and its influence on lipophilicity and related parameters. *ACS Med. Chem. Lett.* **2022**, *13* (5), 799-806.

3) Hill, J.; Hettikankanamalage, A. A.; Crich, D. Diversity-oriented synthesis of *N,N,O*-trisubstituted hydroxylamines from alcohols and amines by N-O bond formation. *J. Am. Chem. Soc.* **2020**, *142* (35), 14820-14825.

4) Hoang, K. M.; Lees, N. R.; Herzon, S. B. Programmable synthesis of 2-deoxyglycosides. *J. Am. Chem. Soc.* **2019**, *141* (20), 8098-8103.

5) Kyasa, S.; Meier, R. N.; Pardini, R. A.; Truttmann, K.; Kuwata, K. T.; Dussault, P. H. Synthesis of ethers via reaction of carbanions and monoperoxyacetals. *J. Org. Chem.* **2015**, *80* (24), 12100-12114.

6) Riddell, F. G.; Turner, E. S. The barrier to rotation about the N-O bond. *J. Chem. Soc., Perkin Trans.* **1978**, *2*, 707-708.

7) Hassan, A.; Wazeer, M. I. M.; Perzanowski, H. P.; Ali, S. A. Nitrogen inversion and N-O bond rotation in some hydroxylamine and isoxazolidine derivatives. *J. Chem. Soc., Perkin Trans.* **1997**, *2*, 411-418.

8) Raban, M.; Kost, D. Stereolabile configurational units - Torsional and inversional stereochemistry in sulfenamides and hydroxylamines. *Tetrahedron* **1984**, *40* (18), 3345-3381.

9) Dhanju, S.; Blazejewski, B. W.; Crich, D. Synthesis of trialkylhydroxylamines by stepwise reduction of *O*-acyl *N,N*-disubstituted hydroxylamines. Substituent effects on the reduction of *O*-(1-acyloxyalkyl)hydroxylamines and on the conformational dynamics of *N*-alkoxypiperidines. *J. Org. Chem.* **2017**, *82* (10), 5345-5353.
